# Phosphorylation‐linked complex profiling identifies assemblies required for Hippo signal integration

**DOI:** 10.15252/msb.202211024

**Published:** 2023-03-10

**Authors:** Federico Uliana, Rodolfo Ciuffa, Ranjan Mishra, Andrea Fossati, Fabian Frommelt, Sabrina Keller, Martin Mehnert, Eivind Salmorin Birkeland, Frank van Drogen, Nevena Srejic, Matthias Peter, Nicolas Tapon, Ruedi Aebersold, Matthias Gstaiger

**Affiliations:** ^1^ Department of Biology Institute of Molecular Systems Biology, ETH Zurich Zurich Switzerland; ^2^ Department of Biology Institute of Biochemistry, ETH Zurich Zurich Switzerland; ^3^ Quantitative Biosciences Institute (QBI) University of California San Francisco San Francisco CA USA; ^4^ Department of Cellular and Molecular Pharmacology University of California San Francisco San Francisco CA USA; ^5^ J. David Gladstone Institutes San Francisco CA USA; ^6^ Apoptosis and Proliferation Control Laboratory The Francis Crick Institute London UK

**Keywords:** Interaction proteomics, Protein phosphorylation, Protein–protein interactions, YAP1, Proteomics, Signal Transduction

## Abstract

While several computational methods have been developed to predict the functional relevance of phosphorylation sites, experimental analysis of the interdependency between protein phosphorylation and Protein–Protein Interactions (PPIs) remains challenging. Here, we describe an experimental strategy to establish interdependencies between protein phosphorylation and complex formation. This strategy is based on three main steps: (i) systematically charting the phosphorylation landscape of a target protein; (ii) assigning distinct proteoforms of the target protein to different protein complexes by native complex separation (AP‐BNPAGE) and protein correlation profiling; and (iii) analyzing proteoforms and complexes in cells lacking regulators of the target protein. We applied this strategy to YAP1, a transcriptional co‐activator for the control of organ size and tissue homeostasis that is highly phosphorylated and among the most connected proteins in human cells. We identified multiple YAP1 phosphosites associated with distinct complexes and inferred how both are controlled by Hippo pathway members. We detected a PTPN14/LATS1/YAP1 complex and suggest a model how PTPN14 inhibits YAP1 via augmenting WW domain‐dependent complex formation and phosphorylation by LATS1/2.

## Graphical abstract

A novel approach is proposed to study the functional relationships between two critical mechanisms in cell signaling: PTMs and complex formation. This proteomic workflow is applied to investigate YAP1, a major signal integrator and effector of the Hippo pathway.

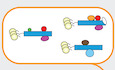

## Introduction

Two of the central principles of cell signaling regulation are the state‐specific remodeling of protein assemblies and the site‐specific modification (posttranslational modifications—PTMs) of signaling proteins, particularly phosphorylation (Pawson & Scott, 2005; Scott & Pawson, 2009). Mass spectrometry represents the method of choice to analyze both Protein–Protein Interactions (PPIs) and protein phosphorylation with high throughput, dynamic range, accuracy, and sensitivity (Bensimon *et al*, 2012; Gstaiger & Aebersold, 2013; Olsen & Mann, 2013; Riley & Coon, 2016). While affinity purification coupled to mass spectrometry (AP‐MS) has traditionally been the method of choice for the identification of PPIs, newer methods have emerged that specifically identify proximal proteins (e.g., BioID; Lambert *et al*, 2015) as well as groups of proteins co‐separating under native conditions, therefore suggesting protein complexes (protein correlation profiling, PCP; Havugimana *et al*, 2012; Heusel *et al*, 2019). For phosphorylation, phosphopeptide enrichment strategies have compensated for the frequently substoichiometric nature of these peptides, and state‐of‐the art efforts are routinely capable of quantifying thousands of different sites (Bekker‐Jensen *et al*, 2020). However, protein phosphorylation and protein interactions are not independent events, but rather represent two, frequently causally interdependent aspects of the same regulatory system. In most signaling studies, these two aspects are dealt with distinct experimental and computational settings, hence separating two key facets of the cellular regulatory networks. Affinity enrichment of a proteins of interest can partly alleviate sensitivity issues of global phosphoproteomics studies; however, both AP‐MS and proximity labeling provide a convoluted representation of interactions and phosphosites of concurrently purified complexes (Zheng *et al*, 2013; Lundby *et al*, 2019). Therefore, these methods fail to provide association between phospho‐proteoforms and complex formation. Here, we present an approach to separate and identify complex isoforms of a protein and its complex‐specific phosphosites.

The intersection of literature information of human PPIs with the most recent survey of functional phosphosites (Ochoa *et al*, 2020) identifies a small subset of proteins that are both interaction hubs and strongly regulated at the posttranslational level. YAP1 stands out as a unique example because it (i) is a promiscuous interactor (top 1% in terms of number of known interactors; Oughtred *et al*, 2019); (ii) carries a high number of identified phosphorylation events and functional phosphosites (Hornbeck *et al*, 2015; Ochoa *et al*, 2020; only second to p53 among top 1% promiscuous interactors); and (iii) has a well‐characterized signaling role. For these reasons, we chose YAP1 in our study as a model to establish and apply a robust workflow to determine the interdependencies between phosphorylation and PPI formation (Appendix Fig S1A). Furthermore, YAP1 is best known as a main effector of the Hippo pathway, a conserved signaling cascade that regulates tissue homeostasis and organ size. The core of the Hippo pathway is a kinase module of the Mammalian STE20‐like 1/2 (STK 3/4) and Large tumor suppressor 1/2 (LATS1/2), kinases that target YAP1, and a second transcriptional co‐activator, TAZ. Once the pathway is activated, STK3/4 phosphorylates LATS1/2, thus promoting the activation of the kinase and consequent YAP1 phosphorylation. Phosphorylation events in YAP1 reduce its nuclear localization and binding to the TEAD family of transcription factors, thereby blocking the transcription of genes involved in cell proliferation, apoptosis, and differentiation (Zhao *et al*, 2008; Yu & Guan, 2013).

Our current knowledge on the role of YAP1 phosphorylation in Hippo signaling is largely based on a limited set of widely available phosphorylation‐specific antibodies. For instance, *Cell Signaling Technologies* reports antibodies for only four of 52 sites cataloged in the *Phosphositeplus* repository (Hornbeck *et al*, 2015). However, this protein phosphorylation database suggests a significant number of additional YAP1 phosphorylation sites that at present are functionally unexplored. This bias is well‐exemplified by the site S127, which makes up around 50% of all low‐throughput studies entry in the *Phosphositeplus* repository, but only around 10% of the high‐throughput studies entries (Hornbeck *et al*, 2015; He *et al*, 2016; Appendix Fig S1B).

**Figure 1:**
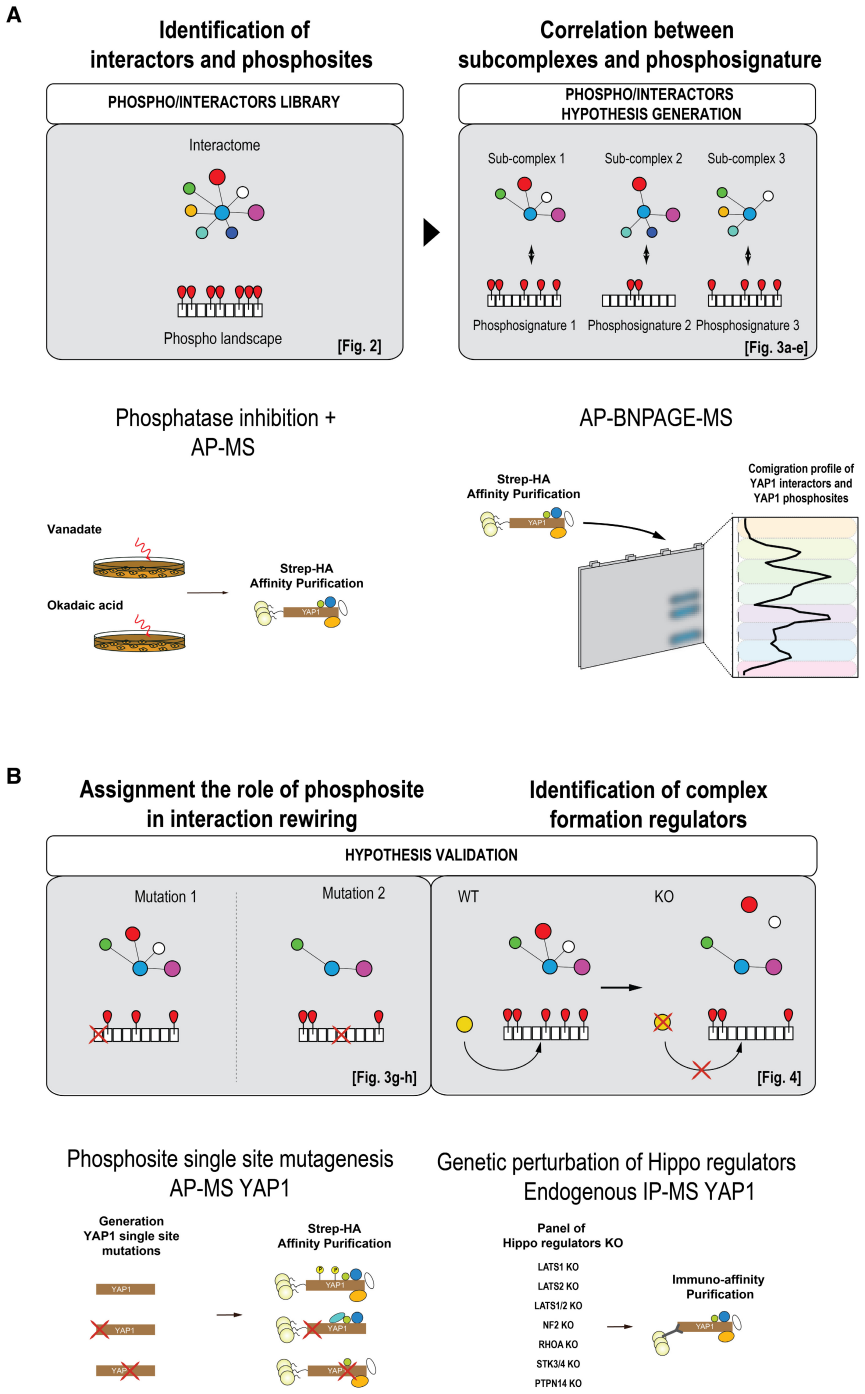
Study design Systematic dissection of phosphorylation‐dependent YAP1 complex re‐organization. First, YAP1 interactors and phosphosites are identified and quantified in steady‐state and upon perturbation with phosphatase inhibitors (left). In this experiment, ectopically expressed Strep‐HA YAP1 is purified by Strep tag and analyzed by mass spectrometry to generate a library of phosphosites and interactors. In a second step (right), YAP1 interactors and phosphosites identified in the previous experiment are separated by AP‐BNPAGE and the physically distinct modules associated to YAP1 phosphosignatures are identified by co‐migration profile.Finally, phopho‐interactors associations are validated by the quantification of YAP1 interactors after the purification of Strep‐HA YAP1 phosphosite‐null mutants (left) and by the investigation of endogenous YAP1 interactors and phosphosites in a panel of genetic deletions of known YAP1 regulators (right).

In this work, we develop an integrated multilayered proteomic workflow to study the interdependencies between protein phosphorylation and PPIs (Fig 1A and B). In a first step, we combine cellular phosphatase inhibition with AP‐MS to comprehensively map the extent and plasticity of YAP1 phosphorylation in treated and untreated cells and to determine impact of phosphorylation on YAP1 protein interactions. In a second step, we separate affinity purified YAP1 complexes by Blue Native PAGE (AP‐BNPAGE) and characterize by MS the composition of different YAP1 modules and YAP1 phosphorylation state (we use here the term “module” to refer to a group of co‐migrating proteins, and “complex” to refer to physically stable assemblies). To test the functional role of identified phosphosites in complex formation, we analyze a panel of YAP1 phosphosite mutants. This results in a map of phospho‐dependent interactions, confirming the role of some sites in the regulation of specific complexes. Finally, we apply targeted proteomics to immuno‐affinity purified, endogenous YAP1 complexes to quantify phosphorylation sites, and interactors detected in the previous steps in a panel of cell lines with genetic deletion of Hippo pathway members previously linked to the regulation of YAP1 activity. The results, besides to confirm prior knowledge, they provide new molecular understanding of the impact of LATS1/2, RHOA and NF2 on YAP1 regulation, and identify the nonreceptor tyrosine phosphatase 14 (PTPN14) as an important noncanonical regulator of YAP1 function. Indeed, our data show that the formation of a YAP1‐LATS1/2 complex and subsequent YAP1 phosphorylation requires the presence of PTPN14. Furthermore, we show that LATS1 and PTPN14 interact directly with each other and bind via the two WW domains of YAP1, with LATS1 exhibiting preference for first and PTPTN14 for the second WW domain. We thus propose a model where PTPN14 controls YAP1 activity by facilitating LATS‐YAP1 complex formation and subsequent LATS‐dependent phosphorylation which, in turn, controls YAP1 complex organization. In summary, we establish a generic method that systematically dissects PTMs (phosphorylation)‐dependent complex formation as a promising avenue to understand signaling mechanisms of proteins that similar to YAP1, act as key integrator and effectors of diverse signaling inputs.

## Results

### Plasticity of the phosphorylation‐dependent YAP1 interactome

**Figure 2:**
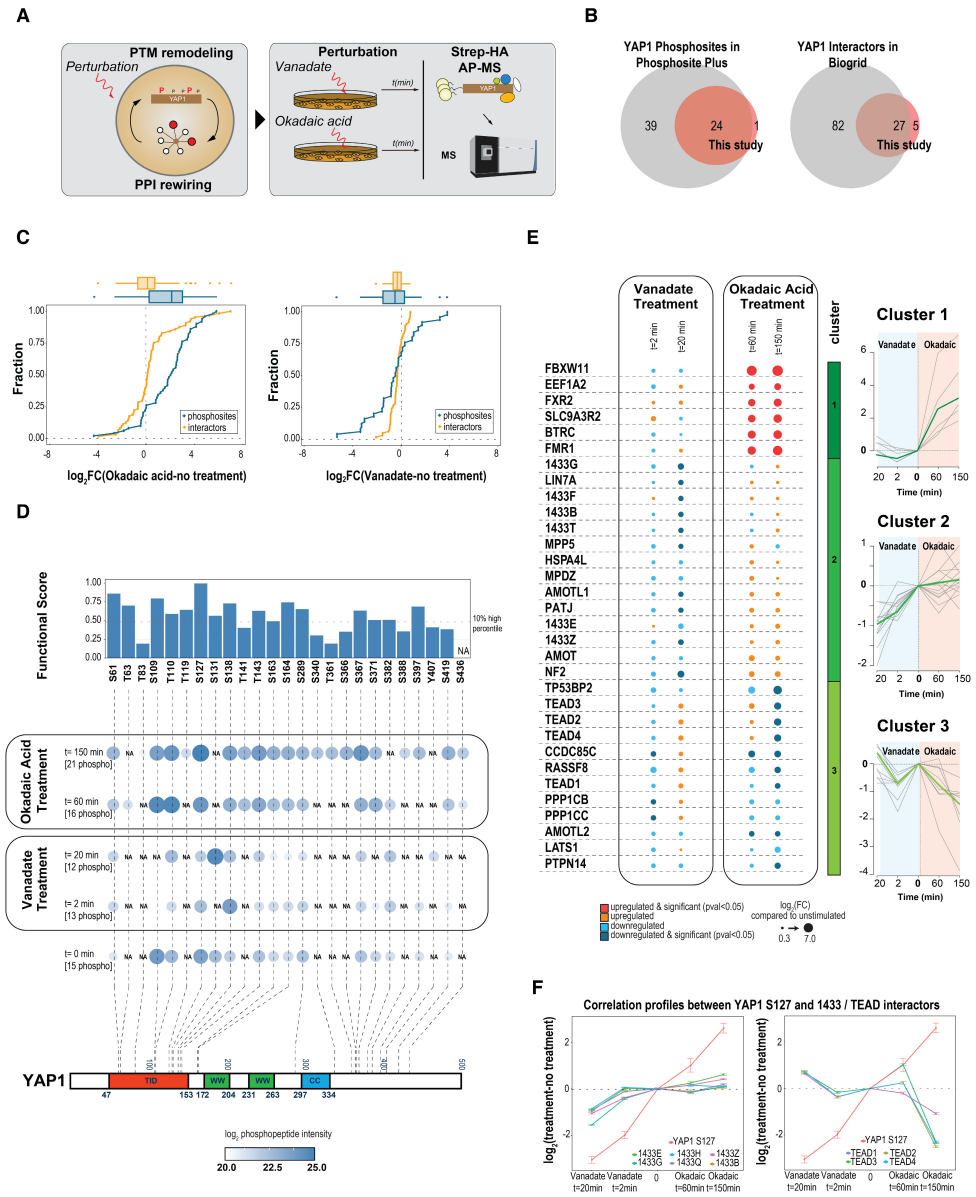
Plasticity of the phosphorylation‐dependent YAP1 interactome AP‐MS approach to profile Strep‐HA‐tagged YAP1 phosphorylation changes and interactome rewiring after phosphatase inhibitor treatment.Overlap of identified and annotated phosphosites (left) and interactors (right) between this study and reference databases. Phosphosites cataloged in *Phosphositeplus* (CST) and YAP1 interactor annotated in BioGRID as “physical and direct interactor” in at least two independent experiments were considered in the reference databases.Empirical cumulative density function (ECDF plot) for YAP1 interactors and phosphosites after stimulation with okadaic acid (left) and vanadate (right). *x* axis represents the log2FC for the respective perturbation versus the control sample (untreated).Kinetics of YAP1 phosphorylation sites. After treatment with vanadate (2 and 20 min) and okadaic acid (60 and 150 min), YAP1 phosphosite abundance is measured by MS preceded by Strep affinity purification. Only YAP1 phosphosites identified in all replicates (three independent replicates) and with a localization score higher than 0.8 are considered for this analysis. Quantification analysis is performed based on the average of MS1 peptide signal intensity. Size and color of circles represent the average intensity of phosphosites normalized for YAP1 intensity. Barplot on top indicates the functional score associated with each site as reported in Ochoa *et al* (2020). On the bottom, phosphosites are localized onto YAP1 primary sequence (lower part).Kinetics of YAP1 interactors upon phosphatase treatment. After treatment with vanadate (2 and 20 min) and okadaic acid (60 and 150 min), YAP1 interactors are identified by MS. Only high confidence YAP1 interactors (SAINT SP > 0.9) are considered for quantitative analysis based on MS1 peptide intensity. The dot size represents log2 fold change from three independent experiments compared to non‐treated samples. Color scale emphasizes the significance of the changes (two‐sided unpaired Student's *t*‐test). Interactors are fuzzy‐clustered based on the kinetic profile of fold change compared to no treatment condition (right panel).Correlation profiles between YAP1 S127 phosphosite with 14‐3‐3 protein family (left) and with TEADs protein family (right). Mean and SE values of log2 fold change from three independent experiments compared with nontreated samples are reported.

To comprehensively map the extent and plasticity of YAP1 phosphorylation and its role in shaping the interactome of YAP1, we performed affinity purification and mass spectrometry (AP‐MS) to quantify YAP1 interactors and phosphorylation sites in response to phosphatase inhibition (Fig 2A; Appendix Fig S2A–H; Dataset EV1). Specifically, we used YAP1, tagged with Strep‐HA (SH), ectopically expressed in HEK293 cells under the control of a doxycycline‐inducible promoter. We performed triplicate measurements at two time points (2 and 20 min) after treatment of cells with the tyrosine phosphatase inhibitor vanadate, and at two time points (60 and 150 min) after treatment with okadaic acid, a serine/threonine phosphatase inhibitor (Couzens *et al*, 2013). After stringent data filtering using a SAINT probability (Teo *et al*, 2014) > 0.90 for interactors assignment and a PTM localization score > 0.8 for phosphosites (see Materials and Methods for details), we mapped 25 YAP1 phosphorylation sites (Fig 2B, left) and detected 32 high confidence interacting proteins (Fig 2B, right). Remarkably, 96% of the claimed phosphosites and 84% of the identified interactors are supported by published evidence and corroborates the precision and reliability of the presented information.

Okadaic acid and vanadate differentially affect the magnitude of YAP1 phosphorylation and, to a lesser extent, interactor association (Fig 2C). Cumulative density function shows that okadaic acid had an impact on a larger number of phosphosites and caused a dramatic alteration, up to 100‐fold, of several PPIs, while vanadate affects a lower number of phosphosites and protein interactions (Fig 2C). As expected, the use of phosphatase inhibitors improved phosphosite detection: Only 15 YAP1 phosphosites were detected without treatment, whereas 25 phosphosites were detected cumulatively after the addition of the respective phosphatase inhibitors (Appendix Fig S2F). Identified YAP1 phosphosites are primarily localized in the N‐terminal TEAD interaction domain (aa 47–153, TID) or at the C‐terminus (aa 335–500) of YAP1, but not in the two WW domains (Fig 2D). The former region is characterized by phosphosites that have a higher functional relevance score (Ochoa *et al*, 2020; a predictive tool which estimates the impact of phospho‐sites) than the latter. Moreover, most sites, regardless of the sequence location, show very high functional scores (top 10% percentile or higher) against the entire human phosphoproteome (Ochoa *et al*, 2020; Fig 2D, upper panel).

**Figure EV1:**
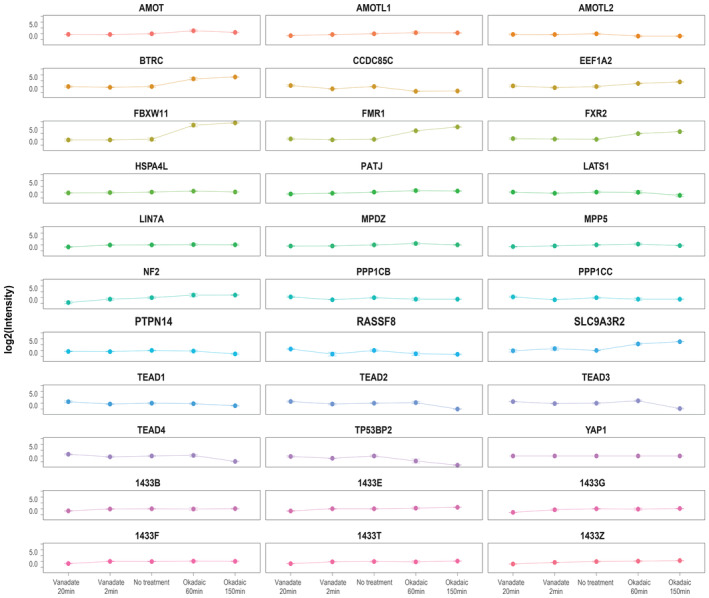
Plasticity of YAP1 interactors Kinetic profiles of Strep‐HA YAP1 interactors identified with SAINT SP score > 0.9 in at least one condition. Mean values of MS1 intensity from three independent replicates and SE error are reported for the following conditions (No treatment, Vanadate treatment for 2 and 20 min and Okadaic acid treatment for 60 and 150 min).

Next, we studied whether the temporal response to vanadate and okadaic acid would reveal distinct changes in YAP1 interactors. To do that, we used a fuzzy clustering approach to group protein response profiles, considering both treatments (see Materials and Methods). Rewiring of the YAP1 interactome under these perturbation conditions indicated three main clusters of alteration (Figs 2E and EV1). These clusters display distinct association dynamics and suggest that proteins exhibiting similar behavior may be part of the same complexes. Indeed, several structurally or functionally related proteins clustered together under the conditions tested, indicating that their interaction with YAP1 is modulated coordinately and controlled by YAP1 phosphorylation status. The first cluster contains the F‐box proteins BTRC and FBXW11, which interact more strongly with YAP1 after okadaic acid treatment compared with untreated cells. BTRC and FBXW11 are known to mediate SCF‐dependent ubiquitination and degradation of YAP1, after phosphorylation of S397, S400 (not detected) and S403 (not detected; Zhao *et al*, 2010). The second cluster consists of apicobasal polarity proteins (AMOT, INADL, MPDZ, MPP5, NF2) and 14‐3‐3 proteins. These proteins interacted less strongly with YAP1 in the presence of vanadate than untreated cells. The third cluster consists of proteins that showed reduced binding to YAP1 in the presence of okadaic acid compared with untreated cells. It includes the members of the ASPP/PP1A complex (CCDC85C, TP53BP2, RASFF8, PPP1CB, and PPP1CC) and TEAD protein family members. Consistent with previous reports, we found an inverse YAP1 association behavior of TEAD compared to 14‐3‐3 proteins, whereby, after okadaic acid treatment, the hyperphosphorylation of YAP1 in the TEAD interaction domain (TID; S127) resulted in a strongly reduced binding of YAP1 for TEAD proteins (TEAD1,2,3,4). By contrast, reduced phosphorylation of YAP1 S127 correlated with a strong decrease in binding of 14–3‐3 proteins (1433F, 1433B, 1433T, 1433E, 1433Z; Fig 2F). This is consistent with the previous finding that pS127 acts as a docking site for 14‐3‐3 proteins (Basu *et al*, 2003) and causes YAP1 translocation. Taken together, these data provide an extensive, quantitative map of YAP1 phosphosites/interactors and their responsiveness upon phosphatase inhibition, which serves as a reference library for subsequent experiments. Furthermore, it indicates how changes in the phosphorylation state of YAP1 are correlated with an organized reshaping of its interactome around three functionally coherent clusters of proteins with distinct association dynamics.

**Figure 3:**
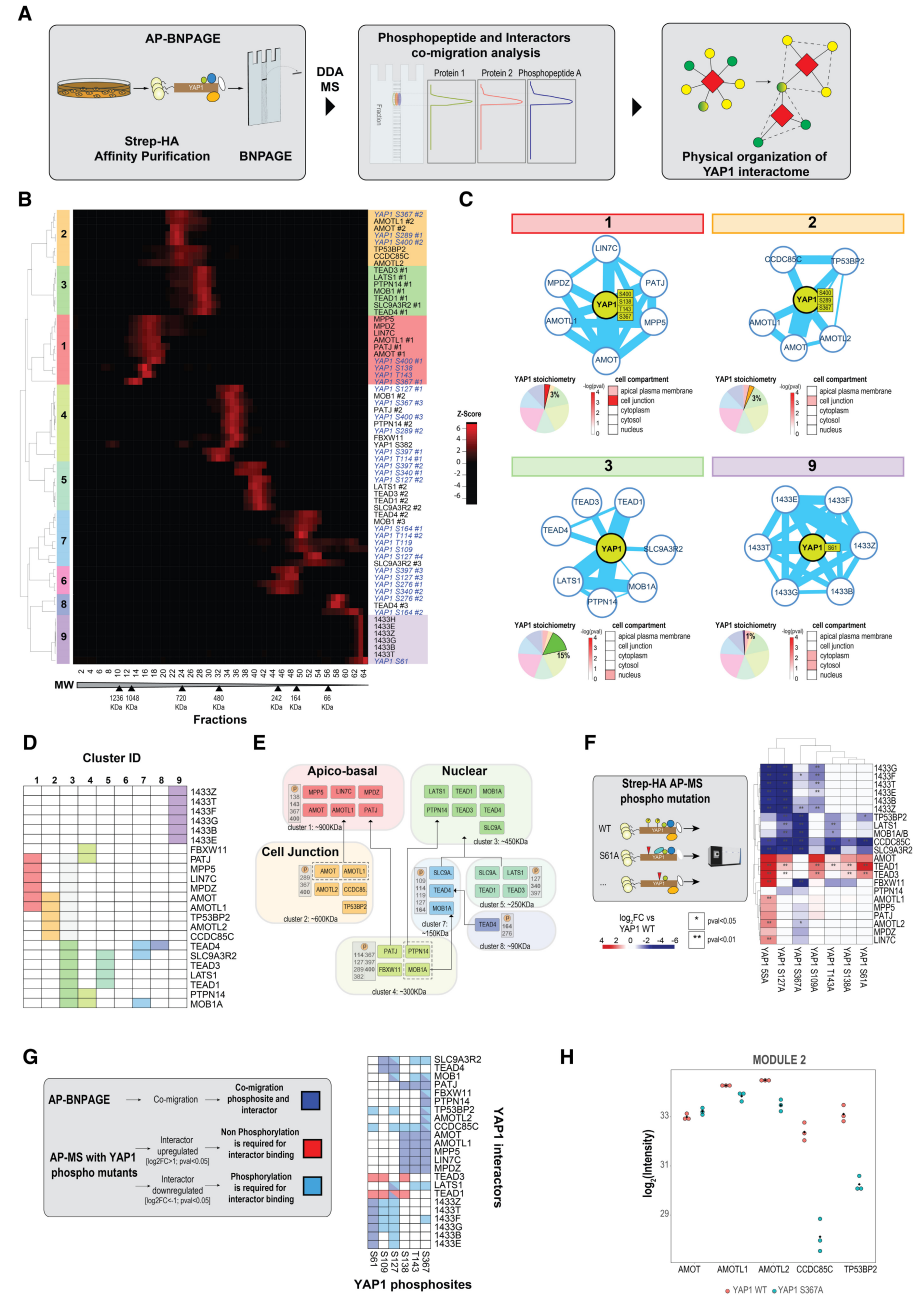
Deconvolution of native YAP1 complexes by protein correlation profiling Workflow of AP‐BNPAGE to investigate the organization of YAP1 interactors and phosphosites. After Strep purification and native elution with biotin, YAP1 interactors and YAP1 phosphopeptides are fractionated based on their electrophoretic mobility under native conditions. Quantitative proteomics data is obtained from the integration of MS1 peptide signal over 64 gel fractions to generate migration profiles of proteins and phosphosites. Co‐migration profiles of YAP1 interactors and phosphosites reveals the presence of modules which describe the physical organization of the YAP1 interactome.Unsupervised hierarchical clustering of YAP1 interactors and phosphosites intensity profiles. MS1 intensity of YAP1 interactors and phosphosites are scaled from 0 to 1. Migration peaks with a minimal intensity of 0.4 and with a FWMH = 2 are identified, smoothed and split. Peak distance correlation provides the identification of a module (z‐score in the heatmap). Raw data and the effect of analysis steps on the migration profiles are shown in Appendix Figs S3 and S4 for YAP1 interactors and phosphosites respectively.Composition, YAP1 phospho‐signature, stoichiometry (pie chart) and localization (heatmap) of four selected modules (1,2,3,9). Edge thickness corresponds to the number of physical PPIs annotated in BioGRID database.Composition of the nine identified modules (group of co‐migrating proteins).Graphical representation of modules identified by AP‐BNPAGE experiment. Each module is characterized by PTM signature and the estimated molecular weight from AP‐BNPAGE experiment. Relationship between modules (fragment or assembly) are indicated by arrows.Effect of phospho mutations on YAP1 interactors (identified previously in the AP‐BNPAGE experiment). Wild‐type Strep‐HA YAP1 and phosphosite mutants (six single site and one multiple site mutations, 5SA) are purified with streptavidin beads and interactors are quantified by MS using MS1 signal intensity. Values reported in the heatmap show the average (three independent replicates) intensity of interactors as log2 fold change compared with the control (wild type YAP1). Upregulated and downregulated YAP1 interactors are shown in red and blue respectively; significant changes (two‐sided unpaired Student's *t*‐test) are marked with asterisks.Association data between YAP1 phosphosites and interactors from AP‐BNPAGE and mutants AP‐MS experiments. Purple tiles show phosphosite and interactor associations generated by co‐migration profiles, cyan and red tiles show the average fold change of interactor downregulation (log2FC < −1, *P* < 0.05) and upregulation (log2FC > 1, *P* < 0.05) in the corresponding wild‐type condition.MS1 intensity of YAP1 interactors from module two after AP‐MS of YAP1 WT and phosphomutant (S367A; respectively, red and cyan).

### Deconvolution of native YAP1 complexes by protein correlation profiling

**Figure EV2:**
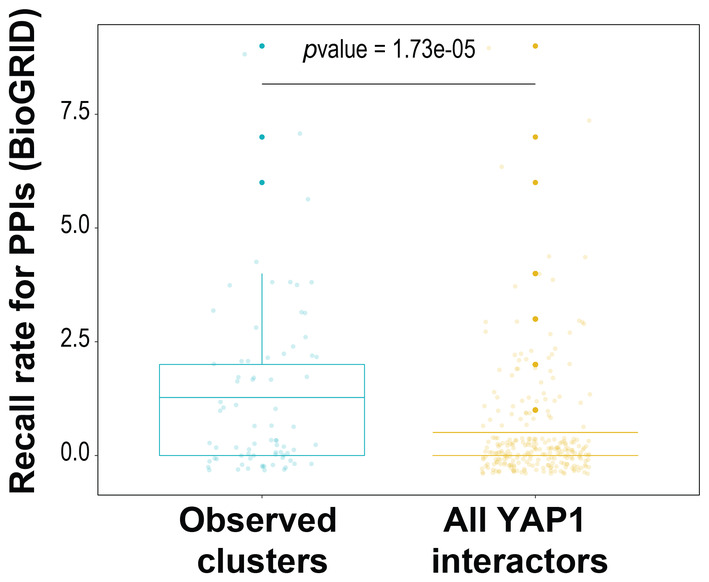
Recall rate for Protein–Protein Interacion pairs in co‐migrating clusters is higher than all pairs of YAP1 interactors Recall rate (BioGRID) for Protein–Protein Interaction pairs in co‐migrating cluster compared to all interactions pairs of YAP1 interactors. Significance is evaluated by two‐side unpaired *t*‐test assuming normal distribution. The boundaries of the box plot correspond to the quantiles Q1 (25%) and Q3 (75%). Lower and upper whiskers are defined by Q1− 1.5IQR and Q3+ 1.5IQR.

Since the AP‐MS data result from concurrently purified YAP1 complexes, AP‐MS data do not inform about the presence of differentially phosphorylated YAP1 complexes in the same sample (Bludau & Aebersold, 2020). To assign the identified YAP1 interactors to specific YAP1 subcomplexes, we subjected a Strep affinity purified YAP1 complex mixture to electrophoretic native size fractionation (BNPAGE; Bode *et al*, 2016; Fig 3A). Specifically, we separated YAP1 complexes along the axis of native electrophoretic separation (molecular weight), excised 64 consecutive gel slices and measure with an untargeted MS1‐based approach the intensity of YAP1 phosphopeptides and interactors (identified in the experiment above, Appendix Fig S2H) to generate migration profiles of protein and YAP1phosphopeptides (Fig 3B; Appendix Figs S3, S4, and S5A–H; Dataset EV2). Migration profiles of the same entity (protein, phosphopeptides) showing multiple peaks (i.e., potentially being present in multiple assemblies) were normalized to the apex, split, and smoothed based on the detection of local maxima (peaks); the resulting single peaks from different proteins/phosphopeptides were grouped by unsupervised hierarchical clustering into co‐migrating modules (see Materials and Methods and the reference; Fossati *et al*, 2021). Critically, the YAP1 profile across the analyzed fractions indicates the existence of electrophoretically well‐resolved peaks of varying abundance and MW (Appendix Figs S3, S4, and S5G for the visualization of raw and smoothed profiles). We observed that, YAP1 phosphosites and interactors exhibit similarly discrete partitioning across the fractionation dimension (Fig 3B and C; Appendix Figs S3 and S4). Of note, the AP‐BNPAGE protocol does not affect the original overall abundance range and stoichiometries of YAP1 interactors, as the sum of intensities per fraction are highly correlated with the unseparated YAP1 interactome (Appendix Fig S5D). Migration profile analysis indicated separation of nine YAP1 modules with specific YAP1 phosphorylation patterns (phosphosignature; Fig 3B). Several lines of evidence support the notion that these modules are indeed biologically relevant entities and not the result of coincidental co‐migration. (i) Interactions between pairs of proteins belonging to the identified modules have been reported in PPIs database (Oughtred *et al*, 2019) significantly more often than all possible pairs of YAP1 interactors. (Fig EV2) (ii) five of seven complexes (excluding modules containing exclusively phosphopeptides or just a single protein) can be assigned to a specific cellular compartments GO term (at least one entity with *P‐*value from hypergeometric test smaller than 0.05; Appendix Fig S5F); (iii) complex components are highly connected and more likely to be part of known, stable complexes (e.g., ASPP‐PP1, RICH1‐AMOT, Fig 3C; Tsitsiridis *et al*, 2023) or to contain homologous proteins (e.g., TEAD, 14‐3‐3; Fig 3C).

We next examined the distribution of the YAP1 interactors identified above across the nine identified modules (Fig 3D). We found three high molecular weight modules (modules 1–3), several partially overlapping modules of intermediate size (modules 4–8) and smaller molecular weight YAP1 complexes containing 14‐3‐3 proteins (module 9). The first module contains mostly apical–basal proteins, including PATJ, MPP5, LIN7C, MPDZ, AMOT, and AMOTL1 of the RICH1/AMOT polarity complex (Wells *et al*, 2006, 1). The second module encompassed tight junction proteins, including members of the ASPP/PP1 complex (Bertran *et al*, 2019) involved in YAP1 S127 dephosphorylation (Royer *et al*, 2014), in addition to AMOT, AMOTL1, and AMOTL2. The third module primarily consisted of known nuclear interactors of YAP1, including the TEAD transcription factors TEAD1, TEAD3, TEAD4, LATS1, and its activator MOB1 as well as PTPN14. Modules 5, 7, and 8 are most likely fragments or assembly intermediates of this nuclear module, while we interpret module 4 as a convolution of a fragment of the nuclear module and two additional proteins (Fig 3E). Overall, our profile analysis separates YAP1 interactome in distinct complexes that are linked to its signal integration and effector function.

In most modules, we were able to identify specific YAP1 phospho‐signatures (ensemble of phosphosites; Fig 3C). For example, in the apical cell polarity complex (module 1), YAP1 was phosphorylated on S138, S143, S367, and S400. Among these, S138 and S367 are phosphorylated by CDK1 through a mechanism involving the interaction with the polarity protein PATJ (Bui *et al*, 2016). This is in striking contrast to the fragments or assembly intermediates of the nuclear module (modules 4, 5, 7, 8, Fig 3E) where YAP1 is richly phosphorylated on several sites and the larger nuclear module itself (module 3), where no YAP1 phosphorylation sites were detected. This pattern cannot be explained by the overall abundance of YAP1 in these different complexes, since YAP1 intensity is comparable in the nuclear module and in some of the submodules (Appendix Fig S5G). These results suggest that S127 may not be the sole regulator of nuclear/cytoplasmic transport, but that it may require dephosphorylation of multiple sites.

To further validate potential roles of YAP1 phosphosites in the regulation of subcomplexes, we generated cell lines expressing Strep‐HA phosphosite mutants of YAP1 (six single‐site selected from the AP‐BNPAGE experiment and one multiple‐site mutations, 5SA; Fig 3F; Appendix Fig S6A–F; Dataset EV3). We next subjected YAP1 phospho‐null mutants to AP‐MS and compared the resulting interactions with the YAP1 WT interactome (Fig 3F). Each mutation exhibits a distinct and overlapping signature of YAP1 interactors. As expected, mutation in S127 (and to a similar extent also S109) strongly reduced 14‐3‐3 binding while it increased binding to TEAD. By contrast, other N‐terminal phosphosite mutants such as S61A and S138A did not affect the binding of 14‐3‐3 proteins, but the levels of TEAD1/2 in YAP1 complexes.

When we analyzed the constitutively active YAP1 mutant 5SA (S61A, S109A, S127A, S164A, S381A), we observed a strong remodeling of YAP1 interactors, which, in addition to the reduction of 14‐3‐3 and a strong increase in TEAD binding, showed a unique increase of AMTOL1/2, LIN7C, and FBXW11. Mutation of the poorly studied S367 phosphosite, on the contrary, did not affect 14‐3‐3 nor TEAD binding, but resulted in a characteristic PPIs pattern not observed in the other mutants.

With the aim to identify which phosposites identified in a given module are functionally linked to the integrity of this module, we integrated AP‐BNPAGE co‐migration data (Fig 3B) with AP‐MS data using YAP1 phospho‐mutants (Fig 3F). In the resulting matrix (Fig 3G), we define association as co‐migration of a protein (y axis) with a YAP1 phosphosite (*x* axis) in a given module. Among all 37 co‐migration associations (dark blue tiles), we found nine pairs where phosphorylation of the indicated YAP1 phospho‐site regulate module integrity (tiles dark blue/cyan or tiles dark blue/red). Remarkably, in eight of these nine associations, phosphorylation is needed for the binding indicating a functional role for the corresponding site (dark blue/cyan tiles). For example, we found that S367A strongly decreased all proteins of module 4 (FBXW11, MOB1A, MOB1B, PATJ, and PTPN14; Appendix Fig S5I) as well as two proteins of module two (CCDC85C and TP53BP2; Fig 3H). Notably, these two proteins exclusively co‐migrate in a single module (Fig 3B).

Overall, our strategy shows that integrated MS‐based analysis of complex composition and phosphorylation state, combined with native fractionation of purified complexes, can deconvolute the interactome (sum of all binary interactions) in biologically meaningful YAP1 complexes and assign complex‐specific YAP1 proteoforms.

### Identification of YAP1 phosphorylation and interactors recapitulate known and suggest new control mechanisms

**Figure 4:**
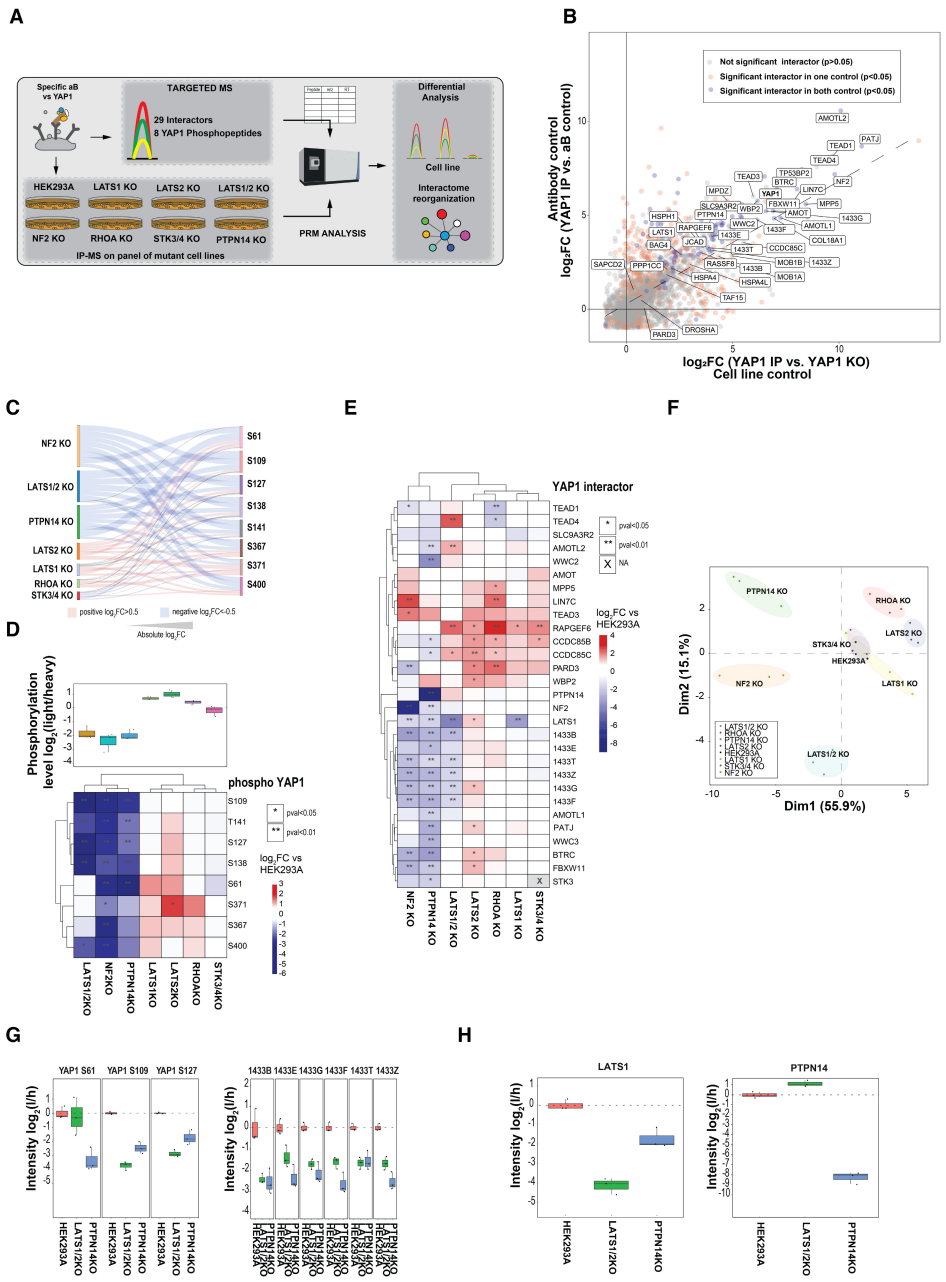
Identification of YAP1 phosphorylation and interactors recapitulate known and suggest new control mechanisms Experimental workflow for profiling endogenous YAP1 phosphorylation and interaction changes in cells lacking known Hippo pathway members. After YAP1 endogenous immuno‐affinity purification from the indicated mutant HEK293A cells, phosphopeptides and interactors were quantified by targeted proteomics (IP‐MS). The experiment has been performed with anti‐C‐terminal peptide antibodies and with two controls: (i) antibody control, IP‐MS experiment using non‐specific antibodies and (ii) cell line control, IP‐MS experiment using YAP1 specific antibodies in YAP1 KO cell line.Correlation plot of proteins enriched in the YAP1 endogenous immune‐affinity purification with two different controls cell line control and aB control. Cell line control is performed with YAP1 IP‐MS from YAP1 KO cells and antibody control with non‐specific control antibodies. The enrichment for cell line and antibody control is calculated as the log2 fold change ratio from average MS1 protein intensity of three independent replicates compared with the controls. Proteins identified and filtered as interactors (SP > 0.9) in fractionated AP‐MS from HEK293A cells expressing epitope tagged YAP1 are annotated. Protein significantly enriched in both controls are marked in blue, in orange those significantly enriched with only one control, in gray those not significantly enriched The significance is calculated with two‐sided unpaired Student's *t*‐test.Sankey plots shows the effect of protein deletion on YAP1 phospholandscape. Color code indicates an increase compared with wild‐type (log2 > 0.5, red) or decrease (log2 < −0.5, blue). Size of the lines is proportional to the log2 fold changes.Unsupervised hierarchical cluster of YAP1 phosphopeptides in a panel of seven cell lines with genetic deletions of indicated Hippo signaling genes. Values reported in the heatmap represent the log2 fold change of phosphopeptide intensity average from three independent biological replicates compared with parental cell (HEK293A). Upregulated and downregulated phosphopeptides are shown in red and blue, respectively; significant changes are marked with asterisks (two‐sided unpaired Student's *t*‐test). On the top, boxplot shows the average phosphorylation level of 8 monitored YAP1 phosphopeptides per condition (*n* = 3 biological replicates). The boundaries of the box plot correspond to the quantiles Q1 (25%) and Q3 (75%). Lower and upper whiskers are defined by Q1− 1.5IQR and Q3+ 1.5IQR.Unsupervised hierarchical cluster of YAP1 interactors in a panel of seven cell lines with Hippo genetic deletions. Values reported in the heatmap represent the log2 fold change of YAP1 interactor intensity average from three independent biological replicates compared to parental cell (HEK293A). Upregulated and downregulated phosphopeptides are shown in red and blue, respectively; significant changes are marked with asterisks (two‐sided unpaired Student's *t*‐test).Principal component analysis based on both phosphorylation and interaction data (target proteins of the KO are removed from the data). Various mutants are highlighted in different colors and every dot represents a replicate condition.Intensities, expressed as log2 ratio of light (endogenous peptides) and heavy (reference peptides) of identified YAP1 phosphopeptides with LATS1 sequence motif (S61, S109, S127; left) and 14‐3‐3 protein family in the indicates cell lines (*n* = 3 biological replicates). The boundaries of the box plot correspond to the quantiles Q1 (25%) and Q3 (75%). Lower and upper whiskers are defined by Q1− 1.5IQR and Q3+ 1.5IQR.Intensities, expressed as log2 ratio of light (endogenous peptides) and heavy (reference peptides) of PTPN14 and LATS1 after YAP1 immuno‐affinity purification in the indicated cell lines (*n* = 3 biological replicates). The boundaries of the box plot correspond to the quantiles Q1 (25%) and Q3 (75%). Lower and upper whiskers are defined by Q1− 1.5IQR and Q3+ 1.5IQR.

We have thus far mapped the effects of phosphorylation changes in the interactome of total YAP1 and further resolved the YAP1 interactome in co‐migrating protein groups (complexes), of which each was associated with a specific YAP1 phospho‐signature/proteoforms. To relate the observed YAP1 proteoforms to the regulation of the Hippo pathway, we analyzed YAP1 phosphorylation and complex formation in a panel of HEK293A knockout (KO) cell lines where each cell line lacked a key regulator of the Hippo pathway (Fig 4A; Plouffe *et al*, 2016). We first established a protocol to affinity‐purify endogenous YAP1 with custom‐generated anti‐YAP1 antibodies (IP‐MS). Compared with the inducible ectopic expression of YAP1 used in the previous experiments (Fig 2), this approach is more compatible with systematic YAP1 interactor analysis in HEK293A mutants lacking critical Hippo signaling components and more reliably reflects endogenous stoichiometries. To maximize the specificity of our purification, we used a double control strategy by using nonspecific antibodies (aB control) and a YAP1 KO HEK293A line (cell line control; see Materials and Methods for details; Fig 4B; Appendix S7A–D, and F; Dataset EV4). Using this approach, we could recover almost all interactors previously defined with AP‐MS of fractionated ectopically expressed YAP1 with high specificity and sensitivity (AUC 0.88 and 0.85; Appendix Fig S7E and G). A global description of all YAP1 interactors and phosphosites identified in all experiments of this work is reported (Appendix Fig S8A and B, respectively for interactors and phosphosites).

**Figure EV3:**
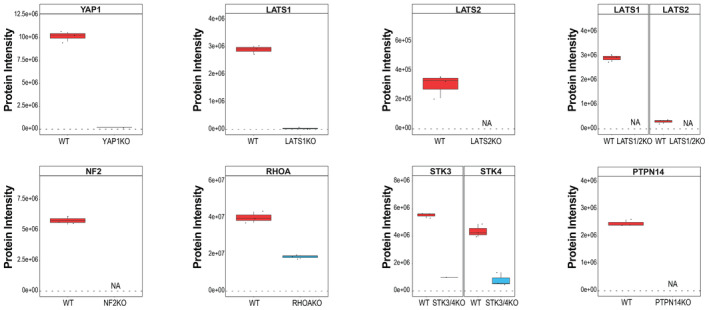
Validation of KO cell lines Targeted proteomic quantification and characterization of genetic deletion in nine different cell lines. The boxplot reports the intensities of the indicated proteins in the parental HEK293A control cell line (left, red) and the respective KO cell line (right, blue). Data shown in the plot are generated from three independent biological replicates. The boundaries of the box plot correspond to the quantiles Q1 (25%) and Q3 (75%).

Next, we used targeted mass spectrometry to analyze a panel of KO cell lines lacking key component of the Hippo pathway (Plouffe *et al*, 2016), including the kinases LATS1, LATS2, LATS1/2, STK3/STK4, the GTPase RHOA, NF2, the phosphatase PTPN14 and YAP1 itself as a control. Gene deletion were confirmed by the absence of the respective proteins as measured by targeted proteomics and Western blot (only PTPN14KO, Appendix Fig S8F), except for STK3/4 KO and RHOA KO cells that showed around 15 and 45% of residual STK4 and RHOA levels compared with parental controls, respectively (Fig EV3; Appendix Fig S8C–E; Dataset EV5). Protein expression of other Hippo pathway regulators was only mildly affected by the gene deletions (Appendix Fig S8E), suggesting that disruption of the Hippo network did not significantly alter protein expression or stability.

Finally, we performed endogenous YAP1 purifications in triplicate for each cell line, followed by targeted PRM measurements using heavy labeled reference peptides. Overall, we quantified 29 interacting proteins and eight YAP1 phosphopeptides (Appendix Fig S9A–E; Dataset EV6 for a summary of all YAP1 peptides monitored in the targeted experiments). To interpret the acquired data, we performed unsupervised hierarchical clustering separately on phosphopeptide intensities (Fig 4C and D) and protein intensities (Fig 4E). YAP1 phosphopeptide values were normalized to YAP1 protein intensity to differentiate variations in protein abundance from changes in phosphorylation.

Sankey plot (Fig 4C) and the average phosphorylation levels (Fig 4D, upper panel) show that phosphorylation levels of YAP1 clearly separated a group of mutants that caused YAP1 strong hypophosphorylation consisting of NF2, LATS1/2, and PTPN14 KO cells from a group showing a mild increase in phosphorylation, consisting of the LATS1, LATS2, RHOA KO cells. By contrast, the STK3/4 double mutant cells only showed a very moderate effect on YAP1 phosphorylation on the tested sites (Plouffe *et al*, 2016; Fig 4C and D). Among the tested phosphopeptides, two distinct clusters with complementary behaviors were observed. The first cluster consisted of sites located N‐terminally, specifically S109; S127; S138; S143, consistently showing a highly significant dephosphorylation in the LATS1/2, NF2 and PTPN14 mutants, and no or mild upregulation in the LATS1, LATS2, and RHOA mutants. The second cluster consisted of sites located C‐terminally, specifically sites S371; S379; S400, showing weaker downregulation in the LATS1/2, NF2 and PTPN14 mutants and stronger upregulation in LATS1, LATS2, and RHOA mutants. Site S61 displayed a more complex modulation, as shown in Fig 4D. Although the peptide encompassing S61 contains a LATS consensus motif (Hao *et al*, 2008), phosphorylation of this site is not affected by the deletion of LATS1/2, implying a role for other kinases, as already suggested by *in vitro* studies (Basu *et al*, 2003; Hao *et al*, 2008; Wang *et al*, 2020).

Unsupervised hierarchical clustering of YAP1 interactor intensities closely mirrored the clustering of phosphopeptides. The LATS1/2, PTPTN14, and NF2 KO cells showed a systematically decreased interaction with cytoplasmic proteins (14‐3‐3 proteins). By contrast, the others mutants (LATS1, LATS2, RHOA, STK3/4) revealed an increased or unchanged association, as indicated by the stable profile of 14–3‐3 proteins (Fig 4E). In several respects, these data are in agreement with previous knowledge: our analysis confirms the positive role on YAP1 phosphorylation by NF2 and LATS1/2 already observed by others (Zhao *et al*, 2007, 2008; Ma *et al*, 2019), but adds a quantitative phosphosite‐level resolution absent in previous analyses (Plouffe *et al*, 2016). As reported previously, deletion of RHOA (only partial) mediates the mechanical stress‐induced activation of YAP1, induces YAP1 hyper‐phosphorylation, and reduces its nuclear localization (Dupont *et al*, 2011; Plouffe *et al*, 2016; Fig 4C–E). Finally, LATS2 KO cells and, to a lesser extent, LATS1 KO cells resulted in mild, but widespread hyperphosphorylation of YAP1 sites, in contrast to the strong downregulation driven by the double mutant. These results confirm that the two kinases are redundant, corroborating the characterization of the KO from prior phospho‐tag experiments (Plouffe *et al*, 2016). We surmised that the upregulation of the phosphopeptides in the single KO mutants could be due to a compensatory mechanism, whereby the loss of one kinase leads to increased expression of the other. Analysis of the levels of LATS2 in LATS1 KO lysates supports this hypothesis (Appendix Fig S8G).

Remarkably, we observed that YAP1 phosphorylation and complex formation patterns in the absence of the phosphatase PTPN14 closely resembles those in NF2 and LATS1/2 KO cells. This observation was further confirmed by a principal component analysis on the combined interactome and phosphoproteome data (Fig 4F), showing a pronounced separation of these three mutants along the major component as compared to the other mutants tested (1^st^ dimension: 55.9% of explained variance). Because YAP1 phosphorylation pattern and complex formation in PTPN14 KO cells resemble those found in cells lacking NF2, which is an upstream activator of LATS1/2, as well as in LATS1/2 double mutant cells, we hypothesized that PTPN14 may play an analogous role in activating YAP1 phosphorylation by LATS1/2. This is supported by several lines of evidence: (i) two of the LATS1/2 sites on YAP1, S109, S127^28^, are negatively regulated in PTPTN14 KO cells and, as a consequence, the interaction with 14‐3‐3 proteins is decreased because the docking site is eliminated (Fig 4G); (ii) PTPN14 KO reduces the binding between YAP1 and LATS1, but LATS1/2 KO does not decrease the amount of PTNP14 associated with YAP1 (Fig 4H, left and right, respectively); (iii) the interaction between PTPN14 and LATS1 has already been reported (Poernbacher *et al*, 2012; Couzens *et al*, 2013; Wilson *et al*, 2014; Go *et al*, 2021); and (iv) evidence for a role of PTPN14 in the modulation of YAP1 phosphorylation and activity have been provided (Wang *et al*, 2012, 2020; Liu *et al*, 2013; Wilson *et al*, 2014).

Taken together, our targeted proteomics of endogenous YAP1 immuno‐purified from cells lacking Hippo pathway regulators resolved their roles in controlling YAP1 activity at the level of YAP1 phosphorylation and complex formation and suggests a key role for PTPN14 in controlling LATS‐dependent YAP1 regulation.

### Reduced LATS1/2‐YAP1 complex formation, enhanced nuclear translocation and activation of YAP1 in PTPN14 mutant cells

**Figure 5:**
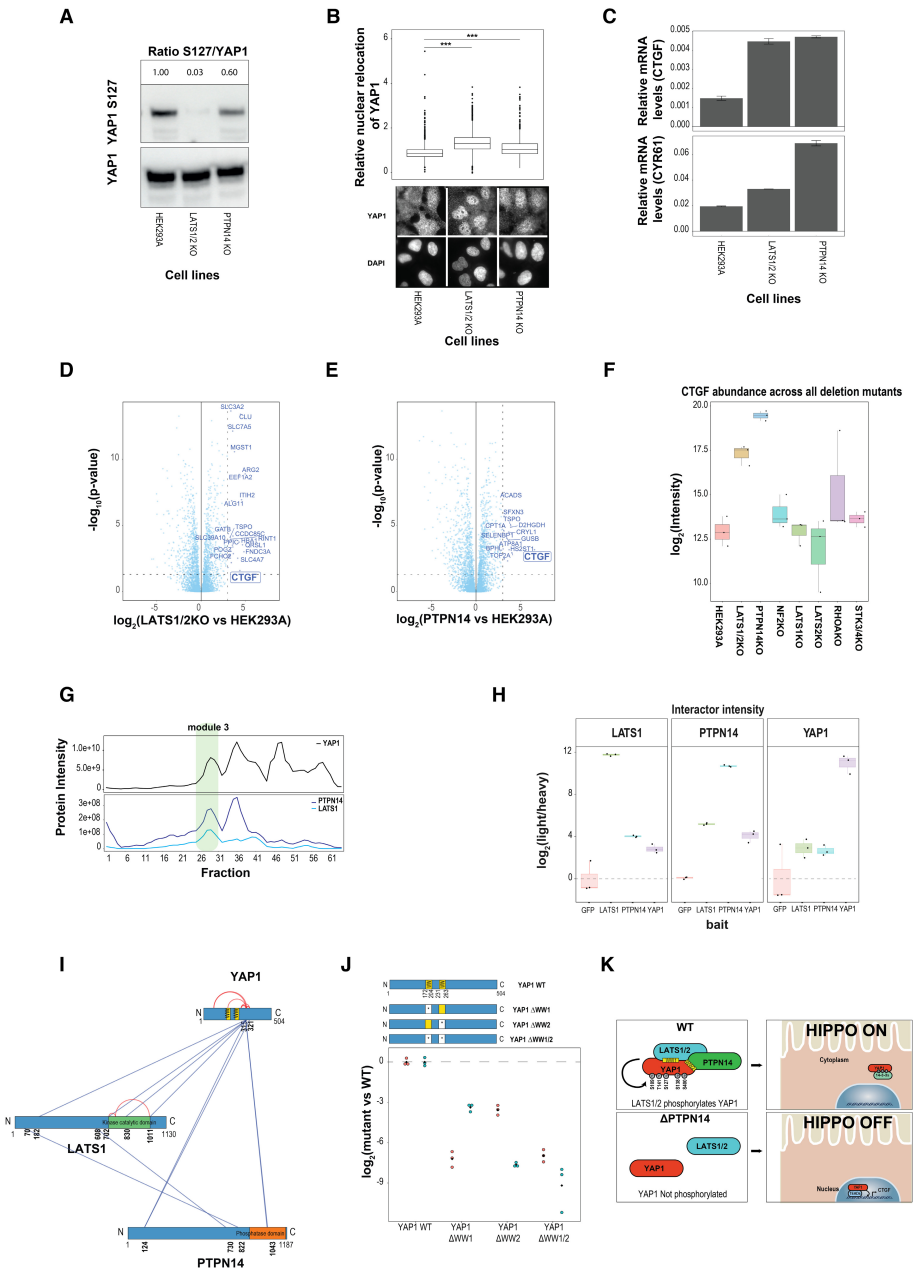
Reduced LATS1/2‐YAP1 complex formation, enhanced nuclear translocation and activation of YAP1 in PTPN14 deleted cells AImmunoblot with anti YAP1 and anti phospho‐YAP1(S127) antibodies on protein lysates from indicated WT and indicated KO cell lines (LATS1/2 and PTPN14). Quantitative values reported above are normalized for the abundance of YAP1.BSubcellular localization of YAP1 in LATS1/2 KO and PTPN14 KO cells. *Lower panel*: the localization of YAP1 is probed using immunofluorescence and visualized using wide‐field microscopy. The DAPI‐signal is used to compare the nuclear relocation of YAP1 among the cell lines. *Upper panel*: quantification of relative nuclear relocation of YAP1 combined from three independent experiments. The boundaries of the box plot correspond to the quantiles Q1 (25%) and Q3 (75%). Lower and upper whiskers are defined by Q1− 1.5IQR and Q3+ 1.5IQR. The significance is indicated with ***P < 0.001 (two‐sided unpaired Student's *t*‐test). Total number of cells analyzed for HEK 293A WT, LATS1/2 KO, and PTPN14 KO cells are 1514, 1673, and 1320 respectively.CCTGF and CYR61 (YAP1 target genes) transcript levels (qPCR) for HEK293A WT, LATS1/2 KO, and PTPN14 KO. Data from three independent biological replicates are presented as mean values±SD.D–FDifferential protein expression data. Volcano plots displaying the log2 fold changes of protein intensity and the corresponding significance (two‐sided unpaired Student's *t*‐test, *N* = 3 biological replicates) of LATS1/2 KO (D) and PTPN14 KO (E) compared to WT control HEK293A. Proteins with log2 FC > 3 and *P*‐value < 0.05 are highlighted with their gene names. (F) Boxplot showing CTGF protein intensity level (log2) across the examined mutant cells. The boundaries of the box plot correspond to the quantiles Q1 (25%) and Q3 (75%). Lower and upper whiskers are defined by Q1− 1.5IQR and Q3+ 1.5IQR.GCo‐migration profile of PTPN14, LATS1 and YAP1 after YAP1 AP‐BNPAGE complex fractionation (data extrapolated from Dataset EV2).HReciprocal enrichment of the PTPN14‐LATS1‐YAP1 complex in the Strep AP‐MS of each of the complex members. Boxplot showing the intensity of interactors (expressed as log2 ratio between endogenous peptide, light, and reference peptide, heavy) across different baits (GFP, LATS1, PTPN14, and YAP1). The boundaries of the box plot correspond to the quantiles Q1 (25%) and Q3 (75%). Lower and upper whiskers are defined by Q1− 1.5IQR and Q3+ 1.5IQR. The data were obtained from three independent replicates.IMap of cross‐linked peptides identified by AP‐XL‐MS of Strep‐HA‐tagged YAP1. Purified YAP1 is subjected to chemical crosslinking with a lysine reactive homo‐bifunctional molecule (DSS). Identified cross‐linked peptides enables to map lysine residues which are in close proximity (< 30 Å). Inter‐ and intraproteins cross‐linked peptides are annotated in blue and red, respectively.JLATS1 and PTPN14 levels in YAP1 WT and in indicated WW domain mutants. Protein intensities are measured by AP‐MS. The plot reports log2 intensity relative to YAP1 wild‐type for each of the three biological replicates.KModel on the YAP1 inhibition by PTPN14: PTPN14 promoting the interaction between LATS1 and YAP1, increase phosphorylation level of YAP1 (S109, S141, S127, S138, S400) which, in turn, leads to YAP1 inactivation and cytoplasmic retention.

Finally, we aimed to gain insights into the mechanism how PTPN14 could act as a positive regulator of LATS1/2 kinases for the control of YAP1. We first validated the effect of PTPN14 deletion on LATS1 activity by monitoring levels of phosphorylation of the known LATS1/2 substrate, site S127, on YAP1 by Western blot. We found that PTPN14 KO reduced the level of YAP1 S127 phosphorylation to around 60% compared with the WT condition, confirming the MS results obtained with purified endogenous YAP1 (Figs 4D and 5A). Next, we compared YAP1 subcellular localization by immunofluorescence in LATS1/2 and PTPN14 KO cells. In keeping with our previous results and previously published data (Plouffe *et al*, 2016; Wang *et al*, 2020), we found that YAP1 was localized in the cytoplasm in WT HEK293A cells, while the nuclear fraction increased upon removal of LATS1/2 and, to a lesser but still significant extent, upon PTPN14 deletion (Fig 5B). Finally, we compared the mRNA expression of CTGF and CYR61, two established YAP1 target genes in the two mutant cell lines. We found an approximately threefold increase in the levels of CTGF mRNA in both LATS1/2 and PTNPN14 mutants compared with parental HEK293A cells. Lack of PTPN14 was also associated with an increase of CYR61, which was even stronger than in LATS1/2 mutants (Fig 5C).

We next validated the finding at the proteome level by performing proteome profiling across the KO cell lines using data‐independent MS acquisition (DIA). We identified 4,436 proteins (Fig 5D–F; Appendix Fig S10A–D; Dataset EV7), and carried out differential expression analysis to identify proteins showing differential abundance under either LATS1/2 or PTPN14 KO (see Materials and Methods). Consistent with the above results, we confirmed increased CTGF protein levels in only LATS1/2 mutant cells and even greater CTGF expression in PTNPN14 KO cells (Fig 5F).

These orthogonal lines of evidence strongly support an involvement of PTPTN14 in the regulation of LATS1/2 and YAP1 activity but do not provide clear indications about the underlying mechanism. Because our interaction data indicate that LATS1/2 are not required for the YAP1‐PTPN14 interaction, but that the reciprocal is true (Fig 4H, left and right, respectively), we propose the existence of a trimeric complex where PTPN14 mediates the interaction between YAP1 and LATS1/2 kinases. This putative complex would reminisce the characterized trimeric complex LATS‐PTPN14‐KIBRA (Wilson *et al*, 2014), in that YAP1 and KIBRA shares two WW domains with a good alignment score (BLASTp analysis, *P* = 5e−16) and it is reported that YAP1 associates with PTPN14 and LATS in a WW/PPxY‐dependent manner (Huang *et al*, 2013; Liu *et al*, 2013; Michaloglou *et al*, 2013; Wang *et al*, 2020). The AP‐BNPAGE data support the hypothesis for the presence of YAP1‐LATS1‐PTPN14 complex and indicate co‐migration of the three proteins in a module (Figs 3C, module 3 and 5G). To further corroborate this finding, we carried out quantitative reciprocal AP‐MS in HEK293 cells expressing Strep‐HA tagged YAP1, LATS1, PTPN14, and GFP as control under doxycycline‐inducible promoter (Fig 5H; Appendix Fig S11A–C; Dataset EV8). The analysis of the resulting AP‐MS data confirm that each of the three purifications enriches the other two complex members compared with the control. This in turn confirms that interactions between the three proteins are not mutually exclusive, which is also in agreement with published binary interaction data obtained by co‐immunoprecipitation and by proximity labeling experiments (Couzens *et al*, 2013; Wilson *et al*, 2014; Oughtred *et al*, 2019; Go *et al*, 2021).

**Figure EV4:**
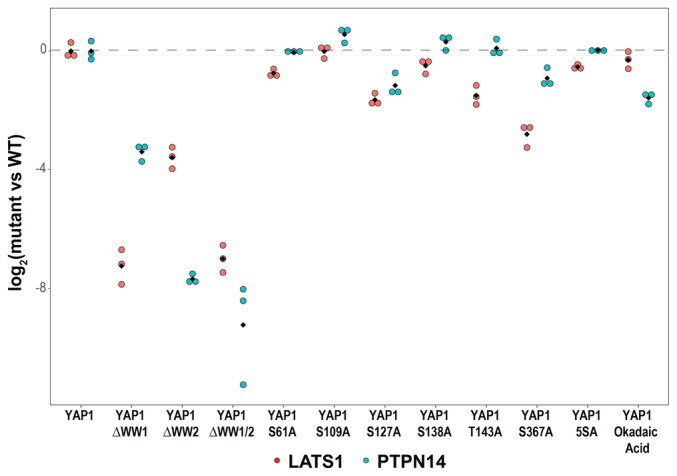
Effect of YAP1 mutants on YAP1 interactions with PTPN14 and LATS1 Intensity profile of YAP1 interactors in a panel of YAP1 mutants. The panel of mutants is composed by Strep‐HA YAP1 phospho mutants (six single mutants and one with multiple mutations S5A) and WW domain mutants (ΔWW1, ΔWW2, and ΔWW1/2). Interactors are identified and quantified by MS1 intensity after streptavidin affinity purification of YAP1. LATS1 (red) and PTPN14 (cyan) MS1 intensities of three independent biological replicates are expressed as log2 fold change compared with YAP1 wild‐type control.

To study the mechanism of ternary complex formation, we first asked whether the interaction between YAP1, LATS1, and PTPN14 is direct. Evidence for direct interactions between WW domain and PPXY motif (contained in YAP1‐LATS and YAP1‐PTPN14) has been previously provided by biochemical reconstitution (Schuchardt *et al*, 2014); however, no evidence for a direct LATS‐PTPN14 interactions exists yet. Therefore, we performed affinity purification of ectopically expressed Strep‐HA YAP1 followed by cross‐linking and mass spectrometry (AP‐XL‐MS). Cross‐linked peptides indicate the presence of lysine residues that are in close proximity (less than 30 Å) and can be used as molecular ruler to pinpoint protein regions in the interaction interface (Leitner *et al*, 2016; Sinz, 2018). We could identify four cross‐links between YAP1 and LATS1, three cross‐links between YAP1 and PTPN14, and two between PTPN14 and LATS1 supporting the idea that these interactions are direct (Fig 5I; Dataset EV9). Interestingly, all YAP1 cross‐linking sites are found in the protein central section, c‐terminus to the WW domains in position 315 and 321. Intraprotein cross‐linking peptides show that these residues are in close proximity with the lysine 280 (close to the second WW domain) and to the lysine 181 (in the first WW domain). As reported before, WW domains mediates the interactions by binding short linear PPxY motif of different YAP1 interactors, among all PTPN14 and LATS1 (Michaloglou *et al*, 2013; Schuchardt *et al*, 2014; Vargas *et al*, 2020), to test whether binding of these proteins via the two WW domains is not exclusive preventing the formation of a ternary complex, we performed Strep AP‐MS with YAP1 mutants in WW1, WW2, or both domains. We found that the WW1 mutation decreased LATS1 binding to a greater extent than WW2 mutation, while the opposite holds for the PTPN14 binding, confirming the known preference of PTPN14 for WW2 (Michaloglou *et al*, 2013; Fig 5J). None of the phospho‐site mutants had a similar effect, suggesting that the ternary complex is mainly regulated by WW domain (Fig EV4).

Overall, all our data support a model where PTPN14 binding to WW2 promotes LATS1/2 binding to the WW1 domain of YAP1. This allows LATS1/2‐mediated phosphorylation of YAP1 which, in turn, leads to YAP1 inactivation and cytoplasmic retention (Fig 5K).

## Discussion

Intra‐ and intercellular signaling systems largely depend on the modulation of the cellular proteome at different levels, including alteration of protein abundance, modification, and interactome remodeling. Most proteomic measurements of signaling systems to date have focused on the exhaustive analysis of single proteomic layers, exemplified by the analysis of altered protein abundance profiles (Eraslan *et al*, 2019), and/or the analysis of altered phosphorylation patterns (Frejno *et al*, 2020). Yet, it is well‐known that molecular events at the different layers are interdependent and collectively determine the state of the signaling system (Pawson, 1995; Deribe *et al*, 2010; Zheng *et al*, 2013; Ciuffa *et al*, 2022). An integrated view of the reorganization of the proteome across layers in the context of the cellular state is therefore critically important to unravel the underlying signaling network (Ciuffa *et al*, 2022). In this study, we developed a generic experimental and computational approach to study the interdependence between PPIs and phosphorylation in the context of signaling systems. Using the Hippo signaling system as a model, we combined genetic and chemical perturbation with protein and phospho‐protein correlation profiling following complex fractionation by AP‐BNPAGE, to identify different YAP1 proteoforms associated with distinct complexes, recapitulate known mechanisms of regulation and provide new insights into the PTPN14‐mediated inactivation of YAP1.

In the first step of the study, we used two different classes of phosphatase inhibitors for in‐depth profiling of YAP1 phosphorylation states and YAP1 protein interaction dynamics. The combined data evidenced how changes in YAP1 phosphorylation correlated with significant changes in the interactome; and how proteins known to be functionally related—for example, TEAD proteins, apicobasal proteins, and the F‐box proteins BTRC and FBXW11—undergo coordinated changes. We then combined AP‐BNPAGE with MS analysis to isolated distinct YAP1 complexes and obtain a more granular map of the phosphosite/PPI relationship. This approach, although successful in resolving the modular organization of YAP1 interactome, is limited by experimental and data analysis caveats that need to be emphasized. The experiment discussed required large amounts of cells (300 × 10^6^ cells per replicate) and considerable MS acquisition time (nearly 1 week of measurements). In addition, the separation by BNPAGE may disrupt the most labile interactions, as described for the assembly intermediates identified in modules 5, 7, and 8, and in the case of 14‐3‐3 proteins, which co‐migrate in a unique cluster at low molecular weight. Data analysis is challenged by co‐migrating complexes that might lead to convoluted modules, although to a lesser extent than in total protein profiling experiments. Furthermore, undersampling of low abundant phosphopeptides might compromise characterization of proteoforms and their assignment to a given module. Despite these limitations, the presented approach succeeded in the isolation of several complexes reported in the literature (i.e., RICH1 and AMOT polarity complex; Wells *et al*, 2006) or whose members are otherwise functionally related; and on top assigned distinct YAP1 phosphosites to each identified modules. By this means, our data reduce the number of potential YAP1 proteoform‐complexes associations by several orders of magnitude and paves the way for establishing causal relationships. In this sense, we consider our approach complementary to peptide‐based AP‐MS (Lundby *et al*, 2019), which identifies *in vitro* binding between synthetic phosphopeptides and cellular proteins, but fails to link protein level phosphorylation and complex formation. Phospho‐DIFFRAC (Floyd *et al*, 2021) has been proposed as an approach to study phosphorylation‐dependent complex reorganization combining size exclusion chromatography and mass spectrometry of the total proteome (SEC‐MS). However, the additional purification of complexes in AP‐BNPAGE as presented here, allows to resolve co‐migration patterns of low abundant proteins across multiple complexes that often are not resolved by proteome wide fractionation (Havugimana *et al*, 2012; Kirkwood *et al*, 2013; Bode *et al*, 2016; Heusel *et al*, 2019, 2020). In principle AP‐BNPAGE profiling can be applied to a broad range proteins, but may be particularly informative for proteins that, similar to YAP1, are subject to multisite modifications with a rich interactome under steady state and upon signaling. To measure signaling‐induced complex dynamics, AP‐BNPAGE analysis across multiple conditions will greatly benefit from multiplexed MS data acquisition techniques (Havugimana *et al*, 2022).

AP‐BNPAGE can assign phosphosites of a given protein to modules of interactors but does not establish which of the phosphorylation events are actually required for complex formation. Subsequent affinity purification with a set of YAP1 mutants carrying mutations in phospho‐sites identified by AP‐BNPAGE resulted in distinct, but also overlapping YAP1 protein interaction patterns. We confirmed the roles of well‐known sites (e.g., S127, S109), but also provided new functional insights for poorly studied sites (e.g., S367). However, YAP1, like most other proteins, can be modified at multiple sites that limits the mechanistic interpretation of single‐site mutational analysis since nonlinear and synergistic responses could result upon multisite phosphorylation.

Finally, we monitored changes in the phosphorylation status and interactome of YAP1 in a panel of cell lines lacking known Hippo regulators. The results confirm the role of LATS1/2 and NF2 as main modulators of YAP1 function and support the role of PTPN14 as an additional critical negative regulator of YAP1 transcriptional activity as demonstrated in several earlier studies and in different cell systems (Poernbacher *et al*, 2012; Wang *et al*, 2012, 2020; Huang *et al*, 2013; Liu *et al*, 2013; Michaloglou *et al*, 2013; Wilson *et al*, 2014). We verified by immunoblotting, immunofluorescence and proteomics that deletion of LATS1/2 and PTPN14 affect the activity and localization of YAP1 in similar ways, albeit at different magnitudes. Our targeted proteomics approach revealed a distinct pattern of hypophosphorylated YAP1 sites in PTPN14 KO cells that closely matches the one measured in LATS1/2 mutants and demonstrated that PTPN14 is required for the interaction of YAP1 with LATS1/2, highlighting the benefits of coordinately measuring changes in PPI and phosphorylation patterns. Furthermore, we show by reciprocal quantitative AP‐MS that PTPN14, YAP1, and LATS1 are binding to each other in a nonmutually exclusive fashion, indicating the existence of a trimeric complex. The existence of this PTPN14‐YAP1‐LATS1 complex was also apparent from our AP‐BNPAGE data, showing distinct co‐migration in a module (module 3, Fig 3B and C). AP‐XL‐MS indicated direct interaction between YAP1, PTPTN14 and LATS1 and AP‐MS with WW‐domain mutants suggest preferential binding of LATS1 and PTPN14 to the WW1 and WW2 domain, respectively. Taken together, these data suggest a model where PTPN14 may supports LATS1‐dependent YAP1 phosphorylation via WW domain‐mediated trimeric complex formation. It has been shown that increasing cell density and the extent of cell–cell contacts that is accompanied by a strengthened interaction of YAP1 with LATS1 and PTPN14, also leads to an increased phosphorylation of YAP1 (Zhao *et al*, 2007; Hauri *et al*, 2013). Given also the localization of PTPN14 at cell junctions (Wilson *et al*, 2014), it is conceivable that PTPN14, by promoting YAP1‐LATS binding may translate signals from cell–cell contacts to inhibit YAP1‐dependent transcription programs.

In summary, we describe a strategy to simultaneously analyze functional relationship of two critical mechanisms of cell signaling: PTMs and complex formation. Using YAP1 as a model, we integrated multiple proteomics layers to study (i) the role of phosphorylation for complex formation, (ii) how phosphorylation and complex formation are controlled by known pathway effectors, and (iii) how phenotypes could emerge from perturbing these signaling mechanisms. The steps described in this manuscript can be adapted to study the effect of different types of PTMs (i.e., ubiquitination) for a wide range of proteins that partition into multiple complexes. Besides representing a comprehensive and sensitive account of YAP1 phosphorylation and complex formation, our data suggest a model for PTPN14 as a negative YAP1 regulator via supporting binding and phosphorylation of YAP1 by LATS1/2. Given the widespread nature of phosphorylation controlled complex formation, we strongly believe that the presented strategy represents a significant analytical advance to disentangle regulatory mechanisms for a wide range of cellular signaling systems.

## Materials and Methods

### Reagents and Tools table

**Table:** 

Reagent/resource	Reference or Source	Identifier of catalog number
Experimental cell lines		
Flp‐Ln T‐Rex 293 Cell Line	Thermo Fisher Scientific	(Invitrogen) R78007
HEK293A	Thermo Fisher Scientific	(Invitrogen) R70507
HEK293A	Plouffe *et al* (2016)	NA
HEK293A YAP1KO	Plouffe *et al* (2016)	NA
HEK293A LATS1KO	Plouffe *et al* (2016)	NA
HEK293A LATS2KO	Plouffe *et al* (2016)	NA
HEK293A LATS1/2KO	Plouffe *et al* (2016)	NA
HEK293A RHOAKO	Plouffe *et al* (2016)	NA
HEK293A STK3/4/2KO	Plouffe *et al* (2016)	NA
HEK293A NF2KO	Plouffe *et al* (2016)	NA
HEK293A PTPN14KO	This study	NA
Oligonucleotides and sequence‐based reagents		
gRNA target sequence		Table EV1
Oligonucleotides		Table EV1
Recombinant DNA		
pTOSH‐GW‐FRT‐HA‐Strep	Glatter *et al* (2009)	NA
pOG44 Flp recombinase expression vector	Invitrogen	
pSpCas9(BB)‐2A‐GFP (PX458)	Addgene	48138 (PX458)
hORFeome V5.1	Horizon/Dharmacon	Open Biosystem
hORFeome V8.1	Horizon/Dharmacon	Open Biosystem
Antibodies		
Actin	Abcam	ab179467
YAP1 (commercial)	Santa Cruz	15407
YAP1 (custom developed)	Eurogentec (“speedy 28 Day program”)	
PTPN14	Cell Signaling	13808
YAP1 S127A	Cell Signaling	4911
HA	BioLegend	HA.11,901513
Mouse (Secondary)	Jackson Immuno Research Labs	115035003
Rabbit (Secondary)	Cell Signaling	7074S
Alexa488‐labeled secondary antibody	Thermo Fisher Scientific	A32731
Chemical and reagents		
Trypsin–EDTA	Life Technologies Europe BV	25300‐054
X‐tremeGENE 9 DNA Transfection Reagent	Sigma Aldrich Chemie	6365787001
Blasticidin S – Hydrochlorid	HUBERLAB.AG	A3784.0025
JetPrime	Polyplus	101000027
DMEM	Gibco	11965092
Penicillin–Streptomycin	Gibco	P0781‐100ML
Fetal Bovine serum (FBS) Mycoplasma and Virus screened 500 ml	BioConcept AG	2‐01F10‐I
Hygromycin B liquid (50 mg/ml) 20 ml	Invitrogen	10687010
Lipofectamine RNAiMAX	Thermo Fisher Scientific	13778‐150
cOmplete™, EDTA‐free Protease Inhibitor Cocktail	Roche	11873580001
Benzonase	Sigma Aldrich	E1014
Urea	Sigma Aldrich	U5128
Sodium Fluoride	Sigma‐Aldrich	201154
Sodium Orthovanadate	Sigma‐Aldrich	450243
Protease Inhibitor	Sigma‐Aldrich	P8849
PMSF	Sigma‐Aldrich	P7626‐5G
IGEPAL	Sigma‐Aldrich Chemie GmbH	8896
HEPES	Sigma Aldrich Chemie GmbH	H4034
NaCl	Merck	1.06404.5000
DSS	Thermo Fisher Scientific	
DSS (DSS‐d0, DSS‐d12)	Creative Molecules	
Avidin	IBA Life Sciences	2‐0204‐015
Strep‐Tactin Sepharose beads	IBA Life Sciences	
Bicinchoninic acid (BCA)	Pierce	
TCEP	Sigma‐Aldrich	C4706‐2G
IAA	Sigma‐Aldrich	I6125‐5G
Ammonium Bicarbonate	Sigma‐Aldrich	A6141
DTT	Sigma Aldrich	D0632
EDTA	Biosolve	5142391
Tris–HCl	Sigma Aldrich	10708976001
NuPAGE™ MES SDS Running Buffer (20×)	Invitrogen	NP0002
NuPAGE 4–12% Bis‐Tris SDS‐PAGE	Invitrogen	NP0321
Native PAGE 3–12%	Invitrogen	BN1001BOX
NitrocelluloseTrans‐Blot Turbo	BIoRad	1704158
Chemiluminescence kit	Cytiva	RPN3004
Simple Blue Safe Stain	Invitrogen	LC6060
Protease MAX Surfactant	Promega	V2071
Methanol	Fisher Chemicals	M/4058/17‐4
Acetonitrile	Fisher Chemicals	A995‐212‐4
Formic Acid, LC–MS grade	Pierce	28905
iRT peptides	Biognosys	
Glycerol	Chemie Brunschwig AG	15892‐0010
Ethanol	Honeywell	2860
Hydrochloric Acid	VWR International GmbH	1.00317.1000
Acetic Acid	VWR International GmbH	1.00063.1000
Okadaic acid	Biovision	1543
Vivacon 500 Sartorious 10KDa	Sartorious	VN01H01
Microspin	Nest group	
Biotin	Pierce	29129
HiTrap NHS‐Activated	GE Healthcare	
Protein A Sepharose 4 Fast Flow	Cytiva	17071601
Pur‐A‐Lyzer Mega Dialysis 3500KDa	Sigma Aldrich	PURG35010‐1KT
Sodium Borate	Sigma Aldrich	1.06669
Ethanolamine	Sigma Aldrich	398136
Triethylamine	Sigma Aldrich	T0886
DMP	Sigma Aldrich	80490
DAPI	Sigma Aldrich	D9542
RNeasy kit	Qiagen	74004
RNase‐free DNase I	Qiagen	79254
SuperScript II polymerase	Invitrogen	18064022
QIAprep Spin Miniprep Kit	QIAGEN	27104
Pierce™ BCA Protein Assay Kit	Thermo Fisher Scientific	23225
Software and algorithms		
MaxQuant 1.5.2.8	Cox & Mann (2008)	
Spectronaut 13	Bruderer *et al* (2015)	
Skyline (v.4.1)	MacLean *et al* (2010)	
Xquest/Xprophet	Leitner *et al* (2014)	
R 4.2.0	The R Project	

### Methods and Protocols

#### Plasmids and cloning

Expression constructs were generated with a N‐terminal Strep‐HA‐tagged bait proteins and entry clones of a Gateway compatible human clone collection (ORFeome v5.1 and v8.1). The integration of the entry clones into the Gateway destination vectors (pcDNA5/FRT/TO/SH/GW; Glatter *et al*, 2009) was performed with an enzymatic LR clonase reaction (Invitrogen). YAP1 mutant sequences were generated by gene synthesis in pDONR223 (Biocat GMBH) and subsequently cloned into pcDNA5/FRT/TO/SH/GW as described above. The sequences of YAP1 mutants oligonucleotides are reported in the Reagents and Tools table.

#### Tissue culture and DNA transfection

T‐REx™ Flp‐In cell lines purchased from Invitrogen were cultured in DMEM (4.5 g/l glucose, 2 mM l‐glutamine; Gibco), supplemented with 10% fetal bovine serum (FBS; BioConcept), 100 U/ml penicillin (Gibco), and 100 μg/ml streptomycin (Gibco). HEK293A cell lines were purchased from Invitrogen or received as gift by the Guan lab (Plouffe *et al*, 2016) were cultured in DMEM (4.5 g/l glucose, 2 mM l‐glutamine), supplemented with 10% FBS (BioConcept), 100 U/ml penicillin (Gibco), 100 μg/ml streptomycin (Gibco), and MEM Non‐Essential Amino Acids Solution. Cell lines were cultured at 37°C in a humidified incubator with 5% CO_2_.

#### Stable cell line generation of N terminal Strep‐HA‐tagged proteins

T‐REx™ Flp‐In cells were co‐transfected with the corresponding expression plasmid and the pOG44 vector (Invitrogen) encoding the Flp‐recombinase using jetPrime (Polyplus) according to the manufacturer's instructions. Two days after the transfection, cells were selected in hygromycin (100 μg/ml) and blasticidin C (15 μg/ml) containing medium for 3 weeks.

#### CRISPR/Cas9‐mediated gene knockout of PTPN14 in HEK293A cells

To generate CRISPR/Cas‐9 PTPN14 KO cells, we designed guideRNAs based on their specificity score from the Optimized CRISPR Design web tool (http://crispr.mit.edu; PTPN14 gRNA target sequence 1: 5′‐CACCGCGTTGTAGCGCCGTGTCCGGCGG (exon 1), PTPN14 gRNA target sequence 2: 5′‐CACCGGCTCCACCCATCGTGCTTGCTGG (exon 2)). Annealed DNA oligonucleotides containing the target sequence were cloned into the hSpCas9 plasmid (pX458, Addgene) using BbsI restriction sites. Subsequently, HEK293A cells were transfected with two hspCas9 constructs encoding gRNAs with the target sequence 1 and 2. The cell culture medium was replaced 4 h after transfection and cells were recovered for 72 h. Then, 1 × 10e6 cells were gently detached from the tissue culture plate with 0.25% trypsin–EDTA (Gibco) and resuspended in PBS containing 1% FBS. GFP‐expressing cells were detected and isolated by FACS (BD Facs Aria IIIu sorter) and sorted into a 96‐well plate. The cell clones were expanded and characterized by Western blotting and mass spectrometry.

#### Western blot

Cells were grown in six‐well plates to 80% confluency and harvested. Cell pellet was snap frozen and lysed in 100 μl lysis buffer (0.5% NP40, 50 mM HEPES (pH 7.5), 150 mM NaCl, 50 mM NaF, 400 nM Na_3_VO_4_, 1 mM PMSF and protease inhibitor cocktail). The cell lysate was cleared by centrifugation (15,000 *g* for 20 min), boiled for 5 min after the addition of 3× Laemmli sample buffer, loaded on NuPAGE 4–12% Bis‐Tris SDS–PAGE gels (Invitrogen) for gel electrophoresis and then transferred onto nitrocellulose membranes (Trans‐Blot Turbo, BioRad). The following primary antibodies were used: anti‐PTPN14 (#13808, Cell Signaling), anti‐actin (#179467, Abcam), anti‐YAP1 (#15407, Santa Cruz), anti‐YAP1phosphoS127 (#4911, Cell Signaling), and anti‐HA (HA.11,901513, BioLegend). Proteins were detected by enhanced chemiluminescence (ECL, Amersham) using horseradish‐peroxidase‐coupled secondary antibodies (Rabbit #7074, Cell Signaling and Mouse #115035003, Jackson ImmunoResearch).

#### Protein extraction and full proteome digestion

Cells were cultured in 150 mm tissue culture plates until they reach 80% confluence. Cells were harvested, and the cell pellet was snap frozen and lysed. Lysis was performed in 8 M urea and subjected to harsh sonication (3 times 1 min, 80% amplitude and 80% cycle time, Hielscher‐Ultrasound Technology), Benzonase (Sigma) activity (50 U/ml) and centrifugation (14,000 *g* for 15 min). The protein amount of the cleared supernatant was measured by the Bicinchoninic acid (BCA) assay (Pierce), and 50 μg protein was subsequently reduced (5 mM TCEP in 50 mM ammonium bicarbonate, 30 min) and alkylated (10 mM iodoacetamide, 30 min). The protein sample was diluted to 1.5 M urea and proteolyzed with 0.5 μg of LysC (Wako) and 2 μg Trypsin (Promega, sequencing grade) for 16 h at 37 °C. Proteolysis was quenched by 0.1% TFA and peptides were purified with a C18 column (Sep‐Pak 1 cc, Waters). Eluted peptides were dried using a speed vacuum centrifuge before being resuspended in 20 μl 0.1% formic acid and 2% acetonitrile. iRT peptides (Biognosys) were spiked to each sample (1:50) before LC–MS/MS analysis for quality control.

#### Affinity purification of Strep‐HA tagged proteins and digestion (AP‐MS)

The expression of Strep‐HA tagged bait proteins stably integrated in T‐REx™ Flp‐In cells was induced with 1 μg/ml tetracycline for 24 h. For affinity purification, three or four (based on bait expression), 150 mm tissue culture plates at 80% cell confluency were harvested and the cell pellet was snap frozen. The frozen pellet was lysed with the following buffer (HNN lysis buffer): 0.5% NP40, 50 mM HEPES (pH 7.5), 150 mM NaCl, 50 mM NaF, 400 nM Na_3_VO_4_ supplemented with 1 mM PMSF, 1.2 μM Avidin (IBA), and protease inhibitor cocktail (P8849, Sigma), using 800 μl of lysis buffer for each lysed cell plate. The lysate was incubated on ice for 20 min and subjected to mild sonication (3 times 10 s, 35% amplitude and 80% cycle time, Hielscher‐Ultrasound Technology) and digestion of nucleic acids via Benzonase (Sigma; 50 U/ml). The cleared cell lysate was incubated with 50 μl cross‐linked Strep‐Tactin Sepharose beads (IBA) for 1 h on a rotation shaker. Before the incubation with lysate, beads were cross‐linked with 5 mM of di‐succinimidyl suberate DSS (Thermo) in 50 mM HEPES (pH 8.0), 150 mM NaCl for 30 min at 37°C with strong agitation and quenched with 50 mM ammonium bicarbonate for 30 min at 37°C. Upon washing two times with lysis buffer and three times with HNN buffer (50 mM HEPES (pH 7.5), 150 mM NaCl, 50 mM NaF), beads and bound proteins were transferred in 10 kDa molecular weight cutoff spin column (Vivacon 500, Sartorious), following the FASP protocol (Wiśniewski *et al*, 2009). Briefly, beads in solution were centrifuged at 8,000 *g* until dryness. Samples were denatured, reduced (8 M Urea and 5 mM TCEP in 50 mM ammonium bicarbonate, 30 min), and alkylated (10 mM iodoacetamide, 30 min). Each sample was subsequently washed three times by flushing the filter with 25 mM ammonium bicarbonate and digested with 0.5 μg of Trypsin (Promega, sequencing grade) for 16 h at 37°C. Proteolysis was quenched by 0.1% TFA, and peptides were purified with a C18 microspin column (Nest Group). Eluted peptides were dried using a speed vacuum before being resuspended in 20 μl 0.1% formic acid and 2% acetonitrile. For quality control, iRT peptides (Biognosys) were spiked to each sample (1:50) before LC–MS/MS analysis. In fractionated samples, peptides were subjected to high pH fractionation in reversed phase (microspin column, Nest Group) following the procedure based on the high pH reversed‐phase peptide fraction kit (Pierce).

#### Affinity purification of Strep‐HA‐tagged proteins and crosslinking reaction (AP‐XL‐MS)

Strep‐HA‐tagged YAP1 was expressed and purified following the protocol described above. Briefly, after mild lysis, following purification, streptavidin beads containing YAP1 and co‐purified proteins were loaded in 10 kDa molecular weight cutoff spin column (Vivacon 500, Sartorious). The solution (without primary ammine) was concentrated to a final volume of ~ 100 μl and proteins subjected to cross‐linking reaction with 1 mM isotope labeled di‐summidyl suberate (DSS‐d0, DSS‐d12, CreativeMolecules Inc.) at 37°C for 30 min following the protocol preciously described (Leitner *et al*, 2014). The reaction was quenched with 50 mM ammonium bicarbonate, and beads in solution were centrifuged at 8,000 *g* until dryness. Subsequently, samples were denatured, reduced (8 M Urea and 5 mM TCEP in 50 mM ammonium bicarbonate, 30 min), alkylated (10 mM iodoacetamide, 30 min), washed three times by flushing the filter with 25 mM ammonium bicarbonate and digested with 0.5 μg of Trypsin (Promega, sequencing grade) for 16 h at 37 °C. Proteolysis was quenched by 0.1% TFA, and peptides were purified with a C18 microspin column (Nest Group). Eluted peptides were dried using a speed vacuum before being resuspended in 20 μl 0.1% formic acid and 30% acetonitrile and fractionated by peptide‐level size‐exclusion chromatography (SEC) using Superdex Peptide PC 3.2/300 (GE Healthcare). Three high‐mass fractions enriched in cross‐linked peptide pairs were dried and resuspended in 20 μl 0.1% formic acid and 5% acetonitrile and analyzed by MS in technical duplicated.

#### *In vivo* phosphatase inhibition

The expression of YAP1 N terminal Strep‐HA‐tagged integrated in T‐REx™ Flp‐In cells was induced with 1 μg/ml tetracycline. After 24 h, media was exchanged with growth media, and cells were stimulated with 100 μM and 150 nM of Vanadate and Okadaic acid (Biovision) for 2 or 20 min, and 60 or 150 min, respectively. Pervanadate was freshly prepared by mixing on ice for 20 min Na_3_VO_4_ (Sigma Aldrich) with H_2_O_2_ in a molar ratio 1:5, following the protocol of Huyer *et al* (1997). After stimulation, cells were harvested, and the cell pellet was snap frozen.

#### AP‐BNPAGE of YAP1 complexes (AP‐BNPAGE)

A visualized and detailed description of the protocol to resolve purified protein complexes is published by Pardo *et al* (2017) The experimental procedure described below underlines the important and critical steps to perform the experiment. For affinity purification coupled with Blue Native separation, fifteen 150 mm tissue culture plates at 80% cell confluency, treated with 1 μg/ml tetracycline for 24 h were harvested and the cell pellet was snap frozen. Cells were lysed, cleared, and incubated with 50 μl of Strep‐Tactin Sepharose beads, following the conditions described above for the affinity purification of Strep‐HA‐tagged proteins and digestion (AP‐MS). Upon washing two times with lysis buffer and three times with HNN buffer (50 mM HEPES (pH 7.5), 150 mM NaCl, 50 mM NaF), bound proteins were incubated for 30 min and eluted with 50 μl of 2.5 mM biotin in HNN buffer (Thermo). 40 μl of eluted protein was supplemented with 12 μl of native sample loading buffer and loaded on Native PAGE 3–12% Bis Tris precast protein gels (Invitrogen) for native separation, according to the manufacturer's instruction. Different from instructions, the cathode chamber was only filled with Light Blue Cathode Buffer. Native PAGE gel separation was performed for 3 h at 4°C applying three‐step gradient voltage (150–180–200 V). Once the separation was finished, proteins were stained with Simple Blue Safe Stain (Invitrogen) and proteolyzed following Protease MAX Surfactant (Promega) in gel digestion protocol. To excise 64 protein bands with the same size from a native gel separation (necessary for quantitative proteomics data), a custom device constituted by 100 parallel blades spaced 1 mm from one another was used. Briefly, protein bands were distained, shrunk, reduced (25 mM DTT), and alkylated (55 mm iodoacetamide) before proteolysis. Digestion was performed in 50 μl digestion solution (0.5 μg of Trypsin (Promega, sequencing grade), 0.1 μg of LysC (Wako), 0.01% ProteaseMAX Surfactant (Promega) in 50 mM ammonium bicarbonate). After overnight digestions, peptides extracted in solution were collected, while gel pieces were covered with 50% acetonitrile solution for 30 min to improve the yield of the peptide extraction. Peptide solutions generated from the proteolysis and from the treatment of gel pieces with 50% acetonitrile solution were dried and resuspended in 10 μl 0.1% formic acid and 2% acetonitrile. iRT peptides (Biognosys) were spiked to each sample (1:50) before LC–MS/MS analysis for quality control.

#### Immuno‐affinity purification using custom‐designed YAP1 antibodies. Design of epitope and beads preparation for IP‐MS

To perform antibody‐based purification, we designed a custom antibody against the C‐terminal region YAP1 (TLEGDGMNIEGEELM). The following parameter were determinant for the peptide choice: (i) exposition and lack of secondary structure (we used Psipred (Jones, 1999) as secondary structure prediction tool), (ii) low sequence homology with other human proteins, (iii) noninvolvement of PTMs and protein interactions, and (iv) peptide stability in solution (we used ProtParam Tool from Expasy to monitor the stability). The peptide was synthetized, coupled to KLH carrier protein and used for rabbit immunization with the “Speedy 28‐Day program” by Eurogentec. The final bleed was affinity purified in AKTA pure chromatography system (GE Healthcare) with the epitope antibody column with 50 mM HEPES (pH 7.5), 150 mM NaCl as running buffer and 0.1 M Glycine (pH = 3) for the elution. The column for the affinity purification was prepared coupling the peptide TLEGDGMNIEGEELM to NHS group of HiTrap NHS‐Activated affinity column (GE Healthcare). Eluate was neutralized in Tris base solution 100 mM, pH 8.8, dialyzed overnight in buffer 50 mM HEPES (pH 7.5), 150 mM NaCl using membrane dialysis tube (Pur‐A‐Lyzer Mega Dialysis 3500 kDa; Thermo). The dialyzed eluate and the flow through obtained from peptide affinity purification were quantified, affinity characterized, and coupled to protein A Sepharose 4 Fast Flow (GE Healthcare) following the protocol (Gersten & Marchalonis, 1978). Briefly, 10 mg of specific and unspecific antibodies were incubated with 5 ml of wet protein A Sepharose 4 Fast Flow beads for 1 h, and beads were extensively washed with 0.2 Sodium Borate pH = 9 and cross‐linked with 20 mM of DMP for 1 h. After quenching reaction with ethanolamine 0.2 M, beads were aliquoted (~ 200 μg of antibody per purification) and ready to use.

#### Immuno‐affinity purification using custom‐designed YAP1 antibodies (IP‐MS)

HEK293A and HEK293A with genetic deletions were cultured in ten 150 mm tissue culture plates to 80% confluency, harvested and the cell pellet was snap frozen. The frozen pellet was lysed in 8 ml of lysis buffer: 0.5% NP40, 50 mM HEPES (pH 7.5), 150 mM NaCl, 50 mM NaF, 400 nM Na_3_VO_4_ supplemented with 1 mM PMSF and protease inhibitor cocktail (P8849, Sigma). The lysate was incubated on ice for 20 min and subjected to mild sonication (3 times 10 s, 35% amplitude and 80% cycle time, Hielscher‐Ultrasound Technology) digestion of nucleic acids via Benzonase (Sigma; 50 U/ml). The cleared cell lysate was incubated with protein A beads coupled with antibodies overnight on a rotation shaker. After incubation, beads were washed and proteolyzed following the conditions described above for the affinity purification of Strep‐HA‐tagged proteins and digestion (AP‐MS).

#### IF analysis

A 200,000 HEK 293A cells were seeded on poly‐lysine coated glass coverslips and grown with the growth media as described above. After 24 h, cells were washed in ice‐chilled 1× PBS and fixed with 4% PFA. Permeabilizing with 0.1% Triton, cells were blocked with 5% filtered BSA containing 0.01% Triton for at least an hour. Cells were probed with anti‐YAP1 primary antibody (Santa Cruz Biotechnology, sc‐376830) at 1:100 dilution and alexa488‐labeled secondary antibody at 1:2,000 dilution. Before the final wash of coverslips with 1× PBS, cells were incubated with 1:3,000 DAPI for 10 min in the dark. Subsequently, the slides were mounted onto glass slides and imaged using inverted Nikon Eclipse Ti microscope. The nuclear relocation of YAP1 was imaged at 63× oil objectives. The acquisition of images in relevant channels was controlled using open‐source software *micromanager*. Z‐stack of images at multiple positions were acquired using the piezo drive and automated XY drive.

Image analysis was conducted using the CellProfiler software. Images in two channels—DAPI (nucleus) and Cy5 measuring YAP1 levels—were imported into the CellProfiler. Prior to analysis, illumination function was calculated in both channels by selecting the background function, block size of 60, and “Fit Polynomial” smoothing method. The correction function was calculated based on all images in each channel and subsequently applied to the corresponding channel to obtain illumination‐corrected images. The corrected DAPI image was used to segment the nucleus and define the “Nucleus” as a primary object. Propagating from coordinates of Nucleus into corrected YAP1 signal in Cy5 channel using “Global” threshold strategy, a secondary object encompassing the whole cell was created. Subtracting the Nucleus object from thus propagated cell, a tertiary object called “Cytoplasm” was created. Furthermore, two objects were created, expanding 2 pixels and 10 pixels from the nucleus. Subsequently, a tertiary objected called “ring” was created around the nucleus by subtracting 2‐pixel expanded nucleus from the 10‐pixel expanded nucleus. This ring was further limited within the cells by masking it within the coordinates of “Cytoplasm” object, defining it as “Perinuclear.” Finally, the median intensity of corrected YAP1 signal (Cy5 channel) was measured within the Nucleus and Perinuclear objects, and the ratio between the two was computed to determine relative nuclear relocation of YAP1. The experiment was repeated three independent times and more than 1,300 single cells from three repeats were analyzed per condition. Student's *t*‐test was performed between single‐cell data from each condition to determine the statistical significance. The significance is indicated with ***P < 0.001.

#### qPCR analysis

HEK293A cell lines (WT, LATS1/2KO and PTPN14KO) were grown in one 60 mm dish at 50% confluence. Cells were detached by trypsinization and lysed using QIAshredder columns (Qiagen). Total RNA was extracted using RNeasy kit (Qiagen), and DNA was degraded using RNase‐free DNase I (Qiagen) following the manufacturer's instructions. RNA was reverse transcribed into cDNA using random hexanucleotides (Microsynth) and SuperScript II polymerase (Roche). The relative abundance of CTGF and CYR61 mRNA was determined using a Roche LightCycler and SYBRgreen (Roche). GAPDH was used as a reference gene.

The oligonucleotides used for this experiment are reported in the Reagents and Tools table.

#### Mass spectrometry‐based data acquisition

##### MS data acquisition of *in vivo* phosphatase treatment of YAP1 Strep‐HA tagged

LC–MS/MS analysis was performed on an Orbitrap Elite mass spectrometer (Thermo Scientific) coupled to an Easy‐nLC 1000 system (Thermo Scientific). Peptides were separated on a Thermo PepMap RSLC column (15 cm length, 75 μm inner diameter) with a 60‐min gradient from 5 to 35% acetonitrile at a flow rate of 300 nl/min. The mass spectrometer was operated in data‐dependent acquisition (DDA) mode with the following parameters: one full FTMS scan (350–1,600 m/z) at 120,000 resolution followed by 15 MS/MS scans in the Ion Trap. Charge states lower than two and higher than seven were rejected. Selected ions were isolated using a quadrupole mass filter of 2.0 m/z isolation window. Precursors with MS signal that exceeded a threshold of 500 were fragmented (CID, Normalized Collision Energy 35%). Selected ions were dynamical excluded for 30 s.

##### MS data acquisition of AP‐BNPAGE of YAP1 complexes (AP‐BNPAGE)

LC–MS/MS analysis was performed on an Orbitrap Q Exactive HF mass spectrometer (Thermo Scientific), coupled to an Acquity UPLC M‐class system (Waters). Peptides were loaded on commercial trap column (Symmetry C18, 100 Å, 5 μm, 180 μm × 20 mm, Waters) and separated on a commercial column (HSS T3, 100 Å, 1.8 μm, 75 μm × 250 mm, Waters) using a 40‐min gradient from 8 to 30% acetonitrile at a flow rate of 300 nl/min. The mass spectrometer was operated in data‐dependent acquisition (DDA) mode with the following parameters: one full FTMS scan (350–1,500 m/z) at 60,000 resolution, 15 ms injection time and 3e6 AGC target, followed by 12 FTMS/MS scans at 60,000 resolution, 110 ms injection time and 1e5 AGC target. Charge states lower than 2 and higher than 7 were rejected. Selected ions were isolated using a quadrupole mass filter of 1.2 m/z isolation window and fragmented (HCD, Normalized Collision Energy 28%). Selected ions were dynamical excluded for 20 s.

##### MS data acquisition for targeted analysis of genetic KO screen in HEK293A cell lines

LC–MS/MS analysis was performed on an Orbitrap Q Exactive HF mass spectrometer (Thermo Scientific) coupled to an Acquity UPLC M‐class system (Waters). Peptides were loaded on commercial trap column (Symmetry C18, 100 Å, 5 μm, 180 μm × 20 mm, Waters) and separated on a commercial column (HSS T3, 100 Å, 1.8 μm, 75 μm × 250 mm, Waters) using a 90‐min gradient from 5 to 35% acetonitrile at a flow rate of 300 nl/min. The mass spectrometer was operated in parallel reaction monitoring (PRM) mode with the following parameters: one full FTMS scan (400–1,500 m/z) at 120,000 resolution, 250 ms injection time, and 3e6 AGC target, followed by time scheduled target PRM scans at 120,000 resolution, 247 ms injection time and 2e5 AGC target. Selected ions were isolated using a quadrupole mass filter of 2.0 m/z isolation window and fragmented (HCD, Normalized Collision Energy 30%). Scan windows were set to 10 min for each peptide in the final PRM method. The inclusion list with targeted peptides analyzed is reported (Dataset EV4).

##### MS data acquisition of YAP1 IP‐MS

LC–MS/MS analysis was performed on an Orbitrap Q Exactive HF mass spectrometer (Thermo Scientific) coupled to an Acquity UPLC M‐class system (Waters). Peptides were loaded on commercial trap column (Symmetry C18, 100 Å, 5 μm, 180 μm × 20 mm, Waters) and separated on a commercial column (HSS T3, 100 Å, 1.8 μm, 75 μm × 250 mm, Waters) using a 60‐min gradient from 2 to 37% acetonitrile at a flow rate of 300 nl/min. The mass spectrometer was operated in data‐dependent acquisition (DDA) mode with the following parameters: one full FTMS scan (350–1,500 m/z) at 60,000 resolution, 15 ms injection time, and 3e6 AGC target, followed by 12 FTMS/MS scans at 60,000 resolution, 110 ms injection time, and 5e4 AGC target. Charge states lower than two and higher than seven were rejected. Selected ions were isolated using a quadrupole mass filter of 1.2 m/z isolation window and fragmented (HCD, Normalized Collision Energy 28%). Selected ions were dynamical excluded for 30 s.

##### MS data acquisition of targeted analysis of YAP1 IP‐MS in HEK293A cell lines

LC–MS/MS analysis was performed on an Orbitrap Fusion Lumos Tribrid mass spectrometer (Thermo Scientific) coupled to an EASY‐nLC 1200 system (Thermo Scientific). Peptides were separated on Acclaim PepMap 100 C18 (25 cm length, 75 μm inner diameter) with a 90‐min gradient from 5 to 35% acetonitrile at a flow rate of 300 nl/min. The mass spectrometer was operated parallel reaction monitoring (PRM) mode with the following parameters: one full FTMS scan (200–2,000 m/z) at 30,000 resolution, 54 ms injection time, and 1e6 AGC target, followed by time scheduled target PRM scans at variable resolution and injection time (15,000 R/22 ms IT; 30,000 R/54 ms IT; 60,000R/118 ms IT; 120,000 R/246 ms IT). Selected ions were isolated using a quadrupole mass filter of 1.4 m/z isolation window and fragmented (HCD, Normalized Collision Energy 27%). Scan windows were set to 10 min for each peptide in the final PRM method. The inclusion list with target peptides analyzed is reported (Dataset EV6).

##### MS data acquisition of total protein expression in a panel of HEK293A cell lines with genetic deletion

Assay library generation: LC–MS/MS analysis was performed on an Orbitrap Fusion Lumos Tribrid mass spectrometer (Thermo Scientific) coupled to an EASY‐nLC 1200 system (Thermo Scientific). Peptides were separated on Acclaim PepMap 100 C18 (25 cm length, 75 μm inner diameter) with a 120‐min gradient from 3 to 35% acetonitrile at a flow rate of 300 nl/min. The mass spectrometer was operated in data‐independent acquisition (DDA) mode with the following parameters: one full FTMS scan (350–2,000 m/z) at 120,000 resolution (400 m/z), 50 ms injection time, and 4e5 AGC target, followed by 12 FTMS/MS scans at 30,000 resolution (400 m/z), 54 ms injection time, and 5e4 AGC target for a cycle time of 3 s. Charge states lower than two and higher than seven were rejected. Selected ions were isolated using a quadrupole mass filter of 1.4 m/z isolation window and fragmented (HCD, Normalized Collision Energy 35%). DIA. measurements: samples were analyzed with the same set up used for assay library generation. The mass spectrometer was operated in data‐independent acquisition (DIA) mode with the following parameters: one full FTMS scan (375–1,250 m/z) at 120,000 resolution, 50 ms injection time, and 4e5 AGC target, followed by 40 variable windows from 375 to 1,250 m/z with 1 m/z overlap at 30,000 resolution, 54 ms injection time, and 1e5 AGC target for a cycle time of 3 s. Precursor ions were fragmented with HCD, Normalized Collision Energy 35%.

##### MS data acquisition of targeted analysis of PTPN14, YAP1, LATS1, and GFP Strep‐HA‐tagged AP‐MS

LC–MS/MS analysis was performed on an Orbitrap Q Exactive HF mass spectrometer (Thermo Scientific) coupled to an Acquity UPLC M‐class system (Waters). Peptides were loaded on commercial trap column (Symmetry C18, 100 Å, 5 μm, 180 μm × 20 mm, Waters) and separated on a commercial column (HSS T3, 100 Å, 1.8 μm, 75 μm × 250 mm, Waters) using a 60‐min gradient from 5 to 35% acetonitrile at a flow rate of 300 nl/min. The mass spectrometer was operated parallel reaction monitoring (PRM) mode with the following parameters: one full FTMS scan (350–1,800 m/z) at 60,000 resolution, 110 ms injection time, and 1e6 AGC target, followed by time scheduled target PRM scans at 60,000 resolution, 119 ms injection time, and 2e5 AGC target. Charge states lower than two and higher than seven were rejected. Selected ions were isolated using a quadrupole mass filter of 2.0 m/z isolation window and fragmented (HCD, Normalized Collision Energy 30%). Scan windows were set to 6 min for each peptide in the final PRM method. The inclusion list with target peptides analyzed is reported. (Dataset EV8).

##### MS data acquisition Strep‐HA tagged YAP1 mutants

LC–MS/MS analysis was performed on an Exploris 480 mass spectrometer (Thermo Scientific) coupled to an Vanquish Neo liquid chromatography system (Thermo Scientific). Peptides were separated by C18 reverse phase column (75 μm ID × 400 mm New Objective, in‐house packed with ReproSil Gold 120 C18, 1.9 μm, Dr. Maisch GmbH) across 120‐min gradient from 5 to 35% buffer B at a flow rate of 300 nl/min (buffer A: 0.1% [v/v] formic acid, buffer B: 0.1% [v/v] formic acid, 80% [v/v] acetonitrile). The mass spectrometer was operated in data‐dependent acquisition (DDA) mode with the following parameters: one full FTMS scan (350–1,500 m/z) at 60,000 resolution (AGC target −100%, maximum Injection time auto) followed by 20 MS/MS scans in the Orbitrap (15,000 resolution, AGC target 200% and maximum injection time auto). Charge states lower than two and higher than seven were rejected. Precursors with MS signal that exceeded a threshold of 5000 were selected (isolation window ¼ m/z) and fragmented (HCD 28%). Selected ions were dynamical excluded for 25 s.

##### MS data acquisition for cross‐linked Strep‐HA‐tagged YAP1 (AP‐XL‐MS)

LC–MS/MS analysis was performed on an Orbitrap Elite mass spectrometer (Thermo Scientific) coupled to an Easy‐nLC 1000 system (Thermo Scientific). Peptides were separated on a Acclaim PepMap RSLC column (15 cm length, 75 μm inner diameter, Thermo) with a 90‐min gradient from 9 to 35% acetonitrile at a flow rate of 300 nl/min. (buffer A: 0.1% [v/v] formic acid 5% [v/v] acetonitrile; buffer B: 0.1% [v/v] formic acid, 95% [v/v] acetonitrile) The mass spectrometer was operated in data‐dependent acquisition (DDA) mode with the following parameters: one full FTMS scan (350–1,600 m/z) at 120,000 resolution followed by MS/MS scans in the Ion Trap. Only ions with charge higher than three were subjected to isolation (2.0 m/z isolation window) and fragmented (CID, Normalized Collision Energy 35%). Selected ions were dynamical excluded for 30 s.

#### Experiment design, data process, and statistical analysis of mass spectrometry data

##### Analysis of *in vivo* treatment of YAP1 Strep‐HA tagged with phosphatase inhibitors

The experiment was performed with three independent biological replicates of YAP1 Strep‐HA tagged purification without stimulation and with vanadate stimulation (2 and 20 min) or with okadaic acid stimulation (60 and 150 min). To identify YAP1 interactors, we analyzed 12 purification controls with GFP Strep‐HA tagged. Acquired spectra were searched using the MaxQuant software package version 1.5.2.8 embedded with the Andromeda search engine (Cox & Mann, 2008) against human proteome reference dataset (http://www.uniprot.org/, downloaded on 10.10.18) extended with reverse decoy sequences. The search parameters were set to include only full tryptic peptides, maximum one missed cleavage, carbamidomethyl as static peptide modification, oxidation (M) and phosphorylation (S, T, Y) as variable modification and “match between runs” option. The MS and MS/MS mass tolerance were set, respectively, to 4.5 ppm and 0.5 Da. False discovery rate of < 1% was used at the protein level to infer the protein presence. The protein abundance was determined from the intensity of top two unique peptides for each protein. Interactome definition: high confident interactors of AP‐MS experiments were determined by SAINTexpress (Teo *et al*, 2014) with default parameters using spectral counts obtained from Max Quant analysis (MS/MS Count). Twelve Strep‐HA‐GFP pulldowns processed and measured in parallel with the samples and additional control runs from the CRAPome database (http://crapome.org/; Mellacheruvu *et al*, 2013) were used to filter high confidence interactors of YAP1 (SAINT threshold score > 0.90). MS1 quantification of phosphorylated peptides: phosphorylated peptides were filtered based on Andromeda phospho localization probability score (> 0.8). Furthermore, phospho‐sites that were not detected in all three replicates in at least one condition were filtered out. Phosphopeptide intensities were bait normalized and missing value were imputed with the median of biological replicates (only one missing value per replicate per condition) was allowed. MS1 quantification of interactors: LFQ protein intensities of high confidence interactors were bait normalized and missing values were imputed with the median of biological replicates (only one missing value per condition) or using random sampling from a normal distribution generated 5% less intense values. Two‐sided *t*‐test and *P* (corrected for multiple hypothesis testing using the Benjamini–Hochberg method) were computed to compare treated and control groups. Cluster of kinetic profiles for interactors was performed with normalization to unstimulated samples and with a fuzzy cluster algorithm (mfuzzy package, R).

##### Analysis of AP‐BNPAGE of YAP1 complexes

The experiment was performed with single analysis of YAP1 Strep‐HA‐tagged purification, the eluate was separated with blue native gel and fractionated in 64 protein bands.

*Identification of YAP1 interactors*: three independent biological replicates of YAP1 Strep‐HA tagged purification were proteolyzed and peptides were fractionated using High pH Reversed‐Phase Fractionation Kit. Protein identified in fractionation samples were filtered using SAINT express, as described above, to obtain a deeper list of high confidence interactors (57 proteins). Proteins in the list of YAP1 interactors were considered for the AP‐BNPAGE experiment.

In the AP‐BNPAGE‐MS experiment, acquired spectra were searched using the MaxQuant software package using specification described above in the analysis of *in vivo* treatment of YAP1 Strep‐HA tagged with phosphatase inhibitors. Protein intensities of YAP1 interactors (high confidence interactors list from fractionated YAP1 interactome, Appendix Fig S2H) and phosphosite intensity of YAP1 and YAP1 interactors were extracted from the protein and peptide matrices. LFQ protein intensity was normalized using iRT peptide intensity; phospho peptides were filtered based on phospho localization probability score (filter peptides with score above 0.8 in at least one fraction; filter fractions with score above0.5) and the intensity was normalized using YAP1 protein abundance (only YAP1 phosphopeptides) and for iRT peptide intensity. Missing values were imputed with the average of two neighboring fractions. Phosphopeptide and protein profiles were normalized for the maximum value across the fractionation dimension. Next, each profile was split based on identified peaks using gaussian smoothing function (minimum normalized intensity 0.2 and width 2 for proteins; minimum normalized intensity 0.3 and width 2 for phosphopeptides). In the analysis of interactors, YAP1 was excluded as the protein is identified in all fractions and interacts with all protein groups identified in the separation. Hierarchical clustering based on the distance of peak correlation was performed for interactors and phospho‐sites to generate co‐migration groups. The number of clusters and the cluster stability was evaluated by the Silhouette plot using Euclidian distance of clusters.

For each identified cluster, we analyzed the Protein–Protein Interactions (PPIs) annotated in BioGRID (version 3.5.176; Oughtred *et al*, 2019) and compared them with those calculated for all combinations of YAP1 interactors (excluding YAP1 itself). Two generated distributions were assayed for normality with Shapiro test and with a two‐side unpaired *t*‐test. GO cellular component enrichment was calculated using hypergeometric test for the following terms: “cell junctions,” “cytoplasm,” “cytosol,” “apical plasma membrane,” and “nucleus cell compartments.” The layout of protein–protein interaction co‐migration groups (Figs 3C) was generated using Cytoscape (v3.6.0; Kohl *et al*, 2011).

##### Analysis of targeted quantification of genetic KO screen in HEK293A cell lines

The experiment was performed in three independent biological replicates. Dataset EV4 reports the list of all target peptides and proteins measured in the analysis. PRM assay containing protein knockout in the cell line panel, housekeeping protein (Actin B), and iRT peptides was generated from spectra library data imported in Skyline (v.4.1; MacLean *et al*, 2010). Spectra libraries were built using published spectral libraries (Rosenberger *et al*, 2014) and Mascot search results (v. 2.4.1, MatrixScience) after proteomic analysis of cell lysates and YAP1 affinity purified as described above. Briefly, for Mascot search with precursor tolerance of 15 ppm and fragment tolerance of 0.6 Da, a Mascot score larger than 20 and an expectation value smaller than 0.05 were considered to identify correctly assigned peptides. Peak group identification and automatic peak picking of six fragment per peptide was performed employing the mProphet (Reiter *et al*, 2011) algorithm. The second best peaks were used as controls for the training model. For peptide identification, we used the following criteria: retention time matching to spectra library within 5% of the gradient length and dot product between library spectra intensities and light peptides > 0.75. After identification, peptide abundance was obtained from the sum of the integrated area of three fragment ions per peptide. Fragment ions with a signal‐to‐noise ratio less than 5 were filtered out for the quantification. Peptide values were normalized for the intensity of housekeeping peptides (Actin B) and for the intensity of iRT peptides.

##### Analysis of YAP1 IP‐MS

The experiment was designed with YAP1 immuno‐purification and two different control purifications (co‐immuno purification with unspecific antibodies in HEK293A wt cells and anti‐YAP1 co‐immunopurification in YAP1KO HEK293A cells). All purifications were performed in three independent biological replicates. All samples were fractionated with reverse phase high pH fractionation kit (Pierce). Acquired spectra were searched using the MaxQuant software package using specification described above in the analysis of *in vivo* treatment of YAP1 Strep‐HA tagged with phosphatase inhibitors. Proteins significative upregulated (*P*‐value with Benjamini and Hochberg method correction < 0.05) in YAP1 immunopurifications with both purifications were considered as YAP1 interactors.

##### Targeted YAP1 IP‐MS analysis in HEK293A cell lines

The experiment was performed with three independent biological replicates of YAP1 endogenous immune‐purified from a panel of cell lysates.

For the targeted assay/panel, selected peptides belong to proteins, which were prior characterized within this study as high confidence interactors (identified in AP‐MS and IP‐MS experiments) were considered. This targeted panel was supplemented with YAP1 phosphopeptides (identified in AP‐MS and IP‐MS experiments). Dataset EV6 reports the list of all target peptides and proteins measured in the analysis. Isotope‐labeled heavy peptides corresponding to the proteotypic peptides selected for this study, and containing either heavy lysine (13C(6) 15N(2)) or arginine (13C(6) 15N(4)) residues were purchased from JPT Peptide Technologies GmbH. Peptides were analyzed manually, and correct identification with six fragment ions per peptide was assigned based on the coelution of light and heavy peptide and matching peak shape for precursor and product ions from light and heavy peptides. The abundance of peptides was analyzed by summing the integrated areas of three fragment ions per peptide. Fragment ions with a signal‐to‐noise ratio less than 5 were filtered out for the quantification. Peptide intensity values were normalized for the intensity of 8 YAP1 peptides, for the TIC and for the intensity of iRT peptides. Significance of change in intensity was estimated with *P* values using two‐sided, not paired *t*‐test.

##### Analysis of total protein expression in a panel of HEK293A cell lines with genetic deletion

Differential protein expression of three independent biological replicates was measured by data‐independent acquisition (DIA). For library generation, a single‐cell lysate from HEK293A was proteolyzed and peptides were fractionated and analyzed in DDA mode. Hybrid spectral library was generated by Spectronaut 13 (version 13.2.190709; Bruderer *et al*, 2015; Biognosys) using peptide identified in the DIA runs and peptides identified in DDA mode from a prior offline fractionation (8 fractions, reverse phase high pH fractionation). For DDA analysis, acquired spectra were searched using the MaxQuant software package using specification described above in the analysis of *in vivo* treatment of YAP1 Strep‐HA tagged with phosphatase inhibitors, excluding threonine, tyrosine, and serine phosphorylation as variable modification. The generated library included entries for 120,844 peptide precursors and 8,408 protein groups. For the DIA analysis, extraction of quantitative data was performed with Spectronaut querying the library above mentioned with the following settings: tolerance of 10 ppm for precursor and 25 ppm for fragment ions and a dynamic retention time extraction window with nonlinear iRT retention time calibration. Precursor and proteins were identified with q value cutoff of 0.01 (5,947 protein groups). Data normalization by total ion current (TIC) and filtering was performed with mapDIA (Teo *et al*, 2015), where a standard deviation factor of 2 and a minimal correlation of 0.2 were used to filter robust fragment ions with minimum intensity threshold of 200. Filter strategy at protein level include minimum identification of two peptides per protein group in at least two of the three biological replicates. Group comparison level between each of the seven conditions against the wild‐type HEK293A signal was performed within mapDIA. We identified 31,295 and 4,436 proteins with only 3% of missing values across the matrix. Missing values were imputed with the median value of biological replicates (only one missing value per condition) or using random sampling from a normal distribution generated 1% less intense values. ANOVA statistical test was performed to compare protein profiles in all different cell lines.

##### Analysis of targeted PTPN14, YAP1, LATS1, and GFP Strep‐HA‐tagged AP‐MS

The experiment was performed with three independent biological replicates of YAP1, PTPN14, LATS1, GFP Strep‐HA‐tagged purifications. Dataset EV8 reports the list of all target peptides. Selected peptides from the YAP1 library were identified and quantified with the same criteria described above in the analysis of targeted YAP1 IP‐MS in HEK293A cell lines. Peptide intensity values were normalized for the intensity of a reference peptide in the Strep‐HA‐tag of all bait proteins (AADITSLYK) and for the intensity of iRT peptides.

##### Analysis of Strep‐HA‐tagged YAP1 mutants

The experiment was performed with three independent biological replicates and acquired spectra were searched using the MaxQuant software package version 1.5.2.8 embedded with the Andromeda search engine against human proteome reference dataset (http://www.uniprot.org/, downloaded on 06.04.2021) extended with reverse decoy sequences. The search parameters were set to include only full tryptic peptides, maximum two missed cleavage, carbamidomethyl as static peptide modification, oxidation (M) and deamidation (N‐ter) as variable modification and “match between runs” option. The MS and MS/MS mass tolerance was set to 20 ppm. False discovery rate of < 1% was used protein identification. Protein abundance was determined from the intensity of top two unique peptides. Intensity values were median normalized and imputed using random sampling from a normal distribution generated 1% less intense values. ANOVA statistical tests were performed to compare interactor profiles in all conditions. *P*‐values were corrected using the Benjamini–Hochberg method.

##### Analysis of AP‐XL‐MS for Strep‐HA tagged YAP1

The experiment was performed with two independent biological replicates and with two technical replicates. Data were converted to mzXML format with msConvert and searched with xQuest/xProphet (Leitner *et al*, 2014) against a database containing the fasta sequence of YAP1, PTPN14 and LATS1 and its decoy sequence. xQuest search parameters were search for tryptic peptides with two maximum missed cleavages and initial mass tolerance of 15 ppm. The mass of cross‐linker addition is 138.068080 Da. Cross‐linked peptides with a minimal length of five amino acids and a xQuest ld (linear discriminant) score higher than 20 (with a FDR determined with xProphet lower than 0.05) were considered. Cross‐linked peptides are visualized with xvis (web server, https://xvis.genzentrum.lmu.de; Grimm *et al*, 2015). Results are reported in Dataset EV9.

## Author contributions

**Federico Uliana:** Conceptualization; data curation; formal analysis; validation; investigation; visualization; methodology; writing – original draft; writing – review and editing. **Rodolfo Ciuffa:** Data curation; visualization; writing – original draft; writing – review and editing. **Ranjan Mishra:** Investigation. **Andrea Fossati:** Formal analysis; investigation. **Fabian Frommelt:** Formal analysis; investigation. **Sabrina Keller:** Investigation. **Martin Mehnert:** Investigation. **Eivind Birkeland:** Investigation. **Frank van Drogen:** Conceptualization; resources; supervision; funding acquisition; investigation; writing – original draft; project administration; writing – review and editing. **Nevena Srejic:** Investigation. **Matthias Peter:** Investigation; writing – original draft. **Nicolas Tapon:** Writing – original draft. **Ruedi Aebersold:** Resources; funding acquisition; writing – original draft. **Matthias Gstaiger:** Conceptualization; resources; supervision; funding acquisition; writing – original draft; project administration; writing – review and editing.

## Disclosure and competing interests statement

The authors declare that they have no conflict of interest.

## Supporting information



Appendix

Expanded View Figures PDF

Table EV1

Dataset EV1

Dataset EV2

Dataset EV3

Dataset EV4

Dataset EV5

Dataset EV6

Dataset EV7

Dataset EV8

Dataset EV9

PDF+

## Data Availability

Raw proteomics files are deposited to ProteomeXchange Consortium via the PRIDE partner repository (Perez‐Riverol *et al*, 2022) with identifiers PXD032218 (http://www.ebi.ac.uk/pride/archive/projects/PXD032218; Analysis of *in vivo* treatment of YAP1 Strep‐HA tagged with phosphatase inhibitors, Fig 2), PXD030061 (http://www.ebi.ac.uk/pride/archive/projects/PXD030061; Analysis of AP‐BNPAGE of YAP1 complexes, Fig 3), PXD030137 (http://www.ebi.ac.uk/pride/archive/projects/PXD030137; Analysis of YAP1 IP‐MS, Fig 4), PXD032220 (http://www.ebi.ac.uk/pride/archive/projects/PXD032220; Analysis of targeted quantification of genetic KO screen in HEK293A cell lines, Fig 4), PXD032310 (http://www.ebi.ac.uk/pride/archive/projects/PXD032310; Targeted YAP1 IP‐MS in HEK293A cell lines, Fig 4), PXD030368 (http://www.ebi.ac.uk/pride/archive/projects/PXD030368; Analysis of total protein expression in a panel of HEK293A cell lines with genetic deletion, Fig 5), PXD032221 (http://www.ebi.ac.uk/pride/archive/projects/PXD032221; Analysis of targeted PTPN14, YAP1, LATS1 and GFP Strep‐HA tagged AP‐MS, Fig 5), PXD039443 (http://www.ebi.ac.uk/pride/archive/projects/PXD039443; Analysis of YAP1 mutants by AP‐MS, Figs 3 and 5) and PXD039440 (http://www.ebi.ac.uk/pride/archive/projects/PXD039440; Crosslinking analysis of YAP1, Fig 5).
