# Supplementary material for: Phosphorylation‐linked complex profiling identifies assemblies required for Hippo signal integration
Source: Mol Syst Biol. 2023 Mar 10;19(4):e11024. doi: 10.15252/msb.202211024 (PMC10090947; doi:10.15252/msb.202211024)
Supplement: Supplementary file 1 — Appendix [file MSB-19-e11024-s010.docx]

Appendix to:

**Phosphorylation-linked complex profiling identified assemblies required for Hippo signal integration**

Federico Uliana^1,2#^*, Rodolfo Ciuffa^1#^, Ranjan Mishra^2^, Andrea Fossati^1,3,4,5^, Fabian Frommelt^1^, Sabrina Keller^1^, Martin Mehnert^1^, Eivind Salmorin Birkeland^2^, Frank van Drogen^2^, Nevena Srejic^2^, Matthias Peter^2^, Nicolas Tapon^6^, Ruedi Aebersold^1^, Matthias Gstaiger^1^*

1 Department of Biology, Institute of Molecular Systems Biology, ETH Zurich, Zurich, Switzerland

2 Department of Biology, Institute of Biochemistry, ETH Zurich, Zurich, Switzerland

3 University of California San Francisco, Quantitative Biosciences Institute (QBI),

San Francisco, CA, 94158, USA

4 University of California San Francisco, Department of Cellular and Molecular Pharmacology, San Francisco, CA, 94158, USA

5 J. David Gladstone Institutes, San Francisco, CA 94158, USA

6 Apoptosis and Proliferation Control Laboratory, The Francis Crick Institute, 1 Midland Road, London, NW1 1AT, UK

* Correspondence: Correspondence to Matthias Gstaiger and to Federico Uliana

^#^ Contributed equally

**Table of Content:**

| Appendix Figures S1 | 2 |
| --- | --- |
| Appendix Figures S2 | 3 |
| Appendix Figures S3 | 4 |
| Appendix Figures S4 | 5 |
| Appendix Figures S5 | 6 |
| Appendix Figures S6 | 8 |
| Appendix Figures S7 | 9 |
| Appendix Figures S8 | 11 |
| Appendix Figures S9 | 13 |
| Appendix Figures S10 | 14 |
| Appendix Figures S11 | 15 |


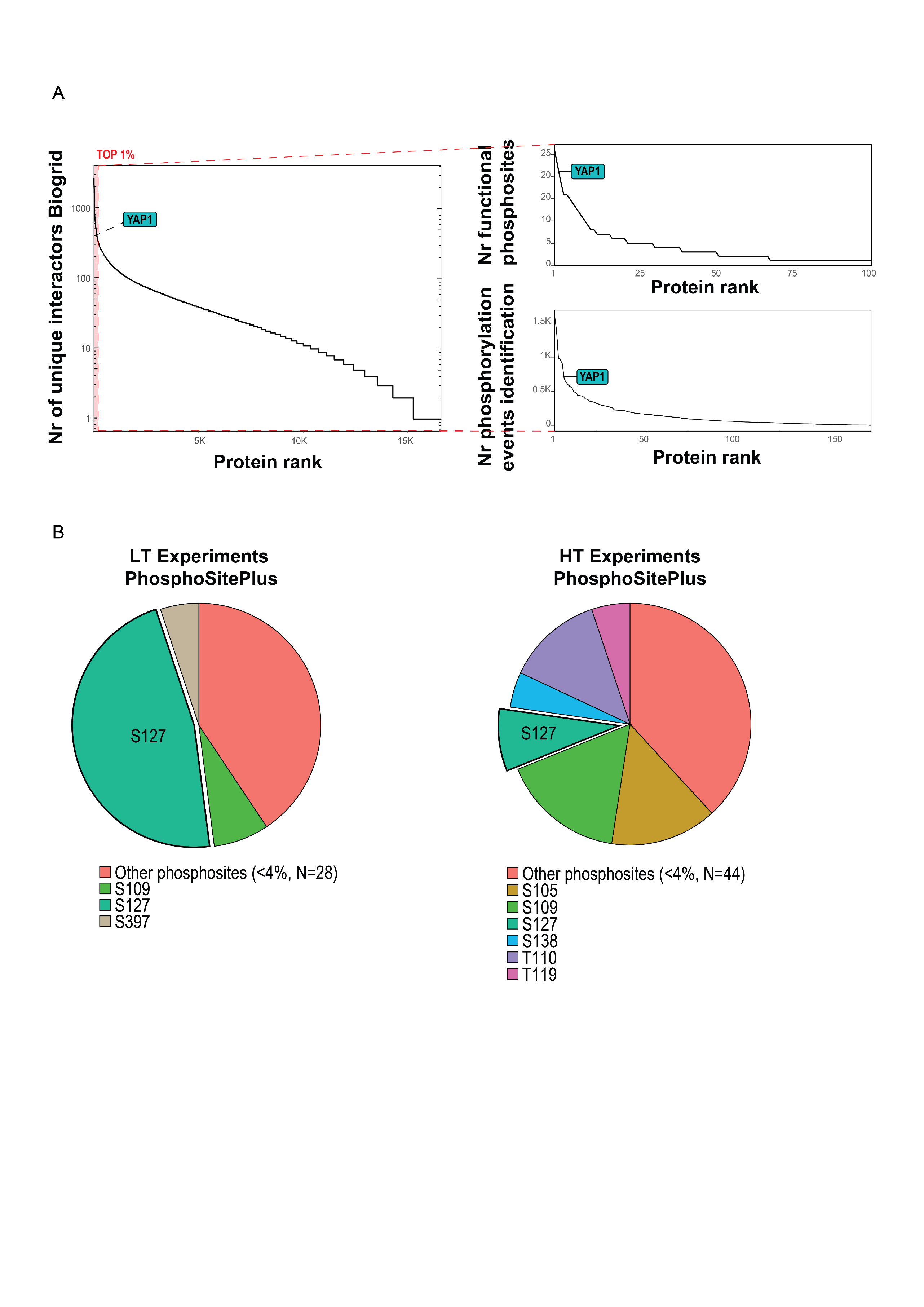


**Appendix Figure S1 A.** Selection of YAP1 as a model system was based on the number of known interactors (annotated in *BioGRID*; left), and number of identified and functional phosphosites data (based on *Phosphositesplus* and the scoring system proposed in Ochoa et al., 2019, respectively). **B.** Fraction of experiments (low throughput, left and high throughput, right) in which YAP1Phosphosites are annotated (*Phosphositesplus*, CST).


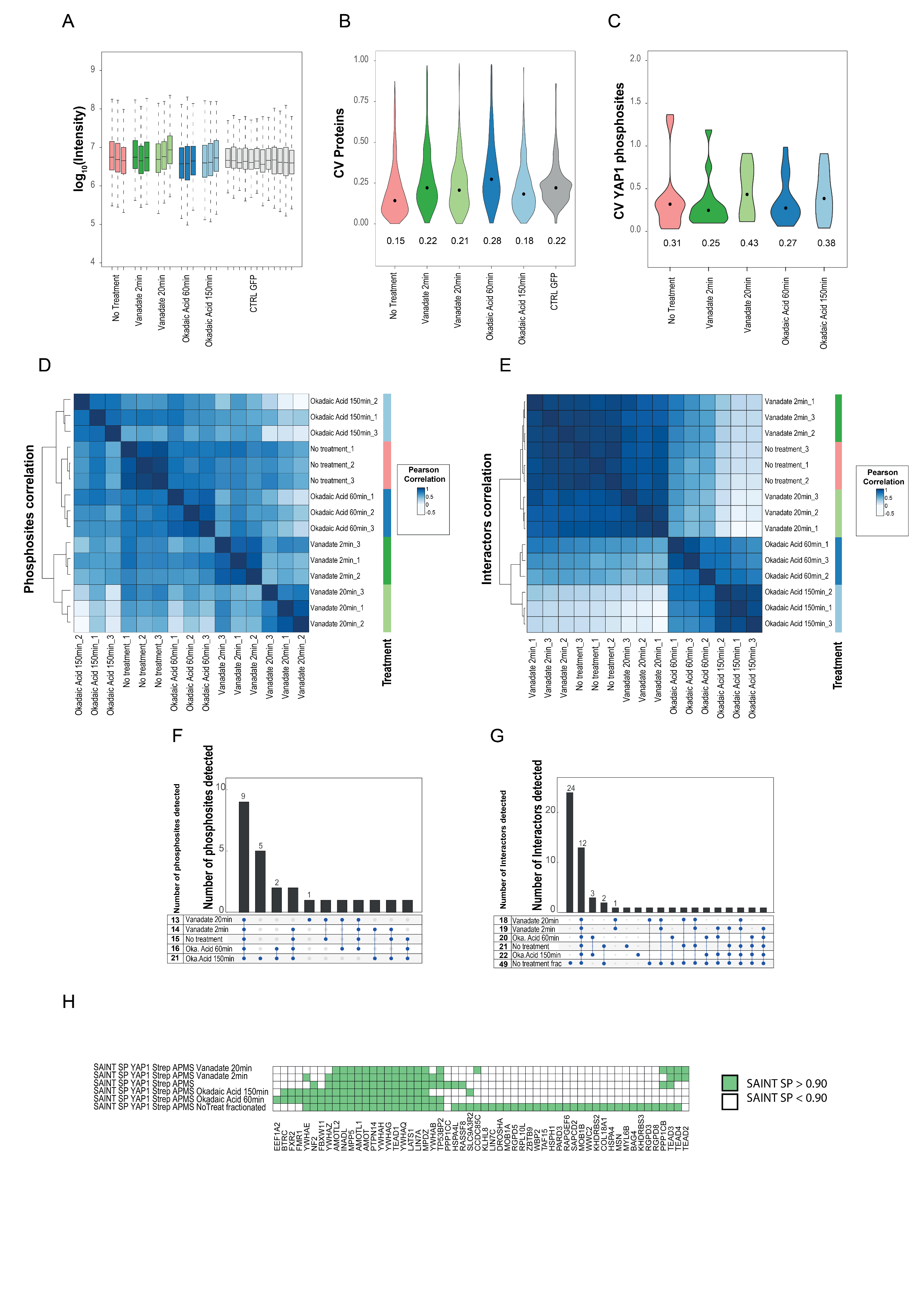


**Appendix Figure S2. A.** Distribution intensity (log10) of identified proteins. **B.** Distribution of coefficient of variation (CV) values at protein level. **C.** Distribution of coefficient of variation (CV) values for YAP1 phosphosites. **D.** Unsupervised hierarchical cluster based on Pearson correlation for YAP1 phosphosites identified across the tested conditions. **E.** Unsupervised hierarchical cluster based on Pearson correlation between YAP1 interactors identified across the tested conditions. **F.** Upset plot of size and overlap of phosphosite sets identified across the tested conditions. **G.** Upset plot of size and overlap of interactor sets identified across the tested conditions. **H.** Heatmap of Strep-HA YAP1 high confidence interactors identified using a SAINT SP score threshold of 0.90 in the tested condition.


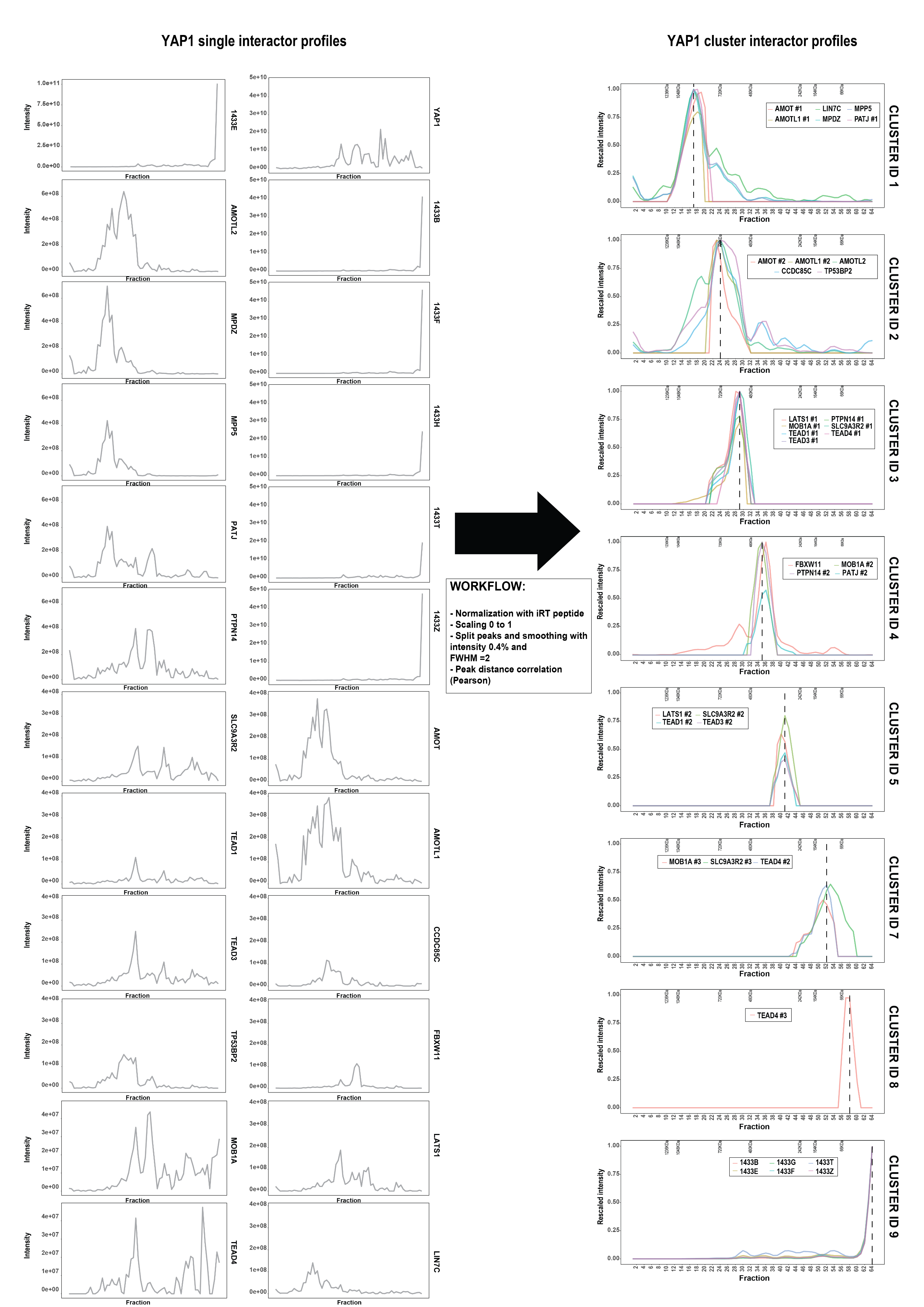


**Appendix Figure S3.** Raw intensity of AP-BNPAGE profiles of YAP1 interactors (left) and profiles after data analysis processing (scaling, split and peak smoothing) grouped by cluster membership (right).


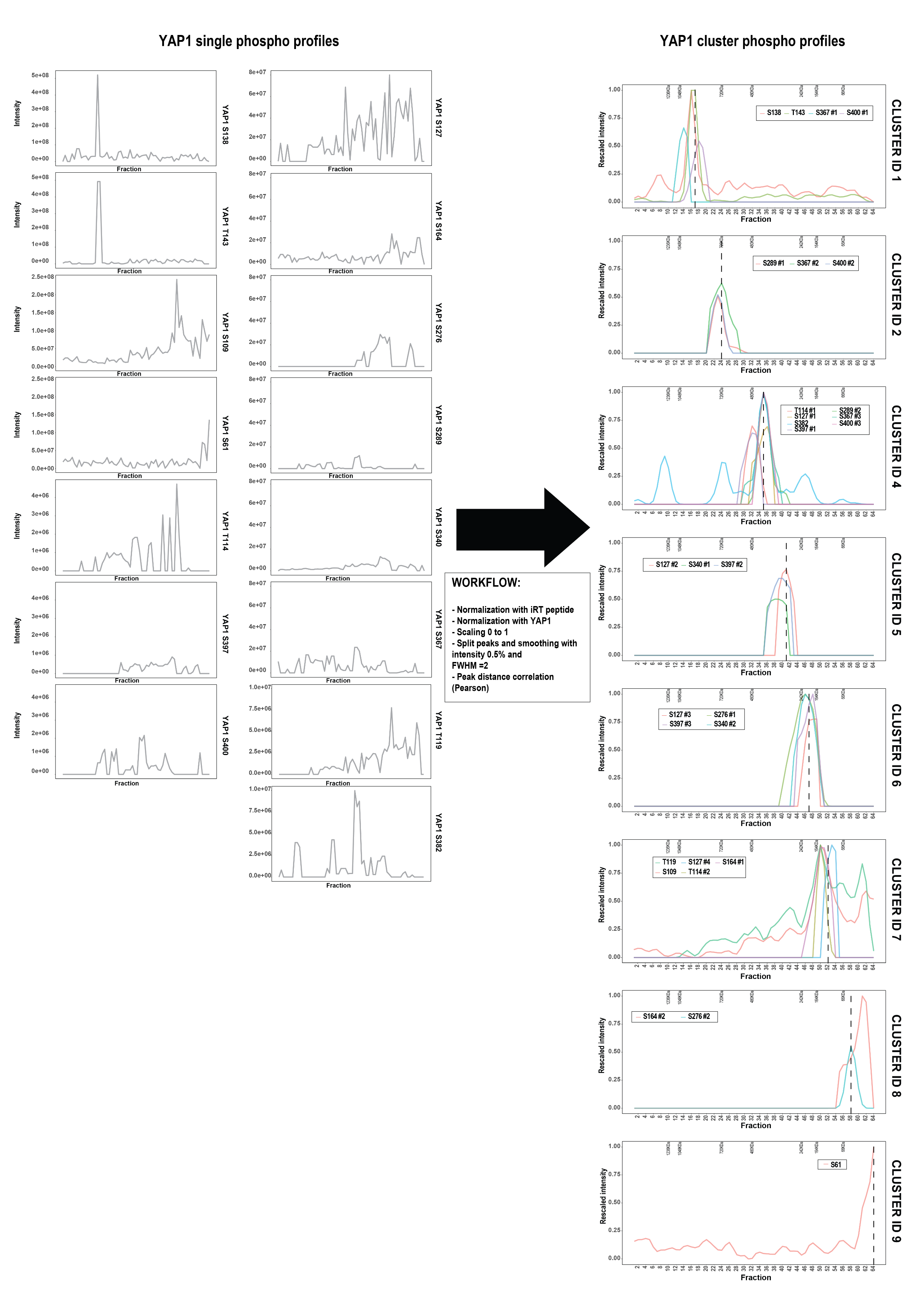


**Appendix Figure S4.** Raw intensity of AP-BNPAGE profiles of YAP1 phosphosites (left) and profiles after data analysis processing (scaling, split and peak smoothing) grouped by cluster membership (right).


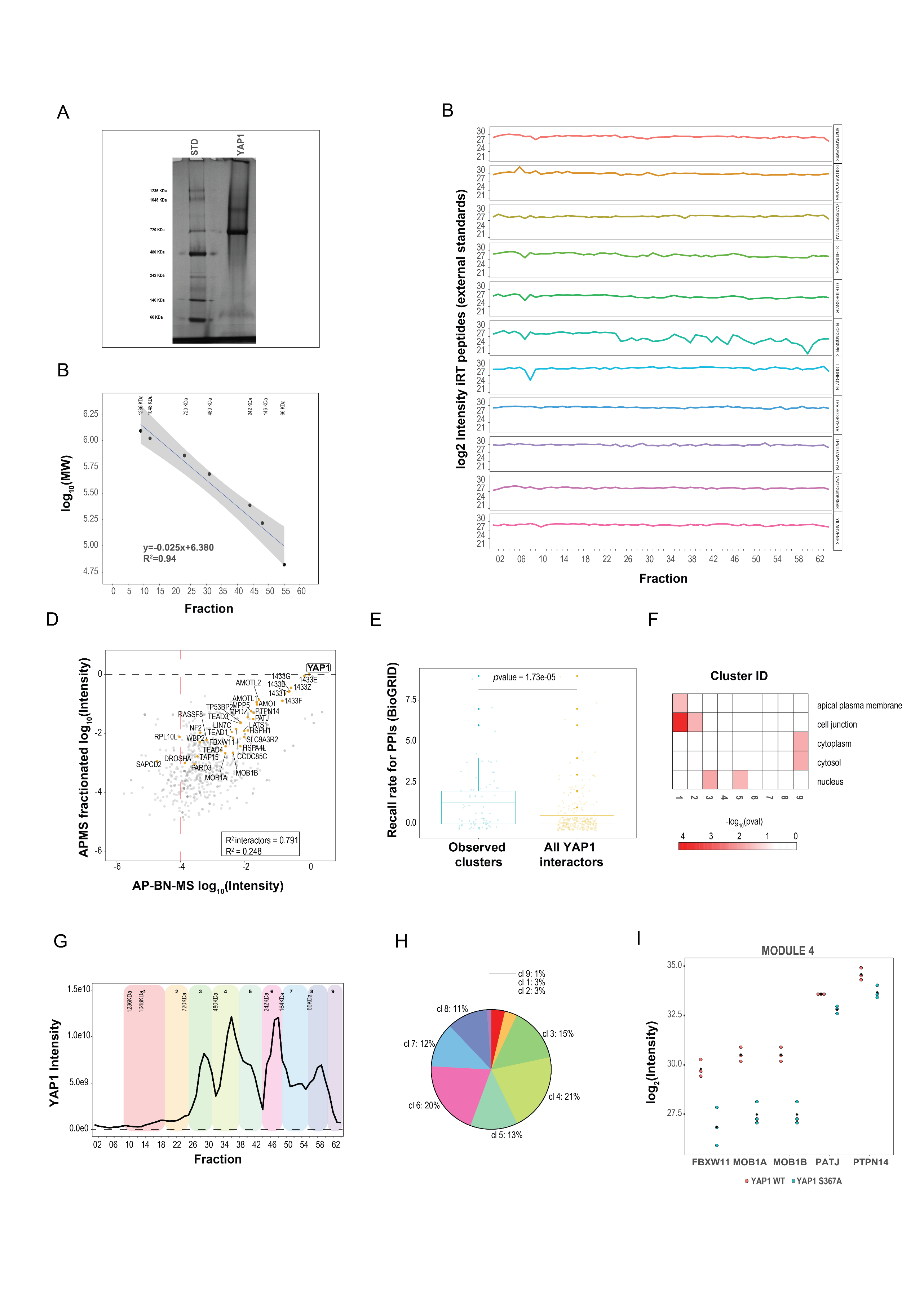


**Appendix Figure S5** **A.** Coomassie staining of the BNPAGE used to resolve YAP1 complexes. **B.** Calibration curve with external standards (BNPAGE proteins standard) separated on AP-BNPAGE. Calibration curve is used to estimate the molecular weight of YAP1 modules. **C.** Quantitative value of external standard (iRT peptides) spiked over all 64 fractions. Quantitative values are obtained from the integration of MS1 signal intensity. **D.** Protein intensity correlation between YAP1 AP-MS and AP-BNPAGE (generated from the sum in all measured fractions of each protein intensity). R^2^ for YAP1 interactors (0.791) and background proteins (0.248) is reported in the lower box. **E.** Recall rate (BioGRID) for protein-protein interaction pairs in co-migrating cluster compared with all interactions pairs of YAP1 interactors. Significancy is evaluated computing a two-side unpaired t-test assuming normal distribution. The boundaries of the box plot correspond to the quantiles Q1 (25%) and Q3 (75%). Lower and upper whiskers are defined by Q1 −1.5IQR and Q3+ 1.5IQR . **F.** Cellular component terms (GO) enriched in the identified modules indicates discrete protein localization for the following terms (nucleous, cytosol, cytoplasm, cell junction and apical plasma membrane). Values reported in the heatmap represent the -log10 *p*value of the hypergeometric test. **G.** Distribution of smoothed signal of Strep-HA YAP1 MS1 intensity across 64 AP-BNPAGE fractions. **H.** Relative Strep-HA YAP1 intensity associated with each of the identified clusters. **I.** MS1 intensity of YAP1 interactors identified in module number 4. Three independent replicates of S367A Strep-HA YAP1 mutant (cyan) is compared to wild type Strep-HA YAP1 (red).


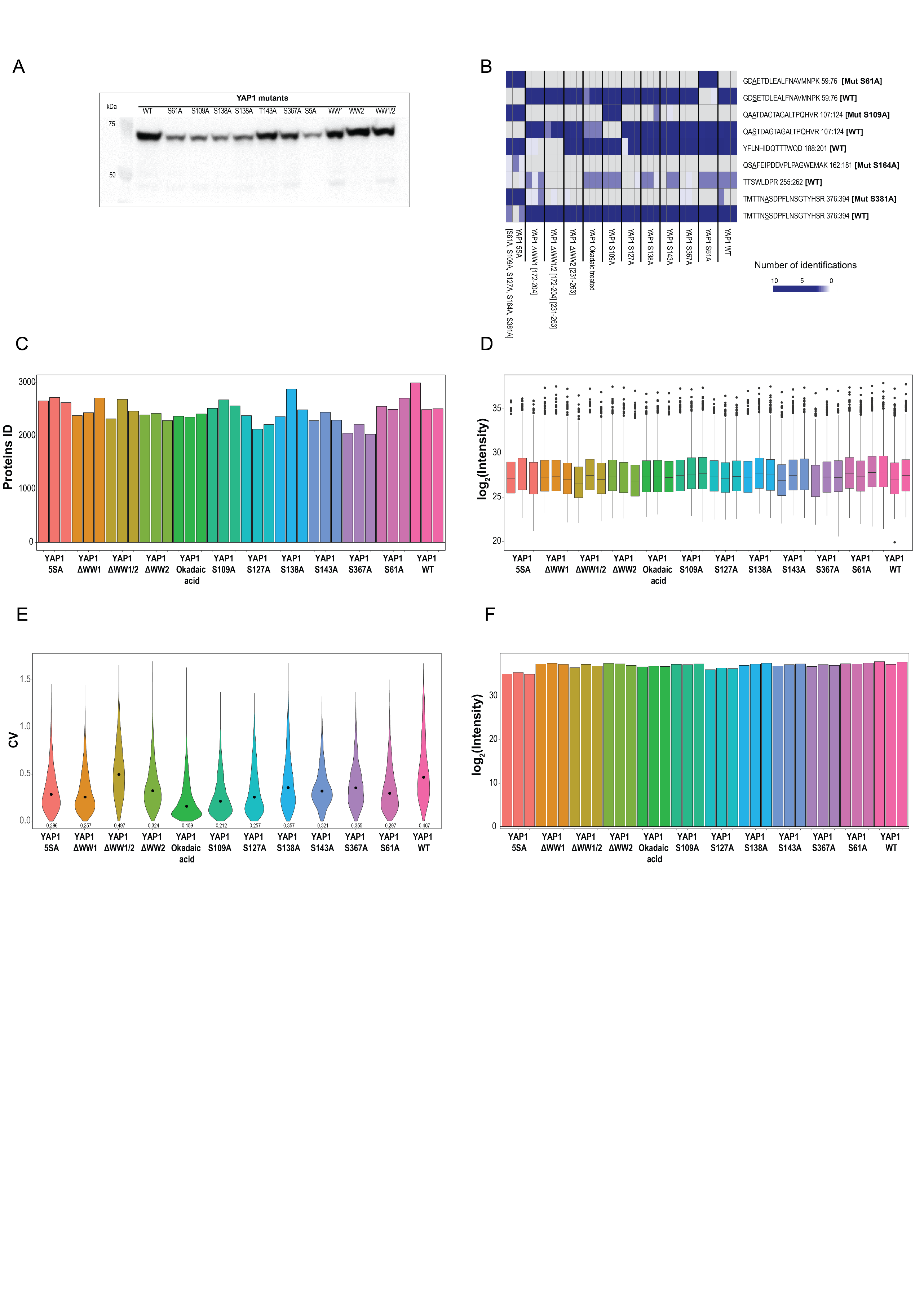


**Appendix Figure S6.** Intensity profile of YAP1 interactors in a panel of YAP1 mutants. The panel of mutants is composed by Strep-HA YAP1 phospho mutants (6 single mutants and one with multiple mutations S5A) and WW domain deletions (ΔWW1, ΔWW2 and ΔWW1/2). Interactors are identified and quantified by MS1 intensity after Strep affinity purification of Strp-HA YAP1 **A.** Validation of inducible expressed cell lines with YAP1 mutants. **B.** Number of identifications for peptides which are specific for YAP1 mutants. Blue scale is proportional to the number of identifications. Peptides not identified are shown in grey. **C.** Number of identified proteins in the YAP1 mutant dataset. **D.** Distribution of protein intensity (log10) in the YAP1 mutant dataset. **E.** Distribution of coefficient of variation (CV) values. **F.** YAP1intensity (expressed in log2 scale) across the different YAP1 mutants.


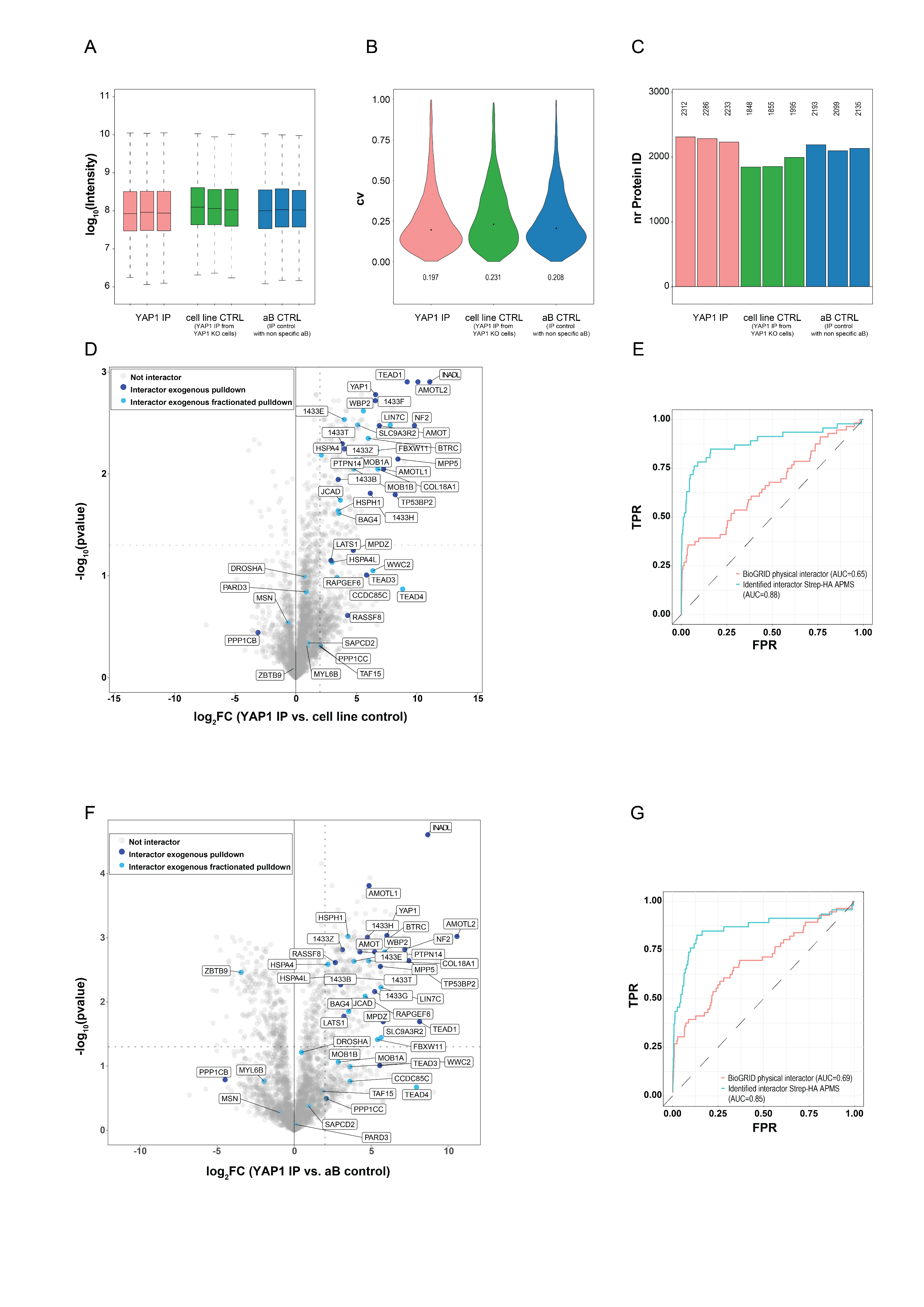


**Appendix Figure S7.** YAP1 endogenous interactome identified by YAP1 immuno-affinity purification (IP-MS). **A.** MS1 Intensity distribution (log10) of proteins identified in the indicated purifications (left: YAP1 IP-MS, center: cell line control, YAP1 IP-MS from YAP1 KO cells and right: aB control, non-specific control antibody IP-MS from HEK293). **B.** Distribution of coefficient of variation (CV) values of proteins identified in the indicated purifications. **C.** Number of proteins identified in the indicated purifications. **D/F.** Volcano Plot generated from YAP1 endogenous immuno-affinity purification using two controls (YAP1 KO **D**; non specific aB **F**). Protein identified and filtered as interactor (SP>0.9) in fractionated (light blue) and not fractionated (blue) AP-MS from HEK293 cells expressing epitope tagged YAP1 are annotated. **E/G.** Receiver-operating characteristic (ROC) curve and corresponding area under the curve (AUC) showing performance of YAP1 immune-affinity purification (using YAP1 KO cell line (**E**) and non specific aB (**G**) controls) as benchmarked against interactors identified with AP-MS of Strep-HA YAP1 ectopically expressed (blue line) and BioGRID annotated interactors (red line).


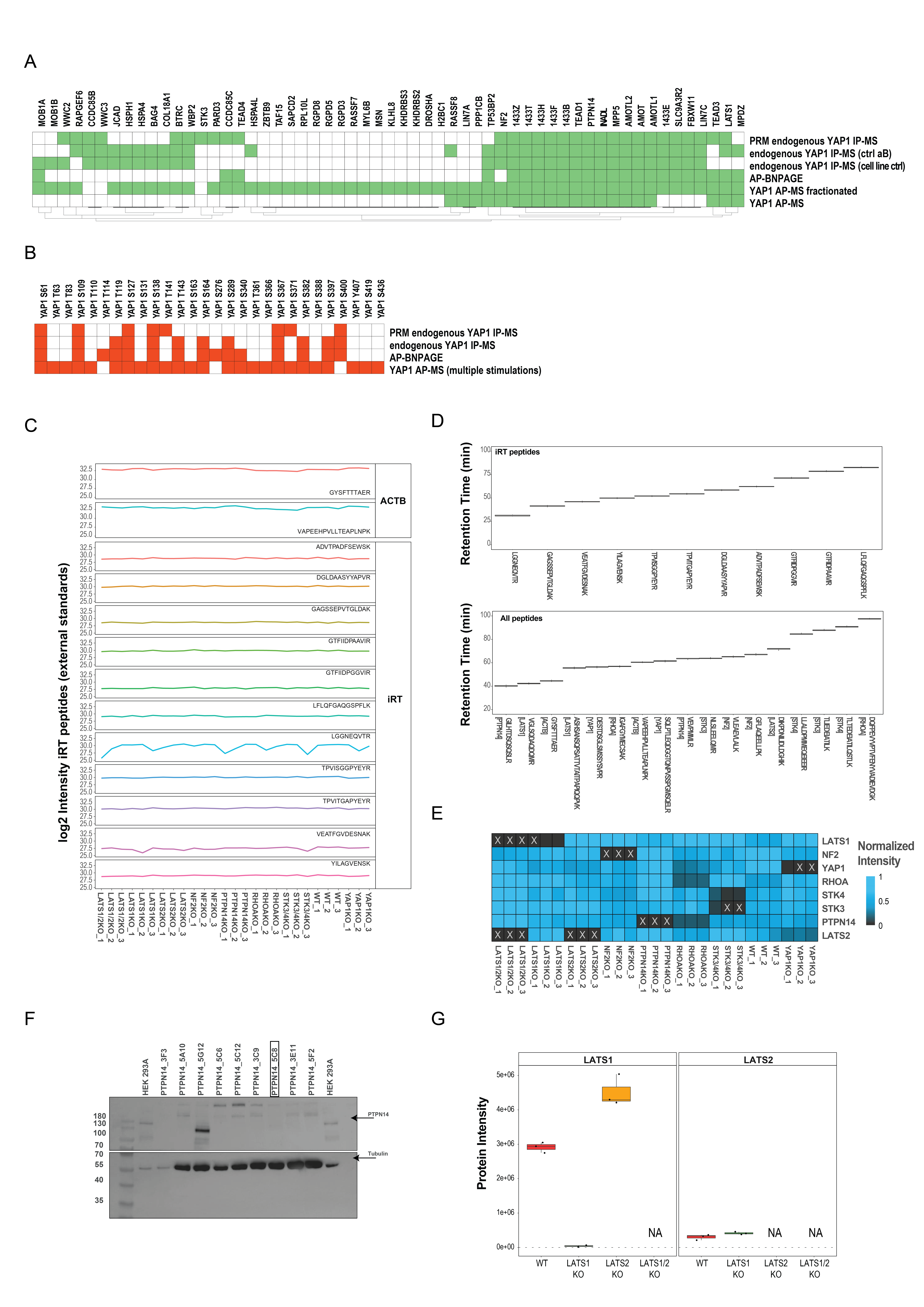


**Appendix Figure S8** Targeted proteomic quantification and characterization of genetic deletion in nine different cell lines. **A.** Heatmap depicts all proteins monitored (identified and quantified with different approaches) across indicated experimental setups used in this study. **B.** Heatmap depicting all YAP1 phosphosites (identified and quantified with different approaches) in all different experiments setup used in this study. **C.** Quantitative values of external standard (iRT peptides) spiked in the measurement for the targeted proteomic characterization of genetic deletions. Two peptides from Actin are used as loading control to normalize the injected lysate amount. Quantitative values are obtained from the sum of the transition values. **D.** Retention time of iRT (top) and selected peptides (bottom) used to evaluate genetic deletion efficiency. **E.** Characterization of genetic deletions in nine different cell lines. The heatmap reports the mean value of proteins intensities from three independent biological replicates normalized for the maximum detected value. **F.** Validation of PTPN14 deletion cell lines. Expression levels of PTPN14 in parental HEK293A control cell lines and CRISPR/Cas9 engineered PTPN14 KO clones in HEK293A cell lines as measured by Western blotting with the indicated antibodies. Clone PTPN14 5C8 has been selected for further analysis. **G.** Protein intensity abundance from three independent biological replicates of LATS1 (left) and LATS2 (right) in the indicated KO cell lines.


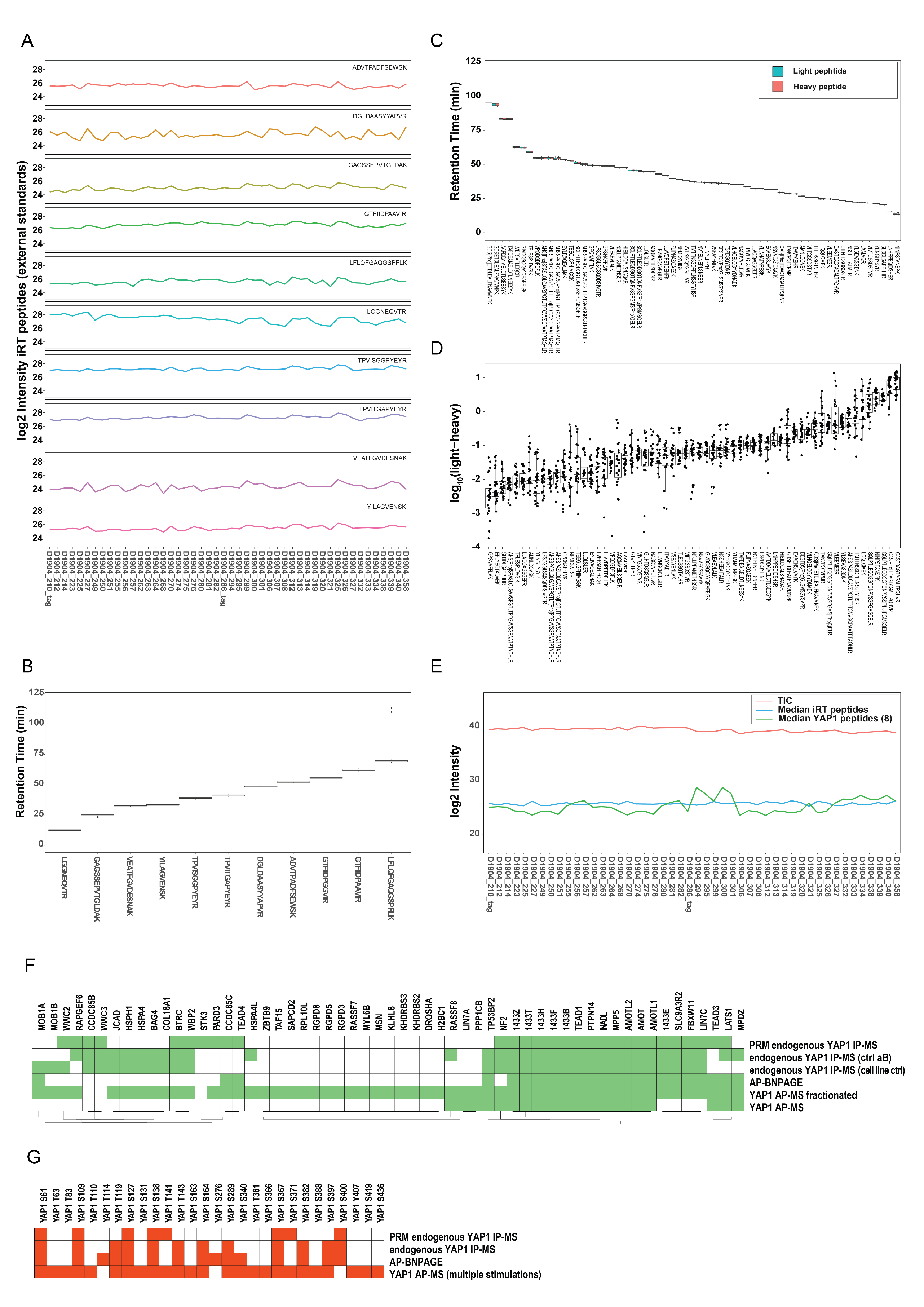


**Appendix Figure S9** Targeted proteomic profiling of endogenous YAP1 phosphopeptides and interactors in cell lacking Hippo pathway members **A.** Intensities of external peptide standard (iRT peptides) spiked in the measurement for the targeted proteomic profile of YAP1 phosphopeptides and interactors in a panel of seven cell lines with Hippo genetic deletions. Quantitative values are obtained from the sum of the transition values. **B.** Retention time of iRT peptides. **C.** Retention time of monitored endogenous and reference peptides (light and heavy) for YAP1 interactors and phosphosites. **D.** Normalized intensity of monitored peptides expressed as the log10(light-heavy) peptide. All peptides are normalized using spiked in corresponding heavy reference peptides. **E.** Data are normalized based on TIC (Total Ion Current), median intensity of iRT peptides and mean intensity of YAP1 non phosphorylated peptides (8). All values used for the normalization are reported in the plot.


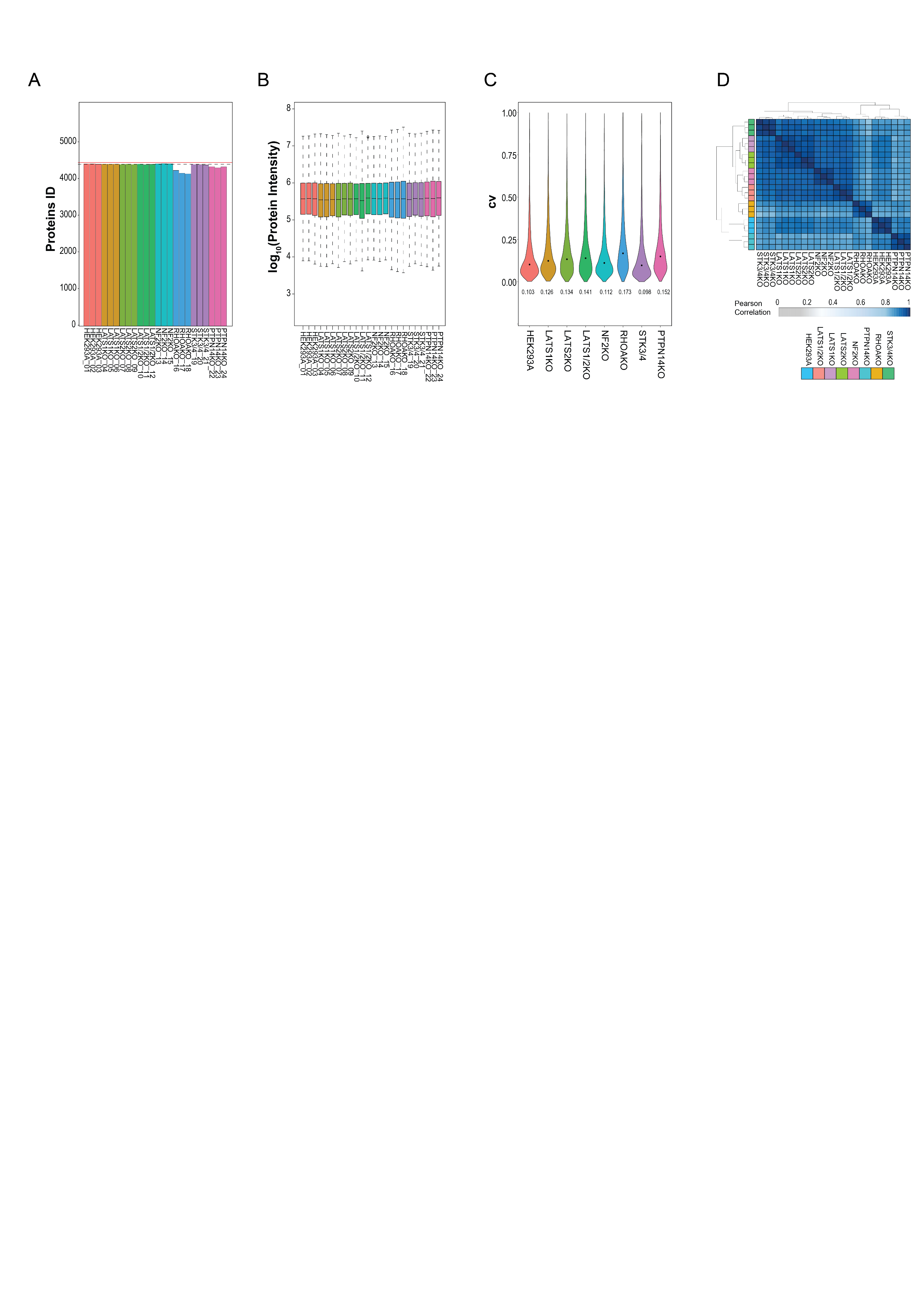


**Appendix Figure S10.** Differential protein expression data as determined by DIA proteomic workflow. **A.** Number of identified proteins in the DIA dataset. **B.** Distribution of protein intensity (log10) in the DIA dataset. **C.** Distribution of coefficient of variation (CV) values. **D.** Correlation matrix of protein intensities across indicated genetic backgrounds.


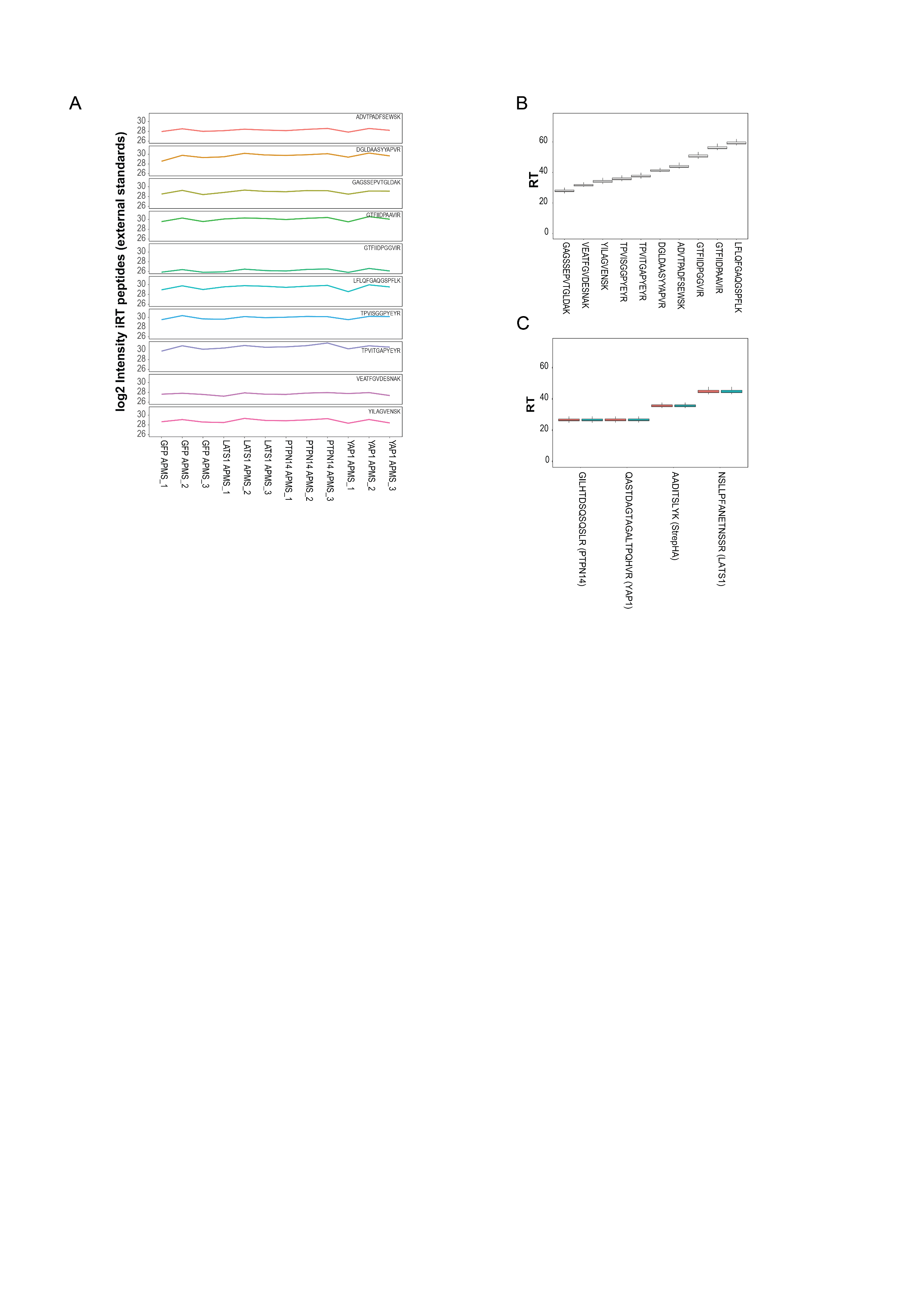


**Appendix Figure S11** Targeted proteomic quantification of PTPN14, LATS1 and YAP1 in reciprocal AP-MS. **A.** Intensities of external peptide standard (iRT peptides) spiked in the measurement across different APMS experiments. Quantitative values are obtained from the sum of the transition values. Retention time of iRT peptides (**B**) and monitored peptides (**C**) used in the AP-MS experiment.
