# Supplementary material for: Phosphorylation‐linked complex profiling identifies assemblies required for Hippo signal integration
Source: Mol Syst Biol. 2023 Mar 10;19(4):e11024. doi: 10.15252/msb.202211024 (PMC10090947; doi:10.15252/msb.202211024)
Supplement: Supplementary file 2 — Expanded View Figures PDF [file MSB-19-e11024-s004.pdf]

Expanded View Figures

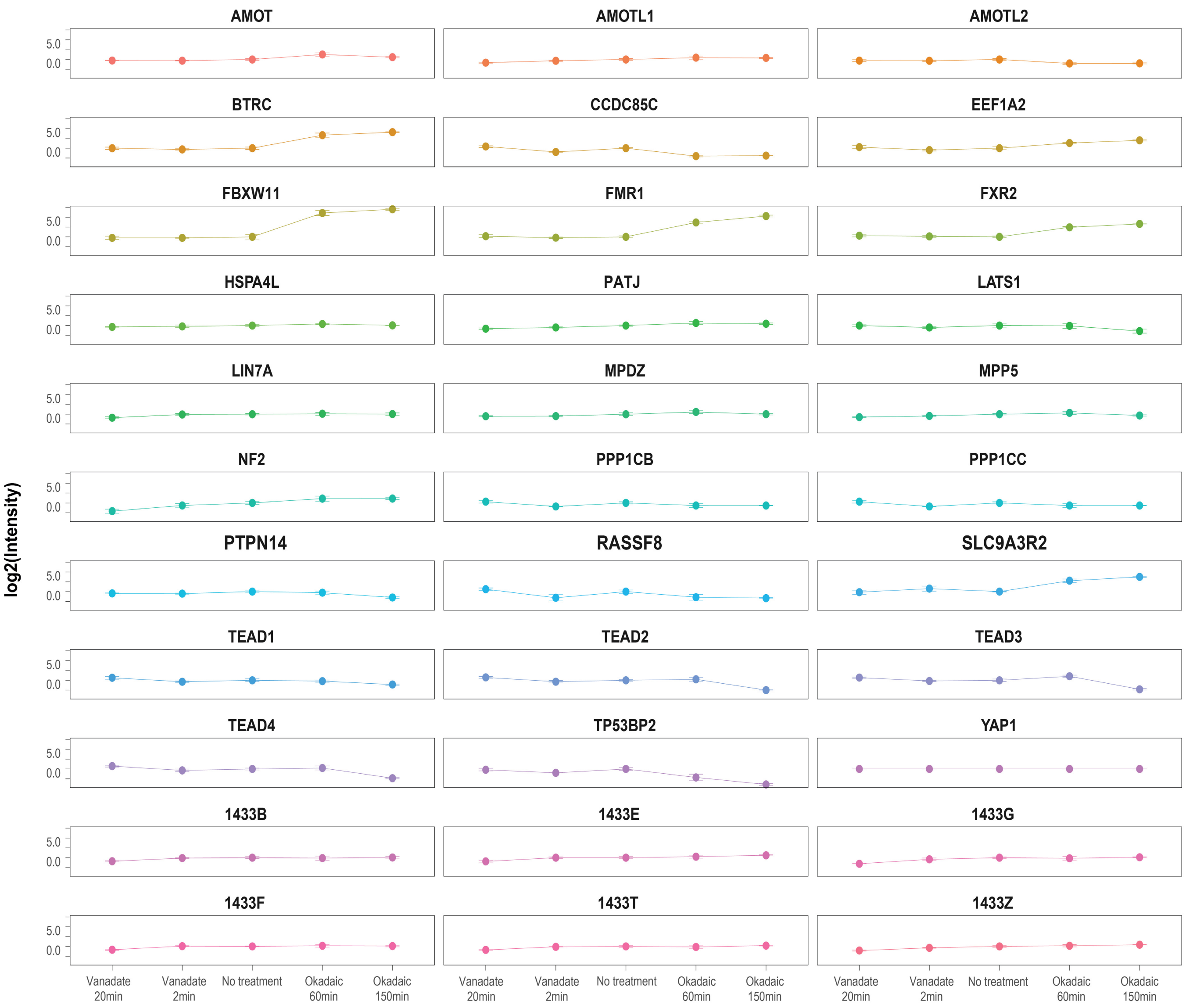

Figure EV1. Plasticity of YAP1 interactors.

Kinetic profiles of Strep-HA YAP1 interactors identified with SAINT SP score > 0.9 in at least one condition. Mean values of MS1 intensity from three independent replicates and SE error are reported for the following conditions (No treatment, Vanadate treatment for 2 and 20 min and Okadaic acid treatment for 60 and 150 min).

© 2023 The Authors

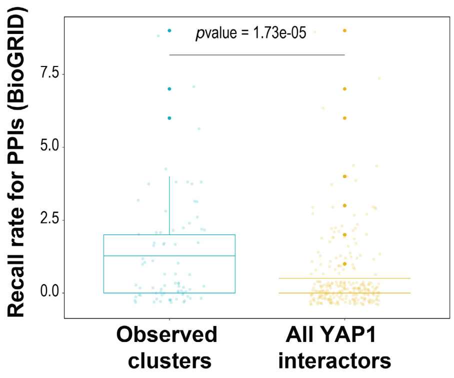

Figure EV2. Recall rate for Protein–Protein Interacion pairs in co-migrating clusters is higher than all pairs of YAP1 interactors.

Recall rate (BioGRID) for Protein–Protein Interaction pairs in co-migrating cluster compared to all interactions pairs of YAP1 interactors. Significance is evaluated by two-side unpaired *t*-test assuming normal distribution. The boundaries of the box plot correspond to the quantiles Q1 (25%) and Q3 (75%). Lower and upper whiskers are defined by Q1− 1.5IQR and Q3+ 1.5IQR.

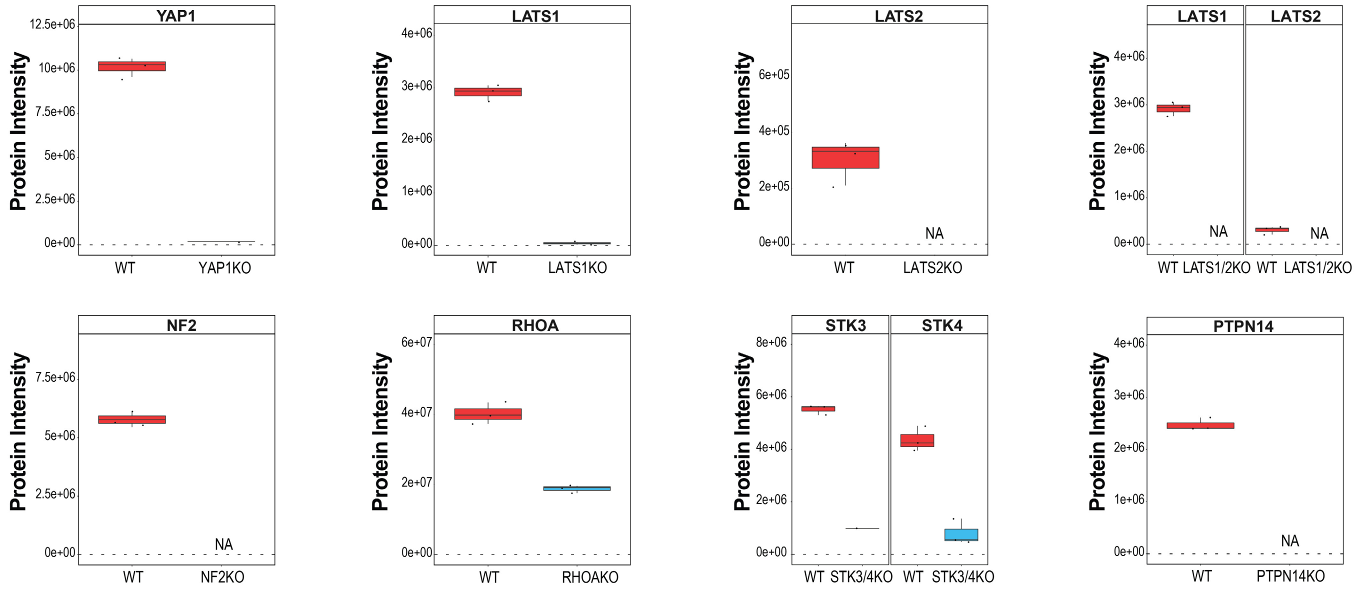

Figure EV3. Validation of KO cell lines.

Targeted proteomic quantification and characterization of genetic deletion in nine different cell lines. The boxplot reports the intensities of the indicated proteins in the parental HEK293A control cell line (left, red) and the respective KO cell line (right, blue). Data shown in the plot are generated from three independent biological replicates. The boundaries of the box plot correspond to the quantiles Q1 (25%) and Q3 (75%).

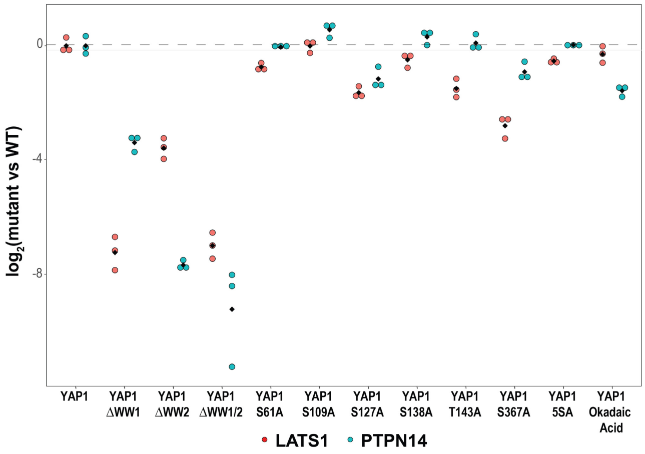

Figure EV4. Effect of YAP1 mutants on YAP1 interactions with PTPN14 and LATS1.

Intensity profile of YAP1 interactors in a panel of YAP1 mutants. The panel of mutants is composed by Strep-HA YAP1 phospho mutants (six single mutants and one with multiple mutations S5A) and WW domain mutants (ΔWW1, ΔW-W2, and ΔWW1/2). Interactors are identified and quantified by MS1 intensity after streptavidin affinity purification of YAP1. LATS1 (red) and PTPN14 (cyan) MS1 intensities of three independent biological replicates are expressed as log2 fold change compared with YAP1 wild-type control.

© 2023 The Authors
