# Supplementary material for: Phosphorylation‐linked complex profiling identifies assemblies required for Hippo signal integration
Source: Mol Syst Biol. 2023 Mar 10;19(4):e11024. doi: 10.15252/msb.202211024 (PMC10090947; doi:10.15252/msb.202211024)
Supplement: Supplementary file 14 [file MSB-19-e11024-s012.pdf]

molecular systems biology

Phosphorylation-linked complex profiling identified assemblies required for Hippo signal integration

Federico Uliana, Rodolfo Ciuffa, Ranjan Mishra, Andrea Fossati, Fabian Frommelt, Sabrina Keller, Martin Mehnert, Eivind Birkeland, Frank van Drogen, Nevena Srejic, Matthias Peter, Nicolas Tapon, Ruedi Aebersold, and Matthias Gstaiger
DOI: 10.15252/msb.202211024

Corresponding author(s): Matthias Gstaiger (matthias.gstaiger@imsb.biol.ethz.ch), Federico Uliana (federico.uliana@bc.biol.ethz.ch)

Review Timeline:

Submission Date:	16th Mar 22
Editorial Decision:	10th May 22
Revision Received:	22nd Jan 23
Editorial Decision:	20th Feb 23
Revision Received:	21st Feb 23
Accepted:	22nd Feb 23

Editor: Jingyi Hou

Transaction Report:

(Note: With the exception of the correction of typographical or spelling errors that could be a source of ambiguity, letters and reports are not edited. Depending on transfer agreements, referee reports obtained elsewhere may or may not be included in this compilation. Referee reports are anonymous unless the Referee chooses to sign their reports.)

Thank you for submitting your work to Molecular Systems Biology. We have now heard back from two of the three reviewers who agreed to evaluate your study. Unfortunately, after a series of reminders, we did not manage to obtain a report from Reviewer #3. In the interest of time, I prefer to make a decision now rather than further delay the process. If we receive the comments from Reviewer #3, we will send them to you, and you can address the issues raised by Reviewer #3 together with those raised by the other two reviewers. You will see from the comments below that Reviewers #1 and #2 find the topic of the study of interest. However, they raise substantial concerns about your work, which should be convincingly addressed in a major revision of the present manuscript.

Without reiterating all the points listed below, the most fundamental issues that need to be convincingly addressed are the following:

- Both Reviewers #1 and #2 mentioned that it would be essential to provide additional experimental validations to better support several conclusions of the manuscript.
- Reviewer #2 (an expert in YAP1 biology) pointed out that the application of the presented proteomics workflow remained relatively preliminary and thought some follow-up experiments would be required to provide novel mechanistic and biological insights, especially regarding the role of PTPN14 in the Hippo pathway. During our pre-decision cross-commenting process (in which the reviewers are given a chance to make additional comments, including on each other's reports), Reviewer #1(a proteomics expert) agreed with Reviewer #2's points and thought they should be addressed before publication. I have also included these comments below after the reviewers' reports.

All other issues raised by the reviewers need to be satisfactorily addressed as well. As you may already know, our editorial policy allows in principle a single round of major revision, and it is therefore essential to provide responses to the reviewers' comments that are as complete as possible.

Reviewer #1:

Phosphorylation linked complex profiling identified complexes required for Hippo signal integration

Review letter
YAP1 is a widely-studied signal integrator protein ranking among the top proteins with the most known phosphorylation sites and protein-protein interaction partners. Uliana et Ciuffa et al., present an interesting combination of techniques to study the relationship between YAP1 phosphorylation and YAP1-associated protein complexes. Overall the study is well done and logically executed.
However, explanation and presentation of the techniques used and results obtained should be clarified substantially. Furthermore, some of the functional conclusions drawn rely solely on correlational data and thus would benefit greatly from additional experimental validation.

Major comments:

1. Figures and text need to be clarified with respect to what type of experiment is being described and what type of data is being collected. For instance, the figures are often unclear whether the experiment consists of pulling down ectopic expression of tagged proteins or endogenous protein. This information should also be clearly in the figures, for instance in the small cartoons describing each approach. Also the affinity tag used (Strep-HA?) is not clearly described. As the authors present the study as a "general method", methodological details such as this need to be in the forefront of the presentation throughout.
2. The generality of the method is somewhat questionable, as much of the biologically interesting data is generated using bespoke knockout cell lines based on extensive pre-existing knowledge of YAP1 biology. This should be discussed or distinguished more clearly.
3. Only the phosphorylation state of YAP1 is considered in the study. However, it seems equally likely that the phosphorylation state of the interaction partners will have an impact on the resulting PPIs and complexes. Were no phosphosites detected on the interactors?
4. Figure 3 panel b is a key part of the manuscript, but it's current presentation is confusing. See below for details.
5. The use of spectral counting (as part of the SAINT algorithm) does not represent the state of the art of MS data analysis anymore. Reanalysis with intensity-based analyses should be preferred to be up to date with current analysis standards.
6. The study aims to establish the causal links between YAP1 phosphorylation and protein-protein interactions. Once sites of interest have been identified, mutagenesis remains the method of choice to test site-specific hypotheses. Given that the method relies on using ectopic expression of the protein of interest, targeted mutagenesis of phosphorylation sites should be feasible and could be incorporated into the study. This would significantly strengthen the findings and highlight the power of the method.

Detailed comments:
Text:
1. The study is framed as studying signaling, despite the fact that all measurements done rely on steady-state systems (with the exception of the phosphatase inhibition experiment used to generate the library of potential phosphosites) without controlled stimulation of signaling.
2. Typo introduction: "protein-protein" lowercase
3. Verb missing in sentence: "protein-protein interactions (PPIs) and protein phosphorylation with high throughput, dynamic range, accuracy and sensitivity3."
4. Typo: "are dealt with in distinct experimental and"
5. "Defining dependencies between phosphosites and specific interactions is limited by several technical and conceptual factors. First, the consistent and quantitative detection of phosphosites is limited by their low abundance and difficulties associated with the correct localization of the phosphate ester groups to specific amino acid residues10,11. Second, PPI data generated by AP-MS or proximity labeling of a bait protein indicate the identity of interacting or proximal proteins in the tested cellular context. Nevertheless, these methods fail to probe the actual composition of specific protein complexes as a function of the cell's signaling state or to resolve the association of (phosphorylation) proteoforms with specific complexes12."
a. Structure of text does not make proper sense
6. " with spatially resolved determination of PPIs."
a. What spatial dimension is this referring to?
7. " Both signaling hubs and"
a. Are signaling hubs always linked to many PPIs? Or what is being referred to here?
8. " context-specific interdependencies"
a. What context is being referred to?
9. " only 4 of 52 known and annotated sites15."
a. That a site appears on phosphositeplus does not make it "annotated". A large proportion of the sites do not have any studies looking at them in detail.
10. "protein phosphorylation databases"
a. Is this referring again to phosphosite plus? The wording is confusing, as it sounds like other databases are referred to
11. Grammar/typo: "we establish generic method"
12. "Figure S2a/b/c/d/e/f/g/h/i"

a. Unnecessary reference to all subpanels. Use either "a-i" or leave it out, where the entire figure is referred to. Same for later on "Figure S3, S4, S5a/b/c/d/e/f"
13. "SH-tag"
a. Explain explicitly, what the tag is at first mention
14. " differentially affected the direction and magnitude of YAP1 phosphorylation"
a. Direction of phosphorylation?
15. " unbiased map of YAP1 phosphosites"
a. The map is based on artificial increase in phosphorylation, so not completely unbiased
16. " represents binary interactions"
a. Why only binary?
17. "deletion of RHOA (although not quantitative), which"
a. The deletion was partial, but not complete
18. Discussion: "to each of the 9 identified complexes."
a. The authors have been careful to refer to the identified co-isolating groups as "modules". This should be also carried through the discussion.
19. "Our targeted proteomics approach revealed a distinct pattern of hyperphosphorylated YAP1 sites"
a. This should be "hypophosphorylated", right?
20. "a key player in enabling the cell density-dependent YAP1 interactome reshaping and signaling."
a. Is density-dependent signaling actually broken in PTPN14 KO cells?

Figures:
1. Figure 1:
a. It looks like the main aim in Figure 1 is to generate a library of potential phosphosites for the protein investigated. This should be clearly stated.
b. Figure 1 should also be clearer on, what the data gathered from this experiment is.
c. Is there risk of measuring non-physiological phosphorylation under the strong phosphatase inhibition used?

2. Figure 2:
a. Panel d: number of replicates and significance estimates should be indicated
b. Panel e: data variability is difficult to estimate in the current linegraphs. Replicates or standard deviations should be shown.

i. INADL is not shown in the figure, nor discussed, despite it being the top interactor in Figure 4B. Why is this?
c. Panel f: this panel would greatly benefit from a title
3. Figure 3:
a. Here, again, it is unclear, whether experiment uses ectopic expression or endogenous protein.
b. Also, it is unclear, whether this experiment is targeted or untargeted MS. The text and figure legends seem to contradict each other at times.
c. Panel b: This panel is one of the most important in the manuscript and represents a nicely-done and interesting experiment. However, the presentation is partially confusing and could be misleading.
i. The heatmap presents a mixture of peptide-level information (YAP1 phosphorylated peptides) and protein groups. Furthermore, in its current representation the heatmap looks like it is representing raw data, which is in fact not the case. Rather, the heatmap shows what are essentially devoncoluted chromatograms. This needs to be clearly indicated already in the main figure.
ii. Color scale should be changed (red/green) to a more readable and color-blind friendly. E.g. red/white/blue?
d. Panel d: Should more clearly mention that the sum intensity over fractions is shown
e. Panel e: The use of 1000 randomly-picked configurations from a relatively small subpool of proteins is arbitrary and runs the risk of inflating significance in the statistical comparison.
f. The explanation of what was done is also unclear.
g. What do the individual points represent? Are they the individual clusters? Are all clusters here? Only 7 points are visible, although 9 clusters were identified The points should also be larger to make them more visible.
h. The 1433 protein module (module 9) should be mentioned and discussed in the text. Do these represent disassembled complexes?
4. Figure 4
a. Not clear, what the controls are. aB control in particular is not sufficiently explained. The text says: "using the flow-through of the antibody purification as non-specific antibody (aB control)". However, the figure legend says: "aB control with non-specific control antibody IP-MS from HEK293A." M&M is also unclear on this.
b. Panel b:
i. Not significant interactor should say $p>0.05$
ii. The cell line ctrl is YAP1 KO cells and should be mentioned explicitly.
iii. Why is INADL not followed up or mentioned?
c. Panel c
i. While the use of different types of plots is laudible, in this case this representation is counterintuitive representation and/or insufficiently explained. For instance, what does the line thickness represent? A simple heatmap or the like would be preferrable and more accessible to most readers.
d. Panel d
i. The axis title "l/h" is unclear and not sufficiently explained. A more descriptive title would be recommended.
e. Panel f
i. Do the individual points represent replicates?
ii. Furthermore, it is unclear, whether the target proteins of the KO cells were removed from the data before doing the PCA.
5. Figure 5
a. Panel b
i. What is "[-]"
b. Panel d
i. The authors could highlight proteins of interest here, e.g. LATS1 and CYR61
c. Panel e
i. Also here could highlight, e.g., PTPN14 and CYR61?
d. Panel f
i. It should be clarified more clearly in the figure that/if this represents whole proteome data
e. Panel g
i. Would the second PTPN14 peak suggest that there are other factors involved in recruiting LATS1 to YAP1 as well? Since there is a second peak with PTPN14 but without LATS1. Could this be due to differentially phosphorylated PTPN14?
6. Figure S3 and S4
a. X axis missing
7. Figure S5
a. iRT peptides should be explained more clearly
8. Figure S6
a. Panel e
i. "identified exogenous interactor?"
9. Figure S8
a. Panel c
i. Typo: "light petide"
10. Figure S10
a. Panel c
i. Typo: "()LATS1"

Additionally:
The finding that PTPN14 augments inhibition of YAP1 by LATS1/2 is interesting. Although potentially outside the scope of this study, the authors might want to consider doing rescue experiments with different PTPN14 mutants (e.g. catalytically dead) in the PTPN14 KO cell line to better understand the role of PTPN14 in the process.

Reviewer #2:

In the manuscript entitled "Phosphorylation linked complex profiling identified complexes required for Hippo signal integration", Uliana et al. used proteomics as an approach to investigate phosphorylation and interactome of YAP, a key effector protein in the Hippo pathway. In particular, they refined YAP-interacting proteins using a native PAGE system and revealed different YAP-associated protein complexes. Moreover, the authors compared YAP phosphorylation and interactome in a group of Hippo components-deficient cells, where they underscored the role of PTPN14, a previously known Hippo pathway regulator, in mediating LATS-induced YAP phosphorylation and inhibition. Overall, this study provides a new proteomic strategy for characterizing the relationship between protein phosphorylation and interactome for a protein of interest. However, the reviewer feels that applying this proteomic flow to analyzing YAP phosphorylation and interacting proteins was superficial, lacking experimental validation and mechanistic insights in advancing the current understanding of YAP regulation.

Specifically,

1. The novelty of the YAP-related interactome analysis presented here is low. First, the Hippo-related proteomic studies have been conducted by different labs a few years ago, who reported almost same interacting proteins for YAP as shown by the authors here. Second, among these published studies, similar phosphatase inhibitor treatment has been used to compare the Hippo PPI change, including the one for YAP (PMID: 24255178). Third, PTPN14 has already been known as a negative regulator of YAP by restricting YAP nuclear localization and promoting LATS activity (PMID: 25023289; PMID: 23613971; PMID: 22525271; PMID: 22948661).

2. In Fig.2, profiling YAP phosphorylation sites was descriptive, lacking experimental validation and further mechanistic investigation. In addition to the known sites, what are the roles of the rest phosphorylation sites in regulating YAP? What kinases are required to phosphorylate these sites? Why would phosphorylation of these sites regulate the interaction between YAP and its binding partners (Fig. 2e)?

3. Revealing different YAP-associated protein complexes by native PAGE is of interest (Fig. 3). Again, experimental validation of these distinct YAP-associated protein complexes is missing. In addition, how YAP phosphorylation affects these complex formation or switch (Fig. 3c) is needed to be addressed.

4. The data supporting current working model (Fig. 5i) were quite preliminary. Can PTPN14 directly bind LATS? Where is this PTPN14/LATS/ YAP complex localized in cells? Will the interaction between YAP and LATS be reduced in the PTPN14 KO cells as compared to that in wild-type cells? Since both LATS and PTPN14 contain PPxY motif that is required for their association with YAP (through YAP WW domain) (PMID: 18158288; PMID: 23613971; PMID: 22525271; PMID: 22948661), will PTPN14 and LATS compete each other for YAP binding? How is the PTPN14/LATS/YAP complex regulated in response to Hippo upstream signaling events (e.g., serum starvation, cell density, actin disruption)?

****
Pre-decision Cross-commenting:

Reviewer #1
We agree with the points raised by ref 2 and think they should be addressed before publishing in MSB.

Reviewer #2
I agree with Reviewer #1's comments and feel they are constructive and in details.
Actually, many previous studies have performed mass spec analyses of YAP and also characterized PTPN14 in Hippo pathway regulation. To move the field forward, if the authors decide to functionally validate their new mass spec findings and elucidate the newly discovered role of PTPN14 in the Hippo pathway as suggested, I am ok with a major revision decision.

Response to the referees:

Phosphorylation-linked complex profiling identified assemblies required for Hippo signal integration

Federico Uliana[1,2#*], Rodolfo Ciuffa[1#], Ranjan Mishra[2], Andrea Fossati[1,3,4,5], Fabian Frommelt[1], Sabrina Keller[1], Martin Mehnert[1], Eivind Salmorin Birkeland[2], Frank van Drogen[2], Nevena Srejic[2], Matthias Peter[2], Nicolas Tapon[6], Ruedi Aebersold[1], Matthias Gstaiger[1*]

1 Department of Biology, Institute of Molecular Systems Biology, ETH Zurich, Zurich, Switzerland

2 Department of Biology, Institute of Biochemistry, ETH Zurich, Zurich, Switzerland

3 University of California San Francisco, Quantitative Biosciences Institute (QBI), San Francisco, CA, 94158, USA

4 University of California San Francisco, Department of Cellular and Molecular Pharmacology, San Francisco, CA, 94158, USA

5 J. David Gladstone Institutes, San Francisco, CA 94158, USA

6 Apoptosis and Proliferation Control Laboratory, The Francis Crick Institute, 1 Midland Road, London, NW1 1AT, UK

* Correspondence: Correspondence to Matthias Gstaiger and to Federico Uliana

[#] Contributed equally

Reviewer #1:

Phosphorylation linked complex profiling identified complexes required for Hippo signal integration

Review letter
YAP1 is a widely-studied signal integrator protein ranking among the top proteins with the most known phosphorylation sites and protein-protein interaction partners. Uliana et Ciuffa et al., present an interesting combination of techniques to study the relationship between YAP1 phosphorylation and YAP1-associated protein complexes. Overall the study is well done and logically executed. However, explanation and presentation of the techniques used and results obtained should be clarified substantially. Furthermore, some of the functional conclusions drawn rely solely on correlational data and thus would benefit greatly from additional experimental validation.

Major comments:

1. Figures and text need to be clarified with respect to what type of experiment is being described and what type of data is being collected. For instance, the figures are often unclear whether the experiment consists of pulling down ectopic expression of tagged proteins or endogenous protein. This information should also be clearly in the figures, for instance in the small cartoons describing each approach. Also the affinity tag used (Strep-HA?) is not clearly described. As the authors present the study as a "general method", methodological details such as this need to be in the forefront of the presentation throughout.

We thank the reviewer for this comment. We substantially modified Figure 1 to illustrate in greater detail the workflow indicating the figures corresponding to each of the steps. In addition, we have changed Figure legends and the Figure 2a, 3a and 4a to specify, whenever needed, whether we have used ectopically or endogenously expressed YAP1.

2. The generality of the method is somewhat questionable, as much of the biologically interesting data is generated using bespoke knockout cell lines based on extensive pre-existing knowledge of YAP1 biology. This should be discussed or distinguished more clearly.

YAP1 has been specifically chosen as an example since it is known to be regulated by multiple phosphorylation sites and because of the known complexity of its interactome. The pre-existing knowledge of YAP1 was of advantage to benchmark our method and ensure that besides uncovering new findings it can also reproduce previous insights. We believe that mapping of phosphorylation sites by phosphatase inhibition, determining correlation between interactors and phosphosites by BNPAGE, and defining the regulatory role of phosphosites by deletion mutants/pulldowns will be generally useful for signaling proteins that similar to YAP1 are known to undergo multisite-phosphorylation and complex formation with many other cellular proteins. Although we think that the method can be applied in principle to all proteins, we agree with the reviewer though that the approach may not be as useful and informative for proteins that are poorly phosphorylated and with very few interactions. We emphasized this point in our discussion by including the sentence:

"In principle AP-BNPAGE profiling can be applied to a broad range proteins, but may be particularly informative for proteins that, similar to YAP1, are subject to multisite modifications with a rich interactome under steady state and upon signaling.."

3. Only the phosphorylation state of YAP1 is considered in the study. However, it seems equally likely that the phosphorylation state of the interaction partners will have an impact on the resulting PPIs and complexes. Were no phosphosites detected on the interactors?

This is an interesting point, and we have attempted, over the course of this study, to take interactors' phosphorylation status into consideration. However, because of low peptide coverage and low signal-to-noise ratio, we found that consistent identification, localization and reliable quantification of phosphopeptides of interactors across the experiments turned out to be very challenging.

To provide an idea on the sparseness of the YAP1 interactors phosphorylation dataset, we present in the heatmap below (**FFR1**) the phosphosites that we have identified for the indicated interactors (x axis) across three different experiments: pull-down of endogenously (IP-MS), ectopically expressed YAP1 (AP-MS Strep HA), as well as AP-BNPAGE. For each experiment, data in dark orange shows phosphosites identified in triplicates with at least a localization score higher than 0.8. Overall, we identified a total of 89 phosphosites across 14 interactors (especially AMOT proteins), only 35 of which are confidently identified, and only 9 are consistent at least across two experiments (less than 10% of all interactor phosphosites).

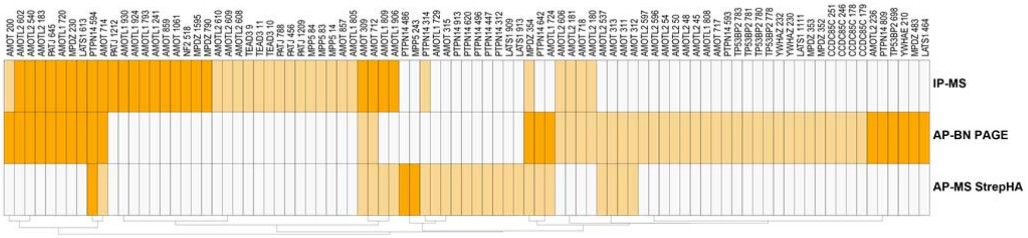

FFR1. Heatmap shows identified phosphosites for YAP1 interactors across all acquired datasets (light orange) and filtered phosphosites (orange) identified robustly across three replicates with a localization score higher than 0.8.

4. Figure 3 panel b is a key part of the manuscript, but it's current presentation is confusing. See below for details.

In the "Detailed comments" part we have addressed these reviewer's comments.

5. The use of spectral counting (as part of the SAINT algorithm) does not represent the state of the art of MS data analysis anymore. Reanalysis with intensity-based analyses should be preferred to be up to date with current analysis standards.

In the manuscript we have been using spectral count information exclusively for filtering specific interactors via SAINT similar to most other recent AP-MS studies[1]. We re-analyzed the YAP1 AP-MS experiments based on MS1 signal, as suggested by the reviewer. The results are presented below as a volcano plot (**FFR2**). Labeled proteins indicates the interactome defined based on spectral counting (Saint probability score higher than 0.9 in at least one condition). Results are consistent, in that the most enriched/significant proteins in the volcano plots are also part of the interactome we have defined with spectra counts. Nevertheless, we deliberately decided to filter our data on spectral counting as the SAINT Score provides a threshold connected to a probabilistic model for filtering high confident interactors (BFDR< 0.05) and dispenses us from the need of using an arbitrary log2 enrichment threshold. Only identification of high confidence YAP1 interactors was performed using spectra count and SAINT (Figure 2E), whereas all other experiments (including the kinetic profile of interactors in Figure 2E) were performed using quantification approaches based on MS1 peptide area or targeted MS.

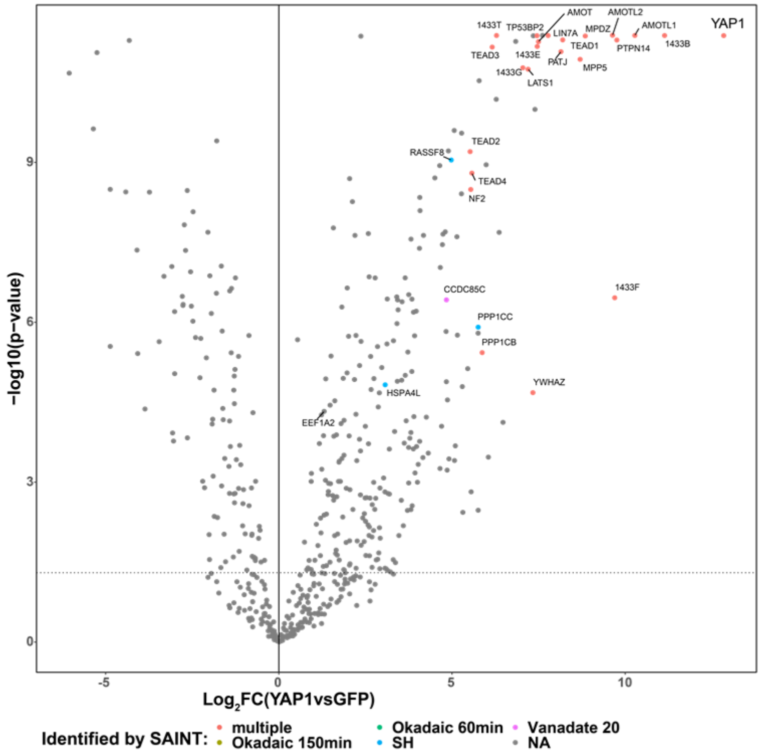

FFR2. Volcano plot shows proteins enriched in YAP1 Strep-HA pulldown. Data are generated using MS1 signal for quantification. Proteins filtered as interactors from SAINT analysis (SP>0.9) are highlighted. Different color code indicates in which condition the protein was identified as an interactor of YAP1.

6. The study aims to establish the causal links between YAP1 phosphorylation and protein-protein interactions. Once sites of interest have been identified, mutagenesis remains the method of choice to test site-specific hypotheses. Given that the method relies on using ectopic expression of the protein of interest, targeted mutagenesis of phosphorylation sites should be feasible and could be incorporated into the study. This would significantly strengthen the findings and highlight the power of the method.

We agree and acknowledge the reviewer for the comment. In our revised version we addressed this point and performed new AP-MS experiments using six distinct YAP1 single-phosphosite mutants and one YAP1 multi-phosphosite mutant (5SA) as a bait. This allowed to infer a causal relationship between YAP1 phosphorylation and complex formation. For details please see also response 3 to reviewer 2. New Results are presented in Figure 3F-H and discussed in the text.

Detailed comments:
Text:
1. The study is framed as studying signaling, despite the fact that all measurements done rely on steady-state systems (with the exception of the phosphatase inhibition experiment used to generate the library of potential phosphosites) without controlled stimulation of signaling.

We agree with this comment and we added a sentence in the discussion underlining the importance of characterizing complex assembly/formation after stimulation:

"In principle AP-BNPAGE profiling can be applied to a broad range proteins, but may be particularly informative for proteins that, similar to YAP1, are subject to multisite modifications with a rich interactome under steady state and upon signaling. To measure signaling induced complex dynamics, AP-BNPAGE analysis across multiple conditions will greatly benefit from multiplexed MS data acquisition techniques[2]".

2. Typo introduction: "protein-protein" lowercase

We correct as suggested by the reviewer.

3. Verb missing in sentence: "protein-protein interactions (PPIs) and protein phosphorylation with high throughput, dynamic range, accuracy and sensitivity3."

We removed an erroneously introduced stop creating the referred-to nominal sentence. Now the sentence should read:

"Mass spectrometry represents the method of choice to analyze both Protein-Protein Interactions (PPIs) and protein phosphorylation with high throughput, dynamic range, accuracy and sensitivity[3–6]"

4. Typo: "are dealt with in distinct experimental and"

We correct the sentence.

"these two aspects are dealt with distinct experimental and computational settings,"

5. "Defining dependencies between phosphosites and specific interactions is limited by several technical and conceptual factors. First, the consistent and quantitative detection of phosphosites is limited by their low abundance and difficulties associated with the correct localization of the phosphate ester groups to specific amino acid residues10,11. Second, PPI data generated by AP-MS or proximity labeling of a bait protein indicate the identity of interacting or proximal proteins in the tested cellular context. Nevertheless, these methods fail to probe the actual composition of specific protein complexes as a function of the cell's signaling state or to resolve the association of (phosphorylation) proteoforms with specific complexes12."

a. Structure of text does not make proper sense

We reformulate and simplify the sentence:

"Affinity enrichment of a proteins of interest can partly alleviate sensitivity issues of global phosphoproteomics studies, however both AP-MS and proximity labeling provide a convoluted representation of interactions and phosphosites of concurrently purified complexes. Therefore, these methods fail to provide association between phospho-proteoforms and complex formation. Here we present an approach to separate and identify complex isoforms of a protein and its complex specific phosphosites".

6. " with spatially resolved determination of PPIs."
a. What spatial dimension is this referring to?

We removed 'spatially resolved', as we recognize it may be ambiguous (it can be both interpreted as referring to proximity labeling as well as cellular localization, the latter being what we initially intended).

7. " Both signaling hubs and"
a. Are signaling hubs always linked to many PPIs? Or what is being referred to here?

We refer to hub as protein partaking in many interactions; we replaced 'signaling' with 'interaction' to avoid implying that interacting with several proteins means necessarily being central in a given signaling pathway.

8. " context-specific interdependencies"
a. What context is being referred to?

By 'context' we refer here to specific cellular states (e.g. steady-state vs stimulated), but again we acknowledge that the term is somewhat potentially mis-interpretable and we removed it from the text, which now reads:

"For these reasons, we chose YAP1 in our study as a model to establish and apply a robust workflow to determine the interdependencies between phosphorylation and PPI formation (Appendix Figure S1A)."

9. " only 4 of 52 known and annotated sites15."
a. That a site appears on phosphositeplus does not make it "annotated". A large proportion of the sites do not have any studies looking at them in detail.

We amended the text as follows:

"Cell Signaling Technologies reports antibodies for only 4 of 52 sites catalogued in the Phosphositeplus repository".

10. "protein phosphorylation databases"
a. Is this referring again to phosphosite plus? The wording is confusing, as it sounds like other databases are referred to

We amended the text as follows:

"However, this protein phosphorylation database suggests a significant number of additional YAP1 phosphorylation sites…"

11. Grammar/typo: "we establish generic method"

We amended the text as follows:

"we establish a generic method"

12. "Figure S2a/b/c/d/e/f/g/h/i"
a. Unnecessary reference to all subpanels. Use either "a-i" or leave it out, where the entire figure is referred to. Same for later on "Figure S3, S4, S5a/b/c/d/e/f"

We modified the text as suggested by the reviewer.

13. "SH-tag"
a. Explain explicitly, what the tag is at first mention

We amended the text as follows:

"Specifically, we used YAP1, tagged with Strep-HA (SH), ectopically expressed in HEK293 cells under the control of a doxycycline-inducible promoter."

14. " differentially affected the direction and magnitude of YAP1 phosphorylation"
a. Direction of phosphorylation?

We removed 'direction'. Now the sentence reads:

"Okadaic acid and vanadate differentially affect the magnitude of YAP1 phosphorylation and, to a lesser extent, interactor association (Figure 2C)."

15. " unbiased map of YAP1 phosphosites"
a. The map is based on artificial increase in phosphorylation, so not completely unbiased

We replaced 'biased' with 'quantitative'.

16. " represents binary interactions"
a. Why only binary?

By binary interactions we mean that only pairs of bait-preys, as opposed to complexes with more than 2 members, can be inferred from AP-MS data. To clarify, we rephrased as follow:

"Since the AP-MS data result from concurrently purified YAP1 complexes"

17. "deletion of RHOA (although not quantitative), which"
a. The deletion was partial, but not complete

We replaced "*although not quantitative*" with "*only partial*".

18. Discussion: "to each of the 9 identified complexes."
a. The authors have been careful to refer to the identified co-isolating groups as "modules". This should be also carried through the discussion.

We replaced "*complexes*" with "*modules*" in the indicated instance.

19. "Our targeted proteomics approach revealed a distinct pattern of hyperphosphorylated YAP1 sites"
a. This should be "hypophosphorylated", right?

We thank the referee and we corrected the typo as correctly suggested by the reviewer.

20. "a key player in enabling the cell density-dependent YAP1 interactome reshaping and signaling."
a. Is density-dependent signaling actually broken in PTPN14 KO cells?

We acknowledge the reviewer for this comment, Wang et al.[2] reported that the density dependent translocation of YAP1 is regulated by PTPN14 and the knockdown of PTPN14 can activate YAP1 to increase cell proliferation. In the model proposed by *Wang et al*. a Cullin2 RING (E3 ubiquitin ligase) degrades PTPN14 at low cell density and leads to YAP1 nucleus translocation. The experiments presented in our manuscript will provide another important piece of information regarding the mechanism in which PTPN14 augmented the interaction between LATS1/2 and YAP1 which, in turn, leads to YAP1 inactivation.

Figures:
1. Figure 1:
a. It looks like the main aim in Figure 1 is to generate a library of potential phosphosites for the protein investigated. This should be clearly stated.

To address this, we modified Figure 1 and the following sentence as follows:

"Taken together, these data provide an extensive, quantitative map of YAP1 phosphosites/interactors and their responsiveness upon phosphatase inhibition, which serves as a reference library for subsequent experiments."

b. Figure 1 should also be clearer on, what the data gathered from this experiment is.

To improve the clarity of Figure 1 we have redesigned it to highlight the experimental setup, which shows the purification workflow and the corresponding figures in the manuscript.

c. Is there risk of measuring non-physiological phosphorylation under the strong phosphatase inhibition used?

We agree that phosphatase inhibition has the rysk to cause non-physiological levels of phosphorylation. It remains however an important tool to detect phosphorylation events that are otherwise hard to measure (because e.g. sub-stoichiometric or due to poor response factor of the corresponding peptide in the mass spectrometer), and also to define how broad the phosphorylation response/corresponding interaction changes can potentially be.

2. Figure 2:
a. Panel d: number of replicates and significance estimates should be indicated

The aim of panel 2D is to present YAP1 phosphorylation plasticity (over 25 phosphosites, only 15 are identified without treatment). In this analysis we considered phosphosites identified in all three replicates, therefore the matrix of phosphosites is rath sparse (Dataset EV1). The significance analysis would require a complete matrix and we would have to impute a large number of values, more than 33% of the entire dataset (123 over 365) with the risk of introducing artefacts. We preferred to report for Figure 2D the intensity of phosphosites identified in triplicates and the missing values (NA) in case this condition was not achieved.

b. Panel e: data variability is difficult to estimate in the current linegraphs. Replicates or standard deviations should be shown.

We supplemented the current plots with a line plot for each of the interactors reporting mean standard error (Figure EV1).

i. INADL is not shown in the figure, nor discussed, despite it being the top interactor in Figure 4B. Why is this?

This is due to a nomenclature inconsistency, which we now address consistently across the text. The new INADL name is PATJ.

c. Panel f: this panel would greatly benefit from a title

We added the following title to figure 2F: “*Correlation profiles between YAP1 S127 and 1433 /TEAD interactors”.*

3. Figure 3:
a. Here, again, it is unclear, whether experiment uses ectopic expression or endogenous protein.

We now highlighted the source of the protein in the Figure 3A with “Strep-HA Affinity purification”.

b. Also, it is unclear, whether this experiment is targeted or untargeted MS. The text and figure legends seem to contradict each other at times.

To clarify the acquisition strategy we added in the Figure 3a “DDA MS”.

c. Panel b: This panel is one of the most important in the manuscript and represents a nicely-done and interesting experiment. However, the presentation is partially confusing and could be misleading.

i. The heatmap presents a mixture of peptide-level information (YAP1 phosphorylated peptides) and protein groups. Furthermore, in its current representation the heatmap looks like it is representing raw data, which is in fact not the case. Rather, the heatmap shows what are essentially devoncoluted chromatograms. This needs to be clearly indicated already in the main figure.

To clarify Figure 3B we now distinguished protein profiles (based on protein groups) from peptide-level information (phosphopeptides) based on the font and color. We also stress in the legend that the represented data have been processed as described in the material and method, and are based on the raw profiles already reported in Appendix Figure S3 and S4.

ii. Color scale should be changed (red/green) to a more readable and color-blind friendly. E.g. red/white/blue?

We corrected and we changed Figure 3B with a scale from black to red with only positive Z score.

d. Panel d: Should more clearly mention that the sum intensity over fractions is shown

We change the figure legend of this panel (in the current version Appendix Figure S5D):

"Protein intensity correlation between YAP1 AP-MS and AP-BNPAGE (generated from the sum in all measured fractions of each protein intensity)"

e. Panel e: The use of 1000 randomly-picked configurations from a relatively small subpool of proteins is arbitrary and runs the risk of inflating significance in the statistical comparison.

We thank the reviewer for this important and relevant comment. We removed the comparison based on randomly picked configurations and replaced it with comparisons that should not introduce potentially artificial inflation of significance. Specifically, we compared the recall rate for all complex member pairs against all combination pairs of YAP1 interactors, we checked for normality (Shapiro test) and we calculated the significance with two-sided unpaired *t*-test (Figure EV2). Moreover, we calculated a simple GO enrichment for each complex and showed that, for most, proteins part of the same complex are significantly associated with specific compartments (Appendix Figure S5f). We think that this change would improve the readability of the manuscript.

f. The explanation of what was done is also unclear.

We reformulated the figures and the text as described above (see Reviewer 1, question related to Figure 3E):

"(i) Interactions between pairs of proteins belonging to the identified modules, have been reported in PPIs database significantly more often than all possible pairs of YAP1 interactors (Figure EV2)."

g. What do the individual points represent? Are they the individual clusters? Are all clusters here? Only 7 points are visible, although 9 clusters were identified The points should also be larger to make them more visible.

In the analysis performed in the previous version of the manuscript and not in this version (see Reviewer 1 question related to Figure 3E,F) we excluded two modules containing either a single protein and a phosphosite, or only phosphosites, and which are therefore not amenable to the analyses carried out.

h. The 1433 protein module (module 9) should be mentioned and discussed in the text. Do these represent disassembled complexes?

We think that the most plausible explanation is that 14-3-3 proteins disassembles during BNPAGE separation. However, we do not have orthogonal lines of evidences to corroborate this hypothesis. Another consideration for 14-3-3 behavior is related to the data analysis workflow. In this case the high abundant 14-3-3 proteins are normalized to 1 (like all the other proteins), this leads to a compression of the dynamic range and as consequence 14-3-3 proteins which are identified in fractions at higher molecular weight are not identified as peak and filtered out (the minimal intensity to be considered as peak is 40% of the main peak).

In the revised manuscript we described the limitations of AP-BNPAGE:

*"This approach, although successful in resolving the modular organization of YAP1 interactome, is limited by experimental and data analysis caveats that need to be emphasized. The experiment discussed required large amounts of cells (300*10^6 cells per replicate) and considerable MS acquisition time (nearly one week of measurements). In addition, the separation by BNPAGE may disrupt the most labile interactions, as described for the assembly intermediates identified in modules 5, 7 and 8, and in the case of 14-3-3 proteins, which co-migrate in a unique cluster at low molecular weight. Data analysis is challenged by co-migrating complexes that might lead to convoluted modules, although to a lesser extent than in total protein profiling experiments. Furthermore, under-sampling of low abundant phosphopeptides might compromise characterization of proteoforms and their assignment to a given module. "*

4. Figure 4
a. Not clear, what the controls are. aB control in particular is not sufficiently explained. The text says: "using the flow-through of the antibody purification as non-specific antibody (aB control)". However, the figure legend says: "aB control with non-specific control antibody IP-MS from HEK293A." M&M is also unclear on this.

In this experiment, aB control consists of non-specific antibodies which are obtained from the flow-through of YAP1 antibody purification. We rephrased the sentence in the main text and in the Figure 4B legend:

"To maximize the specificity of our purification, we used a double control strategy by using non-specific antibodies (aB control) and a YAP1 KO HEK293A line (cell line control) (see material and methods for details)."

"The experiment has been performed with anti C terminal peptide antibodies and with two controls: i) antibody control, IP-MS experiment using non-specific antibodies and ii) cell line control, IP-MS experiment using YAP1 specific antibodies in YAP1 KO cell line. "

b. Panel b:
i. Not significant interactor should say p>0.05

We correct Figure 4B as suggested by the reviewer.

ii. The cell line ctrl is YAP1 KO cells and should be mentioned explicitly.

In figure 4B we added in the x axis *"log2FC(YAP1 IP vs YAP1 KO)"*.

iii. Why is INADL not followed up or mentioned?

We changed the name to PATJ. (see point Figure 2e).

c. Panel c

i. While the use of different types of plots is laudible, in this case this representation is counterintuitive representation and/or insufficiently explained. For instance, what does the line thickness represent? A simple heatmap or the like would be preferrable and more accessible to most readers.

In our revise legend (Figure 4C) we explained now what the line thickness codes for. Using the Sankey diagram we wanted emphasize which KO cell lines caused the most dramatic change on YAP1 phosphosites (e.g. NF2, LATS1/2 and PTPN14 KO cells).

d. Panel d
i. The axis title "l/h" is unclear and not sufficiently explained. A more descriptive title would be recommended.

We spelled out l/h labels in the axis title: "Phosphorylation level log2(light/heavy)".

e. Panel f
i. Do the individual points represent replicates?
ii. Furthermore, it is unclear, whether the target proteins of the KO cells were removed from the data before doing the PCA.

Individual points represent replicates and we excluded the target proteins of the KO in the PCA plot. We rephrased the Figure 4F legend as follow:

"Principal component analysis based on both phosphorylation and interaction data (target proteins of the KO are removed from the data). Various mutants are highlighted in different colors and every dot represents a replicate condition."

5. Figure 5
a. Panel b
i. What is "[-]"

We removed as suggested by the reviewer.

b. Panel d
i. The authors could highlight proteins of interest here, e.g. LATS1 and CYR61
c. Panel e
i. Also here could highlight, e.g., PTPN14 and CYR61?

CYR61 and PTPN14 have not been identified in the proteome wide abundance dataset and therefore could not be annotated in Figure 5D,E. Consistent with previous protein abundance measurements (**FFR3**; Geiger et al.[3], pax-db.org) LATS1 expression is low in HEK293 cells and in the range of the limit of quantification for the dataset generated in Figure 5D,E. This is also reflected in the subthreshold adjusted *p*-values (log2FC = -1.2; *p*-value=0.9) in the volcano plot and the protein is therefore not highlighted (Dataset EV7). Please note that lack of LATS1 and PTPN14 expression in the corresponding KO cells has already been validated by more sensitive targeted analysis in the presented work (Appendix Figure S8A-E) as well as by immunodetection and qPCR by Plouffe et al[4].

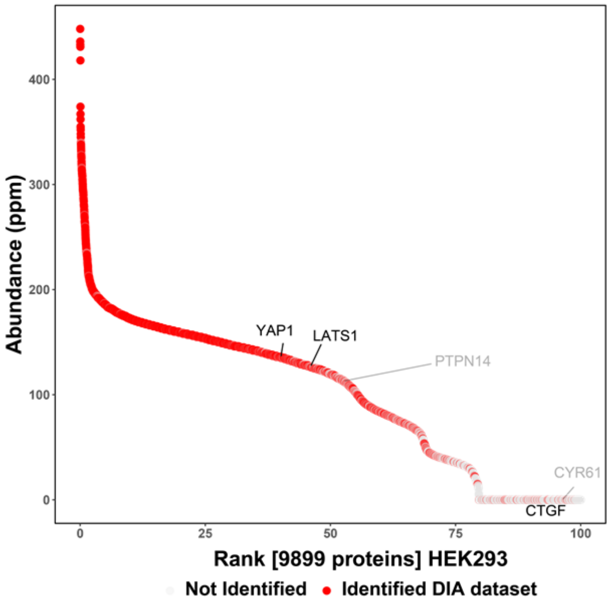

FFR3. Protein abundance distribution of reference HEK293 proteome (pax-db.org). Proteins identified in the acquired dataset (Dataset EV7) are annotated as red dot.

d. Panel f
i. It should be clarified more clearly in the figure that/if this represents whole proteome data

We acknowledge the reviewer for the comment, and we clarified the Figure 5F. In the plot we showed the intensity of CTGF gene across all cell lines with deletion mutants. To improve the clarity. we added a title for this plot: *"CTGF abundance across all deletion mutants".*

e. Panel g

i. Would the second PTPN14 peak suggest that there are other factors involved in recruiting LATS1 to YAP1 as well? Since there is a second peak with PTPN14 but without LATS1. Could this be due to differentially phosphorylated PTPN14?

It is possible. In **FFR4**, we have looked at different phosphosites of PTPN14 and indeed a phosphosite specific to the second PTPN14 peak is distinctly recognizable in the migration profile (cyan trace; PTPN14 phospho 642). This is an intriguing possibility, and more work will be required to test this interesting hypothesis.

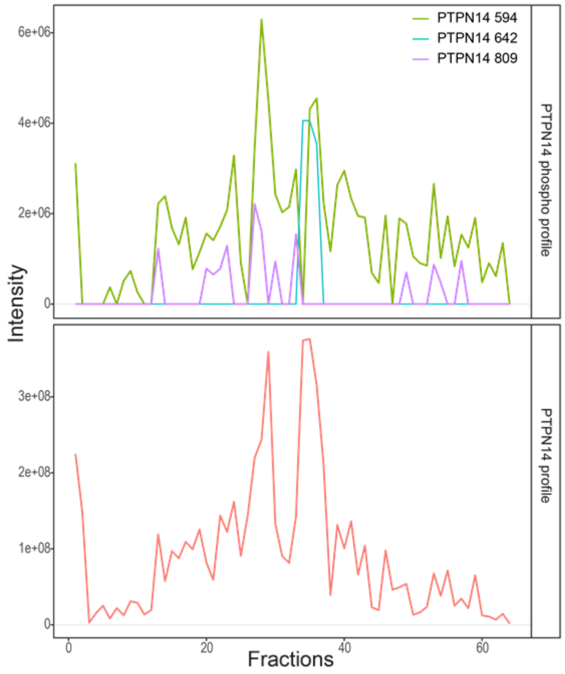

FFR4. Migration profile (raw data) of PTPN14 protein (lower panel) and relative phosphopeptides (upper panel). Data are extracted from Dataset EV2.

6. Figure S3 and S4
a. X axis missing

We added the label "Fraction" in the x axis of Appendix Figure S3 and S4.

7. Figure S5
a. iRT peptides should be explained more clearly

We corrected this Figure and added "external standards" to the y axis of Appendix Figure S5C.

8. Figure S6
a. Panel e
i. "identified exogenous interactor?"

We replaced 'exogenous interactor' with "Identified interactor in Strep-HA APMS' in the legend Appendix Figure S7E/G.

9. Figure S8
a. Panel c
i. Typo: "light petide"

We amended the typo in Appendix Figure S9C.

10. Figure S10
a. Panel c
i. Typo: "()LATS1"

We amended the typo in Appendix Figure S11C.

Additionally:
The finding that PTPN14 augments inhibition of YAP1 by LATS1/2 is interesting. Although potentially outside the scope of this study, the authors might want to consider doing rescue experiments with different PTPN14 mutants (e.g. catalytically dead) in the PTPN14 KO cell line to better understand the role of PTPN14 in the process.

To investigate the binding mechanism between the members of YAP1/LATS1/PTPN14 ternary complex, we added in the revised form of the manuscript two new datasets: we performed crosslinking experiment of purified Strep-HA YAP1 (Figure 5I) and AP-MS of Strep-HA YAP1 with WW domain mutations (Figure 5J). Crosslinking experiments identifies two lysine residues of PTPN14 (124 and 1043) in close proximity with YAP1 (less than 30Å), pinpointing the direct

interaction between PTPN14 and YAP1. Of note, these PTPN14 residues are not involved with LATS1 interaction (Figure 5I). Furthermore, the analysis of YAP1 mutations in WW domains reports a binding preference of PTPN14 for the second WW domain (Figure 5J). Combining these data, we suggest a topological model where the interaction between PTPN14 and YAP1 is direct and mediated by the N and C terminal regions of PTPN14 (the last one contains the phosphatase domain) with the second WW domain of YAP1.

Reviewer #2:

In the manuscript entitled "Phosphorylation linked complex profiling identified complexes required for Hippo signal integration", Uliana et al. used proteomics as an approach to investigate phosphorylation and interactome of YAP, a key effector protein in the Hippo pathway. In particular, they refined YAP-interacting proteins using a native PAGE system and revealed different YAP-associated protein complexes. Moreover, the authors compared YAP phosphorylation and interactome in a group of Hippo components-deficient cells, where they underscored the role of PTPN14, a previously known Hippo pathway regulator, in mediating LATS-induced YAP phosphorylation and inhibition. Overall, this study provides a new proteomic strategy for characterizing the relationship between protein phosphorylation and interactome for a protein of interest. However, the reviewer feels that applying this proteomic flow to analyzing YAP phosphorylation and interacting proteins was superficial, lacking experimental validation and mechanistic insights in advancing the current understanding of YAP regulation.

Specifically,

1. The novelty of the YAP-related interactome analysis presented here is low. First, the Hippo-related proteomic studies have been conducted by different labs a few years ago, who reported almost same interacting proteins for YAP as shown by the authors here. Second, among these published studies, similar phosphatase inhibitor treatment has been used to compare the Hippo PPI change, including the one for YAP (PMID: 24255178). Third, PTPN14 has already been known as a negative regulator of YAP by restricting YAP nuclear localization and promoting LATS activity (PMID: 25023289; PMID: 23613971; PMID: 22525271; PMID: 22948661).

We agree with the reviewer that the main novelty of the paper does not reside in the interactome nor the inhibitory role of PTPN14, but rather in the analysis and regulation of concurrent YAP1 complex formation. As highlighted in the discussion, we presented an approach to investigate i) effectors of phosphorylation events, ii) the causality between phosphorylation and the binding of interactors and iii) the resulting phenotype from the interaction rewiring (e.g. YAP1 translocation and co-activation of transcription activity upon deletion of PTPN14). Therefore, we highlight the scope of the paper in the discussion:

"In summary, we describe a strategy to simultaneously analyze functional relationship of two critical mechanisms of cell signaling: PTMs and complex formation. Using YAP1 as a model, we integrated multiple proteomics layers to study i) the role of phosphorylation for complex formation, ii) how phosphorylation and complex formation are controlled by known pathway effectors and iii) how phenotypes could emerge from perturbing these signaling mechanisms".

i) "First, the Hippo-related proteomic studies have been conducted by different labs a few years ago, who reported almost same interacting proteins for YAP as shown by the authors here."

Experiments presented in Figure 2 were not primarily intended to identify new interactors, but rather to establish a in depth high-confidence interactome and phosphoproteome reference data

set to be used as reference in the following experiments. We have emphasized this more clearly in the text by adding the sentence:

"Taken together, these data provide an extensive, quantitative map of YAP1 phosphosites/interactors and their responsiveness upon phosphatase inhibition, which serves as a reference library for subsequent experiments."

ii) "Second, among these published studies, similar phosphatase inhibitor treatment has been used to compare the Hippo PPI change, including the one for YAP (PMID: 24255178)."

We acknowledge that AP-MS studies also have used phosphatase inhibition in the past to uncover phosphorylation sensitive interactions[5]. However, to the best of our knowledge, only okadaic acid, but not vanadate has been used to study the plasticity of the YAP1 interactome and no thorough study on YAP1 phospho-proteome reorganization was presented.

iii) "Third, PTPN14 has already been known as a negative regulator of YAP by restricting YAP nuclear localization and promoting LATS activity (PMID: 25023289; PMID: 23613971; PMID: 22525271; PMID: 22948661)."

Besides providing orthogonal validation for the inhibitory role of PTNP14, we provided the first evidence for YAP1-PTPN14-LATS ternary complex formation (Figure 5G,H) and clearly showed that PTPN14 is required for the interaction between YAP1 and LATS (Figure 4H). Furthermore, in the revised version of the manuscript we now characterized the role of the two WW domains of YAP1 for ternary complex formation by performing the AP-MS with YAP1 WW domain mutants which revealed differential affinity of the two WW domains in binding PTPN14 and LATS (Figure 5J). Finally, we also analyzed YAP1-LATS-PTPN14 ternary complex formation by cross-linking MS which suggested direct interactions between all three proteins (Figure 5I). Taken together we believe that these novel biochemical insights significantly add to the understanding of the mechanism of YAP1 inhibition by PTPN14 and LATS1/2.

2. In Fig.2, profiling YAP phosphorylation sites was descriptive, lacking experimental validation and further mechanistic investigation. In addition to the known sites, what are the roles of the rest phosphorylation sites in regulating YAP? What kinases are required to phosphorylate these sites? Why would phosphorylation of these sites regulate the interaction between YAP and its binding partners (Fig. 2e)?

We agree with the reviewer that Figure 2 is descriptive; the signal from interactors and phosphosites upon perturbation is highly convoluted and it is hard to generate testable hypothesis (with the exception of the already known docking site of S127 for 14-3-3 proteins and TEADs) (Figure 2F). As describe above (review 2, major point 1), the main purpose of Figure 2 has been to establish a reference map for YAP1 phosphosites and phosphorylation dependent interactions for subsequent complex profiling (Figure 3) and functional experiments (Figure 3,4,5). In our revised manuscript we have now generated and analyzed several YAP1 phosphosite mutants to study the role of phosphorylation (Figure 3F,G). As a remarkable

example, we found that S367 strongly regulates all proteins of module 4 (Appendix Figure S5I) and it is required for binding with two proteins of module two (Figure 3H).

Extensive future experiments will be necessary to identify which of over 500 human kinases and 200 phosphatase control YAP phosphorylation under various physiological conditions. To this end we have studied the roles of LATS1, LATS2, STK3/4 LATS1/2 and PTPN14 by analyzing phosphorylation of endogenous YAP1 purified from the corresponding knock out cell lines. We found that LATS kinases are essential for phosphorylation of at least four sites primarily in the N-terminus of YAP1, overlapping with 14-3-3 binding site (Figure 4D).

3. Revealing different YAP-associated protein complexes by native PAGE is of interest (Fig. 3). Again, experimental validation of these distinct YAP-associated protein complexes is missing. In addition, how YAP phosphorylation affects these complex formation or switch (Fig. 3c) is needed to be addressed.

In the manuscript, we performed reciprocal pulldowns validating the existence of non-mutually exclusive interactions only for YAP1-LATS-PTPN14 (Figure 3C, module 3). Integration of the obtained BNPAGE co-migration data with public protein-protein interaction data show reciprocal interactions within the identified modules (Figure 3C). Furthermore, we have shown that members of the complexes identified by AP-BNPAGE more frequently interact with each other than with other YAP1 interactors (Figure EV2), as well as having enriched GO localization terms (Appendix Figure S5F) and being otherwise more related to one another (known protein complexes, homologous protein).

To identify potential regulatory roles for YAP1 phosphorylation in controlling YAP complex formation, in the revised manuscript, we have mutated several of the YAP1 phosphosites identified in specific YAP1 assemblies by AP-BNPAGE profiling and analyzed their effect on YAP1 complex formation by quantitative AP-MS. The results are summarized in Figure 3F-H and in the new text. Besides the known role of S127 in 14-3-3 protein binding we identified several other less studied N-terminal sites with similar roles to S127 and showed that the poorly studied YAP1 S367 phosphosite was not only co-migrating with TP53BP2 and CCDC85 in AP-BNPAGE (Figure 3C), but is also required for binding to these proteins (Figure 3H).

4. The data supporting current working model (Fig. 5i) were quite preliminary. Can PTPN14 directly bind LATS? Where is this PTPN14/LATS/ YAP complex localized in cells? Will the interaction between YAP and LATS be reduced in the PTPN14 KO cells as compared to that in wild-type cells? Since both LATS and PTPN14 contain PPxY motif that is required for their association with YAP (through YAP WW domain) (PMID: 18158288; PMID: 23613971; PMID: 22525271; PMID: 22948661), will PTPN14 and LATS compete each other for YAP binding? How is the PTPN14/LATS/YAP complex regulated in response to Hippo upstream signaling events (e.g., serum starvation, cell density, actin disruption)?

We thank the reviewer for this important set of questions regarding PTPN14-LATS-YAP1 complex formation. We have addressed these questions in our revised version by AP-MS analysis of the corresponding WW domains in YAP1 (Figure 5J) and by performing crosslinking MS on purified YAP1 complexes (Figure 5J). Results are discussed below and in the revised text.

i) "Can PTPN14 directly bind LATS?"

To test this hypothesis, we performed an AP-MS-XL experiment. Briefly, we performed affinity purification of ectopically expressed Strep-HA YAP1 followed by cross-linking reaction and analysis by mass spectrometry. Crosslinked peptides indicate the presence of lysine residues which are in close proximity (less than 30Å) and can be used as molecular ruler to pinpoint protein regions in the interaction interface and to indicate if the interaction is direct. Besides detection of PTPN14-YAP1 and LATS1-YAP1 cross linked peptides we also identified two crosslinked peptides between PTPN14 and LATS1 indicating direct interaction between these two proteins in YAP1 complexes. This new information is now included in Figure 5I.

ii) "Where is this PTPN14/LATS/ YAP complex localized in cells?".

Our data suggest the presence of PTPN14/LATS/ YAP1 ternary complex, but do not allow to identify its subcellular localization. To generate hypothesis regarding the YAP1-LATS1-PTPN14 complex localization we analyzed the proximity of PTPN14, LATS1 and YAP1 in a proteome wide proximity dependent biotiylation map[1]. LATS1 has been used as a bait and proximity data indicate a strong enrichment of prey proteins associated with cell-cell junctions (GO:0005911). Similarly, PTPN14 proximity data also suggest localization at cell junctions with good specificity using spatial analysis of functional enrichment analysis (SAFE). Cell junction have been also proposed for YAP1 but with lower confidence ($p= 0.00512$). Although more work is needed to corroborate this hypothesis, given this information we suggest a possible localization of PTPN14/LATS/YAP1 complex at cell junctions.

iii) "Will the interaction between YAP and LATS be reduced in the PTPN14 KO cells as compared to that in wild-type cells?"

The interaction between YAP1 and LATS1 is reduced in PTPN14 KO cells. We performed IP-MS of endogenous YAP1 in PTPN14 and LATS1/2 deficient cell line and we found that PTPN14 KO reduces the binding between YAP1 and LATS1, but LATS1/2 KO does not decrease the amount of PTNP14 associated with YAP1 (Figure 4H., left and right, respectively). This data support a model in which PTPN14 is essential for the interaction between YAP1 and LATS1 and the subsequent regulation of YAP1 by the kinase.

iv) "Will PTPTN14 and LATS compete each other for YAP binding?"

This is an interesting question and we thank the reviewer for raising this topic. We aimed to tackle this problem by asking whether LATS1 and PTPN14 binding to YAP1 compete for the same binding domains on YAP1. Previous data showed that the two YAP1 WW domains are required for PTPN14 binding[6-8]. In our revised version we therefore analyzed WW domain mutants of YAP1 by AP-MS. Mutation in the YAP1 WW1 domain basically abolished LATS1

binding whereas PTPN14 could still bind (although at reduced levels compared to WT YAP1). In WW2 mutant on the other hand, PTPN14 levels were strongly reduced in YAP1 complexes whereas LATS1 was still able to bind YAP1. As expected, mutating both WW domains abolished both, LATS1 as well as PTPN14 binding to YAP1. These new results suggest preferential binding of LATS1 to WW1 whereas PTPN14 would favor WW2. This in turn would be consistent with a model where LATS1 and PTPN14 can bind in a non-competitive fashion and that the ternary complex formation is organized by the two WW domains. Such a model is further supported by AP-MS-XL experiment on purified YAP1 complexes. These new results are shown now in Figure 5i and j and discussed in the revised text.

v) "How is the PTPN14/LATS/YAP complex regulated in response to the Hippo upstream signaling events? (e.g., serum starvation, cell density, actin disruption)"

To answer this question (in particular the effect of cell-density on PTPN14/LATS1/YAP1 complex), we combined prior knowledge with the datasets acquired in this project. Reduced cell density affects cell-cell contacts which represent an established Hippo upstream signal for YAP1 activation[9]. *Wang et al.*[2] reported that, at low cell density, PTPN14 is degraded by the ubiquitination machinery. In agreement with these findings, *Hauri et al.*[10] previously observed reduced binding of PTPN14 and LATS1 to YAP1 and activation of YAP1 under low cell density conditions. We have obtained similar results in PTPN14 KO cells where we found reduced LATS1 in YAP1 complexes (Figure 4H) as well as hypo-phosphorylation and subsequent activation of YAP1 and its target genes (Figure 5A-H). Given also the primary localization of PTPN14 at cell-cell junctions (see response 4.ii to Reviewer 2 and response 3 to Reviewer 3, **FFR6**), these results agree with a key role of PTPN14 as regulator of YAP1-LATS1 interaction (Figure 4H) via a ternary complex formation (Figure 5G-J) upon Hippo signaling via cell-cell contacts. When PTPN14 is limited, the interaction between YAP1 and LATS1 is reduced (Figure 4H) which, in turn, leads to YAP1 hypo-phosphorylation (Figure 4D, 5A), nuclear translocation (Figure 5B) and transcription of YAP1 target genes involved in cell proliferation (e.g. CTGF, CYR61) (Figure 5C-F).

****
Pre-decision Cross-commenting:

Reviewer #1
We agree with the points raised by ref 2 and think they should be addressed before publishing in MSB.

Reviewer #2
I agree with Reviewer #1's comments and feel they are constructive and in details.
Actually, many previous studies have performed mass spec analyses of YAP and also characterized PTPN14 in Hippo pathway regulation. To move the field forward, if the authors decide to functionally validate their new mass spec findings and elucidate the newly discovered role of PTPN14 in the Hippo pathway as suggested, I am ok with a major revision decision.

Referee #3

Uliana and colleagues introduce a multi-layered mass-spectrometry-based proteomics strategy to establish interdependencies between protein phosphorylations and protein-protein interactions. These comprise charting the phosphorylation landscape of a target protein, analyzing proteoform-dependent protein complex compositions and analyzing proteoforms and complexes in steady state and upon pharmacological phosphatase inhibition. Application of this workflow to YAP1 identified a non-canonical regulation of complex formation with the serine/ threonine kinase LATS1/2 controlled by the tyrosine phosphatase PTPN14.
The manuscript is very well-written and major claims are supported by data. However, few points require further clarification or analysis to evaluate the capabilities of the workflow and interpretation of the data.

Major points:

1) Is high protein expression a prerequisite for application of this workflow or can it be used to study low-abundant/ tightly regulated protein complexes?

Besides YAP1 which has a median abundance in HEK293 cells (padx-db.org, **FFR3**), we also recently succeeded to profile, using AP-BNPAGE, sub-complexes of an endogenous membrane signalosome (TNFR-SC). To overcome problems with low intensity signals we have applied in this case targeted MS and to quantify peptides in the zeptomoles range[11]. Therefore, we think that our method in principle can be applied to a wide range of proteins for in depth complex profiling and will be particularly useful for proteins which are known to undergo multisite phosphorylation and bind to many cellular proteins.

2) What is the expression level of YAK1 in the herein used HEK293 cells? Is it comparable to endogenous YAP1 levels?

The expression level of ectopically expressed YAP1 in HEK293 is about 10 times higher than the endogenous expression level. We analyzed ectopic and endogenous YAP1 levels by immunoblot in **FFR5**.

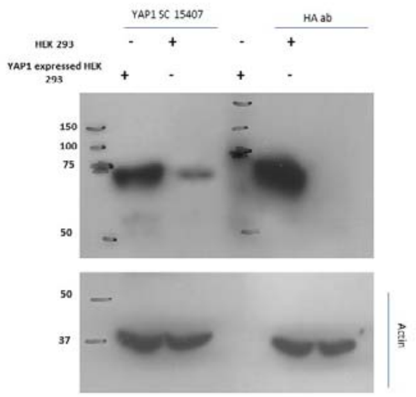

FFR5. Immunoblot comparison of exogenous and endogenous YAP1 levels using YAP1 antibody (SC 15407, Santa Cruz Biotechnology) and anti HA antibodies (HA.11,901513, BioLegend)

3) The manuscript will greatly benefit from comparisons to other current interaction proteomics approaches with focus on subcellular resolution, e.g. "A proximity-dependent biotinylation map of a human cell", Go CD et al., Nature 2021, or "Systematic identification of Protein Phosphorylation-Mediated Interactions", Floyd B et al., JPR 2021 or others.

We thank the reviewer for pointing a comparison of our data with these important resources. We integrated our results with the BioID data generated to characterize the human cell map[1] We used this resource to i) provide additional validation regarding the presence of the ternary complex PTPN14/LATS1/YAP1 and ii) to infer the possible localization of the complex (see response 4.ii to reviewer 2). Browsing through this dataset, we found that among all 192 BioID experiments, LATS1 and SYNE3 baits selectively purify (Saint score = 1) YAP1/PTPN14 and LATS1/PTPN14 respectively (**FFR6**, panel A). We added this information in the main text. Gene ontology analysis of LATS1 BioID interactors reveals an enrichment of prey proteins associated with cell-cell junctions (GO:0005911) (**FFR6**, panel B).

Regarding the paper of Floyd et al., in the discussion we cited phospho-DIFFRAC as a valid approach to investigate phosphorylation dependence of proteins assemblies. This global methodology is based on a co-fractionation combined with lambda protein phosphatase treatment to identify protein elution profile changes (DIFFRAC score). Unfortunately, the resolution and sensitivity of the global proteome profile doesn't allow to assign multiple complexes and the corresponding phosphopeptides for YAP1 complexes.

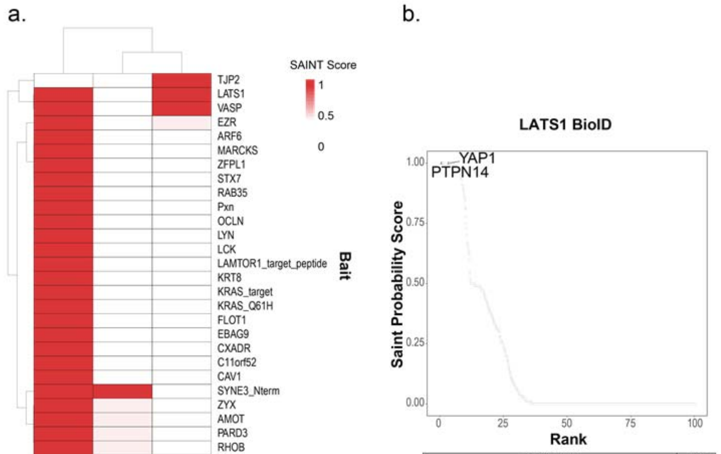

GO term (CC)	pval
cell periphery	7.95E-24
plasma membrane	5.95E-22
cell junction	5.57E-15
cell-cell junction	1.98E-11
plasma membrane part	6.95E-09
plasma membrane bounded cell projection	3.39E-07
plasma membrane region	6.32E-07
cell projection	7.90E-07
anchoring junction	2.37E-06
adherens junction	8.20E-06

FFR6. Re-analysis of cell map database (cell-map.org). a. The heatmap reports BIOID baits in which YAP1, LATS1 or PTPN14 were identified with a cutoff of SAINT Probability Score > 0.9. LATS1 BioID experiment selectively purifies YAP1 and PTPN14 while SYNE3 bait only PTPN14 and LATS1. b. Distribution of SAINT Probability score for LATS1 BioID experiments. YAP1 and PTPN14 are marked in bold. The table below the graph reports gene ontology analysis of the purified interactors.

4) Although impressive data can be generated, the workflow requires a significant amount of cells as starting material and significant MS resources, which - I assume - restricts it to investigations of cell lines as model systems and dedicated MS labs. The authors should mention the requirements clearly and discuss limitations that come with it.

We agree that our method as most MS based technique is limited by the limit of MS based quantitation. Although some of these limitations can be overcome by targeted MS (see response 1 to Reviewer 3) we included these caveats in the revised text. Specifically, we added the following sentences in the discussion:

*"This approach, although successful in resolving the modular organization of YAP1 interactome, is limited by experimental and data analysis caveats that need to be emphasized. The experiment discussed required large amounts of cells (300*10^6 cells per replicate) and considerable MS acquisition time (nearly one week of measurements). In addition, the separation by BNPAGE may disrupt the most labile interactions (as described for the assembly intermediates described by modules 5, 7 and 8 and in the case of 14-3-3 proteins, which co-migrate in a unique cluster at low molecular weight). Data analysis is challenged by co-migrating complexes that might lead to convoluted modules, although to a lesser extent than in total protein profiling experiments. Furthermore, under-sampling of low abundant phosphopeptides might compromise characterization of proteoforms and their assignment to a given module. "*

5) The workflow used a range of custom-generated antibodies against various YAP1 P-sites. This could be a hinderance to the transferability of the workflow to other protein complexes. Please discuss.

In this study, we have generated and used exclusively an antibody against the YAP1 C-terminal region to perform endogenous immune-purifications. Subsequent study of YAP1 phosphorylation sites have been performed using DDA MS or targeted MS analysis which can be generally applied for other proteins.

6) The protein tyrosine kinase ABL can phosphorylate YAP1. Similar to LATS1/2, would a lack of ABL also result in a change of proteoform complexes or is the role of ABL in non-canonical YAP1 phosphorylation irrelevant?

We thank the reviewer for this comment. We have not generated an ABL KO, but similar experiments to those carried out on Figure 4 could be done for ABL and other kinases in future studies.

7) YAP1 can be ubiquinated by SCF(beta-TCRP) E3 ubiquitin ligase. Is the workflow also suitable to screen for other PTMs than phosphorylations? Could ubiquitination alter YAP1 complex composition? It would suffice to discuss the scope of the workflow with regard to other PTMs than Phosphorylations.

Yes, in principle this workflow can be applied to other PTMs. YAP1 was chosen because it is known to carry a high number of phosphosites (Appendix Figure S1A). We have attempted to look for additional modifications including ubiquitination, acetylation, methylation in our datasets, but unfortunately the resulting identifications too sparse and with lower confidence of identification. A different model protein or protocols to preserve labile PTMs (e.g. by including DUB or proteasome inhibitors) may be needed to address the role of other PTMs on the rewiring of protein interaction. Based on this comment proposed by the reviewer we adapted our concluding remarks at the end of the manuscript:

"The steps described in this manuscript can be adapted to study the effect of different types of PTMs (i.e. ubiquitination) for a wide range of proteins that partition into multiple complexes."

Minor points:

1) The title includes the words 'complex' and 'complexes'. 'Complex' can be removed or replaced by a synonym to facilitate intuitive understanding.

We replaced ‘complexes’ with the synonym ‘assemblies’.

2) In the abstract, there's a mention of isoform complexes. What is meant is presumably proteoform complexes.

We removed ‘isoform’, as we recognize it may be ambiguous.

Bibliography:

1. Go, C. D. *et al.* A proximity-dependent biotinylation map of a human cell. *Nature* **595**, 120–124 (2021).
2. Wang, W. *et al.* PTPN14 is required for the density-dependent control of YAP1. *Genes Dev.* **26**, 1959–1971 (2012).
3. Geiger, T., Wehner, A., Schaab, C., Cox, J. & Mann, M. Comparative Proteomic Analysis of Eleven Common Cell Lines Reveals Ubiquitous but Varying Expression of Most Proteins. *Mol. Cell. Proteomics MCP* **11**, M111.014050 (2012).
4. Plouffe, S. W. *et al.* Characterization of Hippo Pathway Components by Gene Inactivation. *Mol. Cell* **64**, 993–1008 (2016).
5. Couzens, A. L. *et al.* Protein Interaction Network of the Mammalian Hippo Pathway Reveals Mechanisms of Kinase-Phosphatase Interactions. *Sci. Signal.* **6**, rs15–rs15 (2013).
6. Michaloglou, C. *et al.* The Tyrosine Phosphatase PTPN14 Is a Negative Regulator of YAP Activity. *PLOS ONE* **8**, e61916 (2013).
7. Schuchardt, B. J. *et al.* Ligand binding to WW tandem domains of YAP2 transcriptional regulator is under negative cooperativity. *FEBS J.* **281**, 5532–5551 (2014).
8. Vargas, R. E. *et al.* Elucidation of WW domain ligand binding specificities in the Hippo pathway reveals STXBP4 as YAP inhibitor. *EMBO J.* **39**, e102406 (2020).
9. Karaman, R. & Halder, G. Cell Junctions in Hippo Signaling. *Cold Spring Harb. Perspect. Biol.* **10**, a028753 (2018).
10. Hauri, S. *et al.* Interaction proteome of human Hippo signaling: modular control of the co-activator YAP1. *Mol. Syst. Biol.* **9**, 713 (2013).
11. Ciuffa, R. *et al.* Novel biochemical, structural, and systems insights into inflammatory signaling revealed by contextual interaction proteomics. *Proc. Natl. Acad. Sci.* **119**, e2117175119 (2022).

1st Revision - Editorial Decision **20th Feb 2023**

Thank you for sending us your revised manuscript. We have now received comments from the three referees who agreed to evaluate your study. As you will see below, the referees are overall satisfied with the modifications and think the study is now suitable for publication.

Reviewer #1:

I am happy with how comments have been addressed and have no further issues

Reviewer #2:

I thank the authors for their efforts to address my previous questions and critiques.

As for the revision, most validations and functional analyses of the identified YAP phosphorylation sites and YAP-LATS1-PTPN14 complex were performed using MS (e.g., IP-crosslink-MS) instead of classic biochemistry, molecular biology and cell biology-related approaches, which dampened the enthusiasm of the reviewer.

As a MS-based systems biology study, the provided data support its acceptance for publication.

Reviewer #3:

The authors have addressed the raised points well. I am in favor of a publication in MSB.

All editorial and formatting issues were resolved by the authors.

2nd Revision - Editorial Decision **22nd Feb 2023**

Thank you again for sending us your revised manuscript. We are now satisfied with the modifications made and I am pleased to inform you that your paper has been accepted for publication.

EMBO Press Author Checklist

Corresponding Author Name: Matthias Gstaiger and Federico Uliana
Journal Submitted to: Molecular System Biology
Manuscript Number: MSB-2022-11024RR

USEFUL LINKS FOR COMPLETING THIS FORM
The EMBO Journal - Author Guidelines
EMBO Reports - Author Guidelines
Molecular Systems Biology - Author Guidelines
EMBO Molecular Medicine - Author Guidelines

Reporting Checklist for Life Science Articles (updated January
This checklist is adapted from Materials Design Analysis Reporting (MDAR) Checklist for Authors. MDAR establishes a minimum set of requirements in transparent reporting in the life sciences (see Statement of Task: 10.31222/osf.io/9sm4x). Please follow the journal's guidelines in preparing your
Please note that a copy of this checklist will be published alongside your article.

Abridged guidelines for figures
1. Data
The data shown in figures should satisfy the following conditions:
- the data were obtained and processed according to the field's best practice and are presented to reflect the results of the experiments in an accurate and unbiased manner.
- ideally, figure panels should include only measurements that are directly comparable to each other and obtained with the same assay.
- plots include clearly labeled error bars for independent experiments and sample sizes. Unless justified, error bars should not be shown for technical
- if n<5, the individual data points from each experiment should be plotted. Any statistical test employed should be justified.
- Source Data should be included to report the data underlying figures according to the guidelines set out in the authorship guidelines on Data

2. Captions
Each figure caption should contain the following information, for each panel where they are relevant:
- a specification of the experimental system investigated (eg cell line, species name).
- the assay(s) and method(s) used to carry out the reported observations and measurements.
- an explicit mention of the biological and chemical entity(ies) that are being measured.
- an explicit mention of the biological and chemical entity(ies) that are altered/varied/perturbed in a controlled manner.
- the exact sample size (n) for each experimental group/condition, given as a number, not a range;
- a description of the sample collection allowing the reader to understand whether the samples represent technical or biological replicates (including how many animals, litters, cultures, etc.).
- a statement of how many times the experiment shown was independently replicated in the laboratory.
- definitions of statistical methods and measures:
  - common tests, such as t-test (please specify whether paired vs. unpaired), simple χ2 tests, Wilcoxon and Mann-Whitney tests, can be unambiguously identified by name only, but more complex techniques should be described in the methods section;
  - are tests one-sided or two-sided?
  - are there adjustments for multiple comparisons?
  - exact statistical test results, e.g., P values = x but not P values < x;
  - definition of 'center values' as median or average;
  - definition of error bars as s.d. or s.e.m.

Please complete ALL of the questions below.
Select "Not Applicable" only when the requested information is not relevant for your study.

Materials

Newly Created Materials	Information included in the manuscript?	In which section is the information available? (Reagents and Tools Table, Materials and Methods, Figures, Data Availability Section)
New materials and reagents need to be available; do any restrictions apply?	Yes	Material and Methods

Antibodies	Information included in the manuscript?	In which section is the information available? (Reagents and Tools Table, Materials and Methods, Figures, Data Availability Section)
For **antibodies** provide the following information:<br>- Commercial antibodies: RRID (if possible) or supplier name, catalogue number and or/clone number<br>- Non-commercial: RRID or citation	Yes	Material and Methods

DNA and RNA sequences	Information included in the manuscript?	In which section is the information available? (Reagents and Tools Table, Materials and Methods, Figures, Data Availability Section)
**Short novel DNA or RNA including primers, probes**: provide the sequences.	Yes	Material and Methods

Cell materials	Information included in the manuscript?	In which section is the information available? (Reagents and Tools Table, Materials and Methods, Figures, Data Availability Section)
**Cell lines:** Provide species information, strain. Provide accession number in repository **OR** supplier name, catalog number, clone number, and/**OR** RRID.	Yes	Material and Methods
**Primary cultures:** Provide species, strain, sex of origin, genetic modification status.	Not Applicable	
Report if the cell lines were recently **authenticated** (e.g., by STR profiling) and tested for mycoplasma contamination.	Not Applicable	

Experimental animals	Information included in the manuscript?	In which section is the information available? (Reagents and Tools Table, Materials and Methods, Figures, Data Availability Section)
**Laboratory animals or Model organisms:** Provide species, strain, sex, age, genetic modification status. Provide accession number in repository **OR** supplier name, catalog number, clone number, **OR** RRID.	Not Applicable	
**Animal observed in or captured from the field:** Provide species, sex, and age where possible**.**	Not Applicable	
Please detail **housing and husbandry conditions**.	Not Applicable	

Plants and microbes	Information included in the manuscript?	In which section is the information available? (Reagents and Tools Table, Materials and Methods, Figures, Data Availability Section)
**Plants:** provide species and strain, ecotype and cultivar where relevant, unique accession number if available, and source (including location for collected wild specimens).	Not Applicable	
**Microbes:** provide species and strain, unique accession number if available, and source.	Not Applicable	

Human research participants	Information included in the manuscript?	In which section is the information available? (Reagents and Tools Table, Materials and Methods, Figures, Data Availability Section)
If collected and within the bounds of privacy constraints report on age, sex and gender or ethnicity for all study participants.	Not Applicable	

Core facilities	Information included in the manuscript?	In which section is the information available? (Reagents and Tools Table, Materials and Methods, Figures, Data Availability Section)
If your work benefited from core facilities, was their service mentioned in the acknowledgments section?	Yes	Acknowledgements

Design

Study protocol	Information included in the manuscript?	In which section is the information available? (Reagents and Tools Table, Materials and Methods, Figures, Data Availability Section)
If study protocol has been **pre-registered, provide DOI in the manuscript**. For clinical trials, provide the trial registration number **OR** cite DOI.	Not Applicable	
Report the **clinical trial registration number** (at ClinicalTrials.gov or equivalent), where applicable.	Not Applicable	

Laboratory protocol	Information included in the manuscript?	In which section is the information available? (Reagents and Tools Table, Materials and Methods, Figures, Data Availability Section)
Provide DOI OR other citation details if **external detailed step-by-step protocols** are available.	Yes	Material and Methods

Experimental study design and statistics	Information included in the manuscript?	In which section is the information available? (Reagents and Tools Table, Materials and Methods, Figures, Data Availability Section)
Include a statement about **sample size** estimate even if no statistical methods were used.	Yes	Material and Methods
Were any steps taken to minimize the effects of subjective bias when allocating animals/samples to treatment (e.g. **randomization procedure**)? If yes, have they been described?	Not Applicable	
Include a statement about **blinding** even if no blinding was done.	Not Applicable	
Describe **inclusion/exclusion criteria** if samples or animals were excluded from the analysis. Were the criteria pre-established?<br><br>If sample or data points were omitted from analysis, report if this was due to attrition or intentional exclusion and provide justification.	Not Applicable	
For every figure, are **statistical tests** justified as appropriate? Do the data meet the assumptions of the tests (e.g., normal distribution)? Describe any methods used to assess it. Is there an estimate of variation within each group of data? Is the variance similar between the groups that are being statistically compared?	Yes	Material and Methods, Figure captions

Sample definition and in-laboratory replication	Information included in the manuscript?	In which section is the information available? (Reagents and Tools Table, Materials and Methods, Figures, Data Availability Section)
In the figure legends: state number of times the experiment was **replicated** in laboratory.	Yes	Figure legends
In the figure legends: define whether data describe **technical or biological replicates**.	Yes	Figure legends

Ethics

Ethics	Information included in the manuscript?	In which section is the information available? (Reagents and Tools Table, Materials and Methods, Figures, Data Availability Section)
Studies involving **human participants**: State details of **authority granting ethics approval** (IRB or equivalent committee(s), provide reference number for approval.	Not Applicable	
Studies involving **human participants**: Include a statement confirming that **informed consent** was obtained from all subjects and that the experiments conformed to the principles set out in the WMA Declaration of Helsinki and the Department of Health and Human Services Belmont Report.	Not Applicable	
Studies involving **human participants:** For publication of **patient photos**, include a statement confirming that consent to publish was obtained.	Not Applicable	
Studies involving experimental **animals**: State details of **authority granting ethics approva**l (IRB or equivalent committee(s), provide reference number for approval. Include a statement of compliance with ethical regulations.	Not Applicable	
Studies involving **specimen and field samples:** State if relevant **permits** obtained, provide details of authority approving study; if none were required, explain why.	Not Applicable	

Dual Use Research of Concern (DURC)	Information included in the manuscript?	In which section is the information available? (Reagents and Tools Table, Materials and Methods, Figures, Data Availability Section)
Could your study fall under dual use research restrictions? Please check biosecurity documents and list of **select agents and toxins** (CDC): https://www.selectagents.gov/sat/list.htm	Not Applicable	
If you used a select agent, is the security level of the lab appropriate and reported in the manuscript?	Not Applicable	
If a study is subject to dual use research of concern regulations, is the name of the **authority granting approval and reference number** for the regulatory approval provided in the manuscript?	Not Applicable	

Reporting

The MDAR framework recommends adoption of discipline-specific guidelines, established and endorsed through community initiatives. Journals have their own policy about requiring specific guidelines and recommendations to complement MDAR.

Adherence to community standards	Information included in the manuscript?	In which section is the information available? (Reagents and Tools Table, Materials and Methods, Figures, Data Availability Section)
State if relevant guidelines or checklists (e.g., **ICMJE, MIBBI, ARRIVE, PRISMA**) have been followed or provided.	Not Applicable	
For **tumor marker prognostic studies,** we recommend that you follow the **REMARK** reporting guidelines (see link list at top right). See author guidelines, under 'Reporting Guidelines'. Please confirm you have followed these guidelines.	Not Applicable	
For **phase II and III randomized controlled trials**, please refer to the **CONSORT** flow diagram (see link list at top right) and submit the CONSORT checklist (see link list at top right) with your submission. See author guidelines, under 'Reporting Guidelines'. Please confirm you have submitted this list.	Not Applicable	

Data Availability

Data availability	Information included in the manuscript?	In which section is the information available? (Reagents and Tools Table, Materials and Methods, Figures, Data Availability Section)
Have **primary datasets** been deposited according to the journal's guidelines (see 'Data Deposition' section) and the respective accession numbers provided in the Data Availability Section?	Yes	Material and Methods and Data availability section
Were **human clinical and genomic datasets** deposited in a public access-controlled repository in accordance to ethical obligations to the patients and to the applicable consent agreement?	Not Applicable	
Are **computational models** that are central and integral to a study available without restrictions in a machine-readable form? Were the relevant accession numbers or links provided?	Not Applicable	
If publicly available data were reused, provide the respective **data citations in the reference list**.	Not Applicable	
